# Repurposing of tamoxifen ameliorates CLN3 and CLN7 disease phenotype

**DOI:** 10.15252/emmm.202013742

**Published:** 2021-08-19

**Authors:** Chiara Soldati, Irene Lopez‐Fabuel, Luca G Wanderlingh, Marina Garcia‐Macia, Jlenia Monfregola, Alessandra Esposito, Gennaro Napolitano, Marta Guevara‐Ferrer, Anna Scotto Rosato, Einar K Krogsaeter, Dominik Paquet, Christian M Grimm, Sandro Montefusco, Thomas Braulke, Stephan Storch, Sara E Mole, Maria A De Matteis, Andrea Ballabio, Julio L Sampaio, Tristan McKay, Ludger Johannes, Juan P Bolaños, Diego L Medina

**Affiliations:** ^1^ Telethon Institute of Genetics and Medicine (TIGEM), Pozzuoli Naples Italy; ^2^ Institute of Functional Biology and Genomics CSIC University of Salamanca Salamanca Spain; ^3^ Centro de Investigación Biomédica en Red sobre Fragilidad y Envejecimiento Saludable (CIBERFES) Instituto de Salud Carlos III Madrid Spain; ^4^ Institute of Biomedical Research of Salamanca University Hospital of Salamanca CSIC University of Salamanca Salamanca Spain; ^5^ Medical Genetics Unit Department of Medical and Translational Science Federico II University Naples Italy; ^6^ School of Healthcare Science Manchester Metropolitan University Manchester UK; ^7^ Faculty of Medicine Walther Straub Institute of Pharmacology and Toxicology Ludwig‐Maximilians University Munich Germany; ^8^ Institute for Stroke and Dementia Research (ISD) University Hospital LMU Munich Munich Germany; ^9^ Munich Cluster for Systems Neurology (SyNergy) Munich Germany; ^10^ Department Osteology & Biomechanics University Medical Center Hamburg‐Eppendorf Hamburg Germany; ^11^ University Children's Research@Kinder‐UKE University Medical Center Hamburg‐Eppendorf Hamburg Germany; ^12^ Medical Research Council Laboratory for Molecular Cell Biology and UCL Great Ormond Street Institute of Child Health University College London London UK; ^13^ Department of Molecular Medicine and Medical Biotechnology University of Napoli Federico II Naples Italy; ^14^ Baylor College of Medicine Houston TX USA; ^15^ Jan and Dan Duncan Neurological Research Institute Texas Children's Hospital Houston TX USA; ^16^ Cellular and Chemical Biology Department Institut Curie, U1143 INSERM, UMR3666 CNRS PSL Research University Paris France

**Keywords:** CLN3, CLN7, high content imaging screening, tamoxifen, TFEB, Genetics, Gene Therapy & Genetic Disease, Neuroscience, Organelles

## Abstract

Batten diseases (BDs) are a group of lysosomal storage disorders characterized by seizure, visual loss, and cognitive and motor deterioration. We discovered increased levels of globotriaosylceramide (Gb3) in cellular and murine models of CLN3 and CLN7 diseases and used fluorescent‐conjugated bacterial toxins to label Gb3 to develop a cell‐based high content imaging (HCI) screening assay for the repurposing of FDA‐approved compounds able to reduce this accumulation within BD cells. We found that tamoxifen reduced the lysosomal accumulation of Gb3 in CLN3 and CLN7 cell models, including neuronal progenitor cells (NPCs) from CLN7 patient‐derived induced pluripotent stem cells (iPSC). Here, tamoxifen exerts its action through a mechanism that involves activation of the transcription factor EB (TFEB), a master gene of lysosomal function and autophagy. *In vivo* administration of tamoxifen to the CLN7^Δex2^ mouse model reduced the accumulation of Gb3 and SCMAS, decreased neuroinflammation, and improved motor coordination. These data strongly suggest that tamoxifen may be a suitable drug to treat some types of Batten disease.

## Graphical abstract

The neuronal ceroid lipofuscinoses (NCL), commonly known as Batten disease (BD), are a group of recessively inherited fatal diseases of the nervous system that typically arise in childhood. There is neither cure nor drugs to revert the course of these diseases.

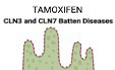

**The paper explained**

**Problem**

Batten disease (BD) is a group of fatal neurodegenerative rare diseases. There is neither cure nor drugs to revert the course of these diseases. Systemic administration of small‐molecule therapeutics may represent an alternative to existing protein and gene therapies, which cannot readily cross the blood–brain barrier to reach the brain.

**Results**

We have discovered a pathological accumulation of the lipid globotriaosylceramide (Gb3) in cellular and murine models of two types of BD, CLN3, and CLN7. By using this novel disease hallmark, we developed a cell‐based assay that identified the FDA‐approved compound tamoxifen. *In vitro* and *in vivo* studies showed that tamoxifen ameliorates BD using human disease cells and a murine model of CLN7.

**Impact**

This study highlights the relevance of combining cell biology approaches with the repurposing of approved drugs to identify small‐molecule therapeutics to treat rare diseases. Our data strongly suggest that the clinical compound tamoxifen may be a suitable drug to treat some types of Batten disease.

## Introduction

The neuronal ceroid lipofuscinoses (NCL), commonly known as Batten disease (BD), are a group of recessively inherited fatal diseases of the nervous system which typically arise in childhood. Neurodegenerative disorders in children are rare, with BD the most frequent; the incidence rates of the entire group of 13 genetically distinct NCL varies between 1:14,000 and 1:67,000, (Haltia & Goebel, 2013; Kauss *et al*, 2020; Mole *et al*, 2019). Current palliative treatment can reduce some symptoms but cannot prevent progressive CNS degeneration. At the cellular level, alterations in BD cells include lysosomal accumulation of toxic metabolites, lipid trafficking impairment, perturbed signalling, disturbed calcium homeostasis in the endoplasmic reticulum, and activation of the unfolded protein response (UPR) (Boustany, 2013; Mole, 2014). Genes involved in human NCLs encode soluble lysosomal enzymes (CLN1/PPT1, CLN2/TPP1, CLN10/CTSD, CLN13/CTSF), a soluble lysosomal protein that can be secreted (CLN5), a protein in the secretory pathway (CLN11/GRN), two cytoplasmic proteins that also peripherally associate with membranes (CLN4/DNAJC5, CLN14/KCTD7), and transmembrane proteins located in the endoplasmic reticulum (CLN6), endoplasmic reticulum/*cis* Golgi (CLN8) and in lysosomes (CLN3, CLN7, CLN12) (Mole & Cotman, 2015; Huber & Mathavarajah, 2018). Despite the identification of causative genes of BD, protein functional understanding remains elusive making it challenging to target therapeutic drugs by intelligent design (Mole, 2014; Kauss *et al*, 2020). In BD, systemic administration of small‐molecule therapeutics is an attractive alternative to existing protein and gene therapies, which cannot readily cross the blood‐brain barrier to reach the CNS. The repurposing approach of known drugs, able to correct pathological hallmarks of BD, would represent a significant therapeutic advance in the treatment of these disorders and benefit from an acceleration of their translation to clinics.

Here, we combine cell‐based phenotypic screening and repurposing of FDA drugs for the identification of correctors of lysosomal storage in CLN3 and CLN7 cellular models of BD. CLN3 disease (MIM # 204200) represents the most common form of NCL worldwide, whereas CLN7 disease (MIM # 610951) is one of the most prevalent BD in southern and Mediterranean Europe. We found a significant endogenous accumulation of the glycosphingolipid globotriaosylceramide (Gb3) (Welford *et al*, 2018) within the lysosomes of human ARPE‐19 cells depleted of *CLN3* or *CLN7* genes by CRISPR genome editing. Gb3 was also found in human juvenile CLN3 patient fibroblasts, neuronal progenitor cells (NPCs) derived from CLN7 patient iPSCs, and neurons in brain tissues from both Cln3^Δex7/8^ and Cln7^Δex2^ mutant mice, suggesting that Gb3 accumulation is part of the pathological storage in these diseases. By using fluorescent‐conjugated bacterial toxins to label Gb3, we developed a cell‐based high content imaging (HCI) screening assay for the repurposing of FDA‐approved compounds able to reduce the accumulation of Gb3 within the lysosomes of BD cells. We found that tamoxifen significantly reduces the intracellular accumulation of Gb3 in CLN3 and CLN7 cell models through a mechanism that is independent of oestrogen receptors but involves activation of the transcription factor EB (TFEB), a master gene of lysosomal function and autophagy (Sardiello *et al*, 2009). TFEB activation by tamoxifen is triggered by lysosomotropic‐mediated inhibition of mTORC1. Furthermore, *in vivo* administration of tamoxifen significantly rescued Cln7^Δex2^ mice from brain cortex Gb3 accumulation, reducing SCMAS storage, hindlimb clasping, and motor discoordination. These data indicate that Gb3 is a novel biomarker for CLN3 and CLN7 diseases, and tamoxifen may be a suitable drug for their treatment.

## Results

### Pathological accumulation of Gb3 in CLN3 and CLN7 diseases

**Figure 1:**
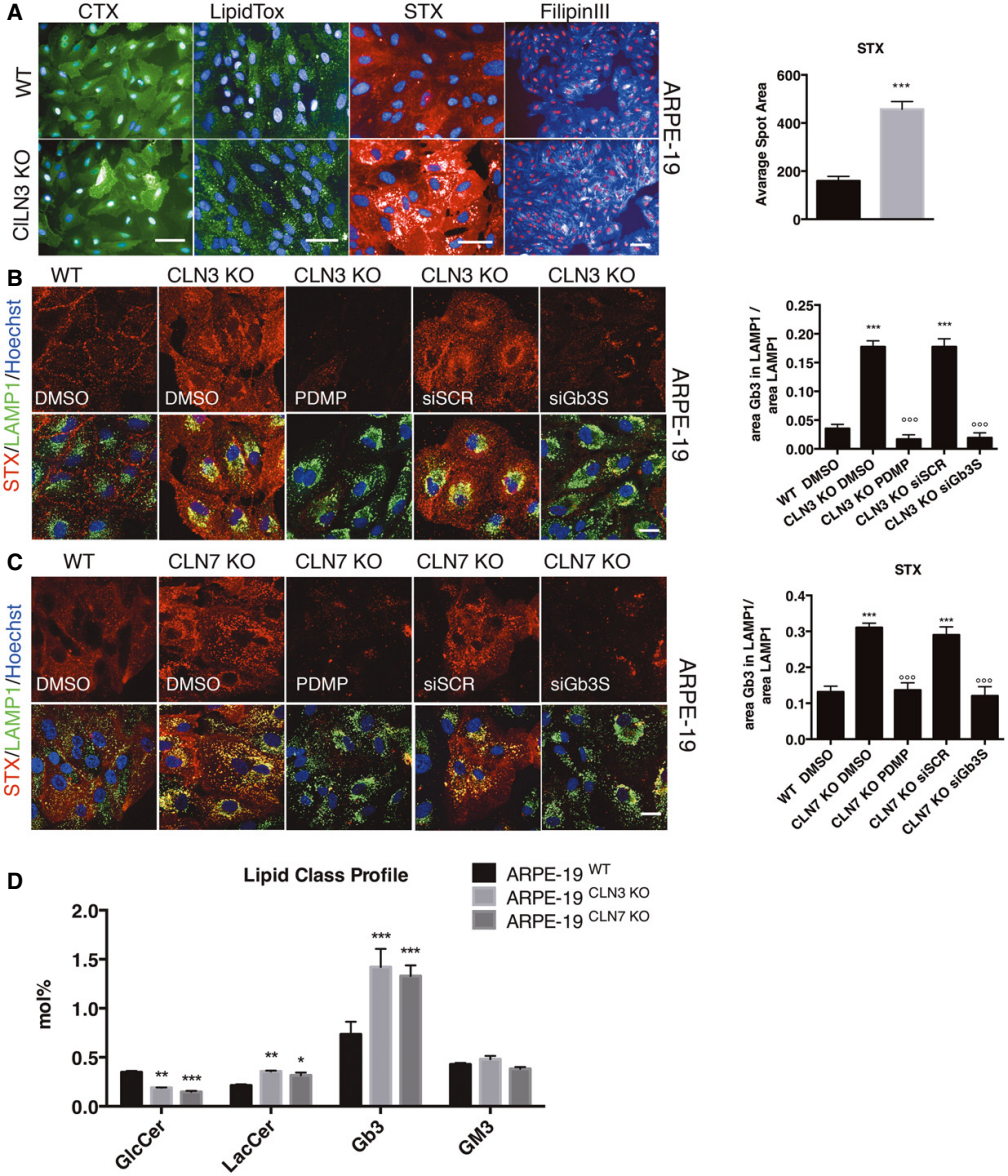
Accumulation of Gb3 in batten disease cellular models ARepresentative Opera images of WT and CLN3 KO ARPE 19 cell lines stained using fluorescent‐conjugated cholera toxin (to detect GM1), LipidTox (to detect neutral lipids), fluorescent‐conjugated Shiga toxin and is quantification (to detect Gb3) and Filipin III (to detect cholesterol). Data are presented as mean ± SD ***P ≤ 0.0001, as determined by Student’s *t*‐test (*n* = 3 biological replicas in duplicate). Scale bars: 50 µm.B, CRepresentative confocal images and their quantification of Gb3 accumulation within the lysosome, detected by fluorescent‐conjugated Shiga toxin, from WT, CLN3‐KO, and CLN7‐KO ARPE‐19 cells treated with PDMP or silenced for Gb3 synthase (siGb3S) (*** versus WT, °°° versus DMSO). Data are presented as mean ± SD, °°°/***P ≤ 0.0001, as determined by ANOVA (*n* = 3 biological replicas in duplicate). Scale bars: 20 µm.DLipidomic analysis of ARPE‐19CLN3KO and ARPE‐19CLN7KO. Data are presented as mean ± SD, *P ≤ 0.01, **P ≤ 0.001, ***P ≤ 0.0001, as determined by ANOVA (*n* = 4 technical replicas).

**Figure EV1:**
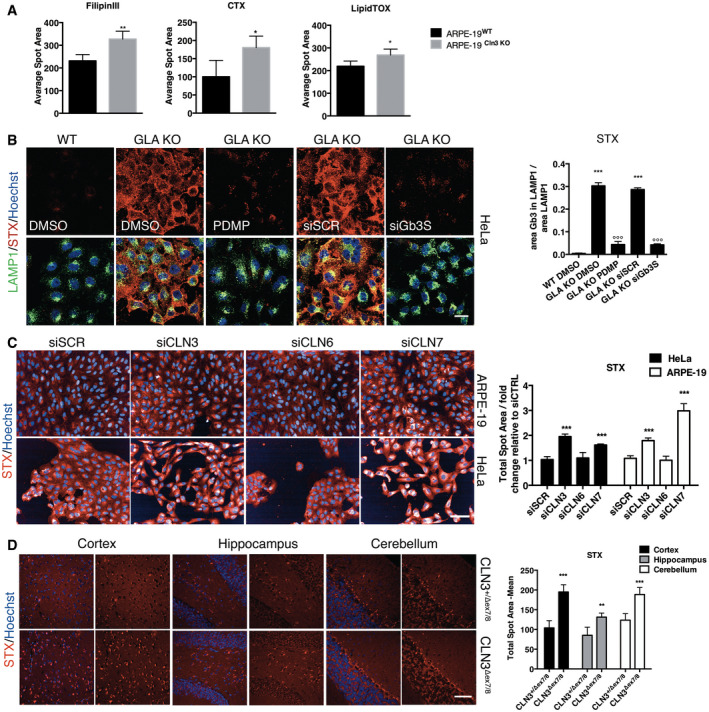
Accumulation of lipids and cell‐based STX assay validation Quantification of WT and CLN3 KO ARPE‐19 cell lines stained using Filipin III (to detect cholesterol) fluorescent‐conjugated cholera toxin (to detect GM1) and LipidTox (to detect neutral lipids). Data are presented as mean ± SD. *P ≤ 0.01, **P ≤ 0.001 as determined by Student’s *t*‐test (*n* = 3 biological replicas in duplicate).Representative confocal images and quantification of Gb3 accumulation within the lysosome detected by Shiga toxin in WT and GLA KO HeLa cells treated with DMSO (controls) or PDMP or silenced for the Gb3 synthase (siGb3S). (***versus WT, °°° versus DMSO) Data are presented as mean ± SD, °°°/***P ≤ 0.0001, as determined by ANOVA (*n* = 3 biological replicas in duplicate) Scale bars: 20 µm.Representative Opera images and their quantification of STX staining in WT ARPE‐19 and HeLa cells silenced for 72 h with siRNAs against a scrambled sequence, CLN3, CLN6, and CLN7. Data are presented as mean ± SD, ***P ≤ 0.0001, as determined by ANOVA (*n* = 3 biological replicas in duplicate) Scale bars: 50 µm.Representative confocal images and quantification of Gb3 accumulation, revealed by STX staining, in brain sections from CLN3^+/Δex7/8^ and CLN3^Δex7/8^ mice at 7.5 months of age. Data are presented as mean ± SD, **P ≤ 0.001, ***P ≤ 0.0001, as determined by ANOVA (*n* = 4 biological replicas) Scale bars: 60 µm. Source data are available online for this figure.

Batten disease (BD) accumulates autofluorescent material, called ceroid lipofuscin, within lysosomes (Mole *et al*, 2005). The nature of this material is heterogeneous (Katz & Robison, 2002; Double *et al*, 2008) and some might derive from oxidation of either modified protein residues or lipids, including triglycerides, free fatty acids, cholesterol, and phospholipids (Double *et al*, 2008). Additionally, various CLN3 disease models present elevation of lipids belonging to the glycosphingolipid pathway such as ceramide, LacCer, GalCer, and gangliosides (Puranam *et al*, 1999; Rusyn *et al*, 2008; Somogyi *et al*, 2018). Based on these observations, we tested different fluorescently‐labelled reporters for the detection of intracellular lipids and sphingosines including; Cholera Toxin B subunit (CTB) to detect GM1 ganglioside (Rusyn *et al*, 2008); LipidTOX for neutral lipids (Somogyi *et al*, 2018); Shiga Toxin subunit B (STX) for globotriaosylceramide (Gb3) (Mallard & Johannes, 2003); and Filipin III to label Cholesterol (Namdar *et al*, 2012). We assessed these reporters in human ARPE‐19 cells depleted for CLN3 (ARPE‐19‐CLN3‐KO) cells by CRISPR genome editing. Although we observed increased levels of various lipid reporters using these cells, the strong elevation of fluorescent‐labelled Shiga Toxin subunit B (STX) staining suggests a dramatic accumulation of globotriaosylceramide (Gb3) in ARPE‐19‐CLN3‐KO cells compared to their WT counterparts (Fig 1A). Also, in comparison with the staining by the other lipid reporters, the STX labelling presents the best signal window for the development of a cell‐based assay in ARPE‐19‐CLN3‐KO cells (Figs 1A and EV1A). To confirm STX staining is selective for Gb3, we used HeLa cells lacking alpha‐galactosidase A (HeLa‐GLA‐KO), a cell model of Anderson‐Fabry disease that results from mutations in the *GLA* gene leading to Gb3 accumulation (Namdar *et al*, 2012) (Fig EV1B). Further, STX staining was negative in cells depleted for Gb3 synthase (Gb3S) or treated with the glucosylceramide synthase inhibitor d‐threo‐1‐phenyl‐2‐decanoylamino‐3‐morpholino‐propanol (PDMP) (Abe *et al*, 2000; Raa *et al*, 2009) in both HeLa‐GLA‐KO and ARPE‐19‐CLN3‐KO cells (Figs 1B and EV1B). Co‐staining with the lysosomal marker LAMP1 indicates that in addition to the general elevation of Gb3 in cellular membranes, most intracellular Gb3 in these knockout cells accumulates within the lysosomal compartment (Fig 1B).

We investigated whether the accumulation of Gb3 is a unique phenotypic feature of CLN3 disease or whether is present in other BDs. Thus, we performed STX staining in cells depleted of either *CLN6* or *CLN7* genes by acute silencing using specific siRNAs in two cell lines, HeLa and ARPE‐19 cells (Fig EV1C). As expected, STX staining revealed that the silencing of CLN3 induces Gb3 accumulation in both HeLa and ARPE‐19 cells (Fig EV1C). Interestingly, the depletion of CLN7 also induces a significant elevation of Gb3 (Fig EV1C). However, Gb3 does not accumulate in cells depleted of CLN6 (Fig EV1C). PCR analysis of the *Gb3S*, *CLN3*, *CLN6,* and *CLN7* mRNA levels confirms the efficiency of gene depletion by siRNAs (Appendix Figs S1 and S2). The selectivity of STX towards Gb3 was again confirmed in ARPE‐19‐CLN7‐KO cells (Fig 1C). Thus, either genetic or pharmacological targeting of Gb3 synthase significantly lowers STX/Gb3 staining in ARPE‐19‐CLN3‐KO, ARPE‐19‐CLN7‐KO, and GLA‐KO cells (Figs 1B and C, and EV1B). Moreover, the direct measurement of Gb3 content by lipidomics analysis of both ARPE‐19‐CLN3‐KO and ARPE‐19‐CLN7‐KO cells shows a doubling of the total Gb3 present in these subtypes of BD (Fig 1D).

**Figure 2:**
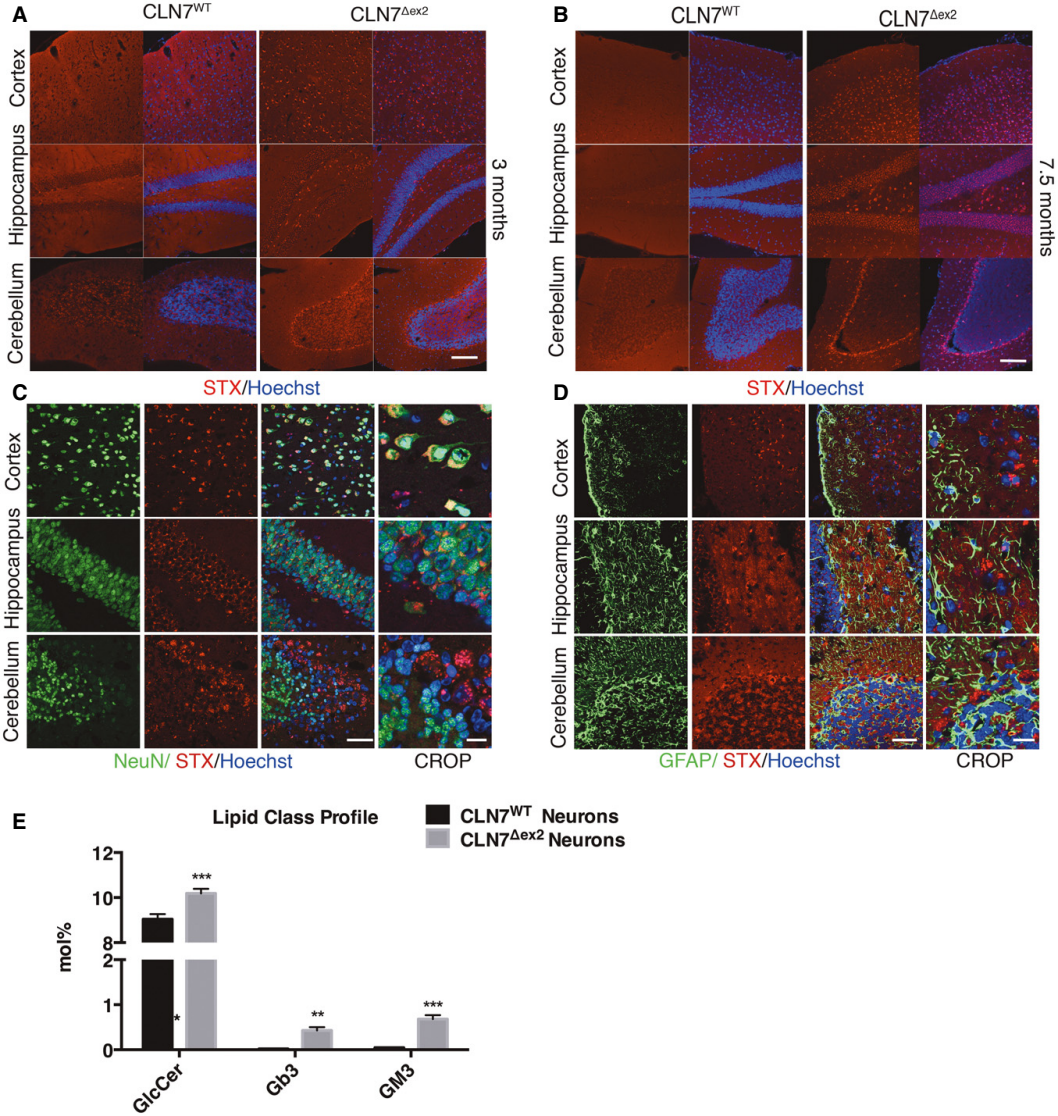
Neural accumulation of Gb3 in brain areas of the CLN7^Δex2^ mouse A, BRepresentative confocal images of Gb3 accumulation, revealed by STX staining, in brain sections from CLN7^Δex2^ mice at 3 and 7.5 months of age compared with CLN7 WT mice. Scale bars: 80 µm.C, DGb3, NeuN and GFAP distribution in brain areas of CLN7^Δex2^ mice at 7.5 months of age. Scale bars: 40 µm, 20 µm.ELipidomic analysis of neurons isolated from CLN7 WT and CLN7^Δex2^ mouse forebrain. Data are presented as mean ± SD, **P ≤ 0.001, ***P ≤ 0.0001, as determined by ANOVA (*n* = 3 biological replicas).

Next, we investigated whether the accumulation of Gb3 observed upon CLN gene depletion *in vitro* also occurs *in vivo*. Thus, we analysed tissue samples derived from the CLN7^Δex2^ mouse, an animal model that well recapitulates the neurological phenotype observed in the human disease (Brandenstein *et al*, 2016). By using the STX labelling of Gb3, we stained brain sections from mice at 3 and 7.5 months of age, which represent the early and late stages of disease progression (Fig 2A and B). We observed a significant accumulation of Gb3 in different brain areas such as the cortex, hippocampus, and cerebellum compared with their corresponding healthy siblings (Fig 2A and B). The abnormal storage of Gb3 appears to be an early pathologic hallmark in the CLN7^Δex2^ disease since it was already present at 3 months of age (Fig 2A). Also, co‐staining with neuronal nuclear antigen NeuN, but not with the astrocyte marker GFAP, indicates that Gb3 mostly accumulates in neurons and not in astrocytes (Fig 2C and D). In line with these observations, the Lipidomics analysis of immuno isolated neurons from fresh CLN7Δex2 mouse forebrain revealed a dramatic neuronal accumulation of both Gb3 and GM3, by 20‐ and 15‐fold, respectively (Fig 2E). As expected, we also observe a similar accumulation of Gb3 in the same regions of the brain from a CLN3 mouse model at 7.5 months (Cotman *et al*, 2002; Staropoli *et al*, 2012), confirming that Gb3 storage is a signature of both BD variants (Fig EV1D).

**Figure 3:**
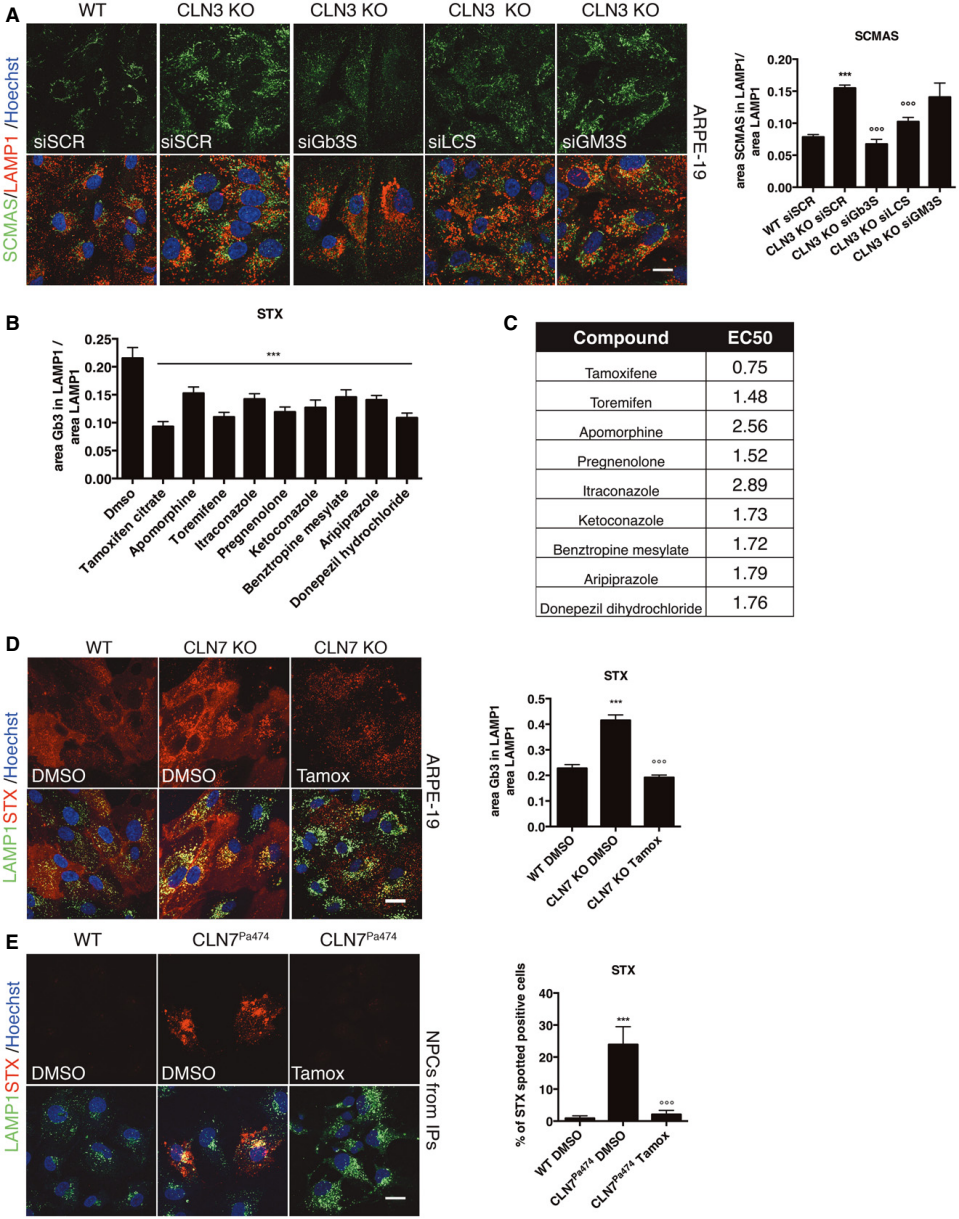
Role of Gb3 in Batten disease and identification of correctors of Gb3 accumulation Representative confocal images and quantification of SCMAS staining within the lysosome of ARPE‐19 WT and CLN3 KO upon depletion of Gb3S (siGb3S), LacCer (siLCS) and GM3s (siGM3S). (***versus WT, °°° versus CLN3 KO siSCR). Data are presented as mean ± SD, °°°/***P ≤ 0.0001, as determined by ANOVA (*n* = 3 biological replicas in duplicate). Scale bars: 20 µm.Identification of FDA compounds reducing Gb3 accumulation: Plot showing the ability of compound hits to reduce Gb3 within the lysosomal compartment compared with DMSO‐treated mutant cells. Data are presented as mean ± SD, ***P ≤ 0.0001, as determined by ANOVA (*n* = 3 biological replicas in duplicate).Table showing the EC50s (Half maximal effective concentration) from the dose–response curves of compound hits. EC50s were calculated using the Prism software.Representative confocal images and quantification of STX staining within the lysosome of ARPE‐19 WT and CLN7 KO in DMSO or treated 48 h with Tamoxifen. (***versus WT, °°° versus CLN3 KO DMSO). Data are presented as mean ± SD, °°°/***P ≤ 0.0001, as determined by ANOVA (*n* = 3 biological replicas in duplicate). Scale bars: 20 µm.Representative confocal images and quantification of STX staining within the lysosome of NPCs WT and derived from CLN7^Pa474^ patient IPSCs in DMSO or treated with Tamoxifen for 48 h. (***versus WT, °°° versus CLN7^Pa474^ DMSO). Data are presented as mean ± SD, °°°/***P ≤ 0.0001, as determined by ANOVA (*n* = 3 biological replicas in duplicate). Scale bars: 20 µm.

The accumulation of Gb3 or GM3 could be a collateral effect arising from the progressive lysosomal dysfunction in the BD models tested, or in contrast, play a direct role in the pathological mechanisms of these diseases. Thus, we silenced Gb3 synthase (siGb3S) to reduce Gb3 generation using specific siRNAs and measured the accumulation of SCMAS, a characteristic component of the pathological storage of CLN3 and CLN7 disease (Palmer, 2015). We observed that acute depletion of Gb3S causes a decrease of SCMAS within the lysosomes of CLN3‐depleted cells (Fig 3A). A similar result was obtained by silencing a more upstream enzyme in the glycosphingolipid synthesis pathway, LacCer synthase (siLCS), whereas silencing of the unrelated GM3 synthase (siGM3S) did not clear the accumulation of SCMAS (Fig 3A). PCR analysis of the Gb3S, LCS, and GM3S mRNA levels confirms the efficiency of the depletion of these genes by siRNAs (Appendix Fig S3). Together, these results indicate that the specific accumulation of Gb3 and not GM3 might play a role in the pathogenesis of two subtypes of Batten disease, and therefore, its targeting might ameliorate the BD phenotype.

### Identification of small molecules reducing Gb3 accumulation in a cell model of Batten disease

**Figure EV2:**
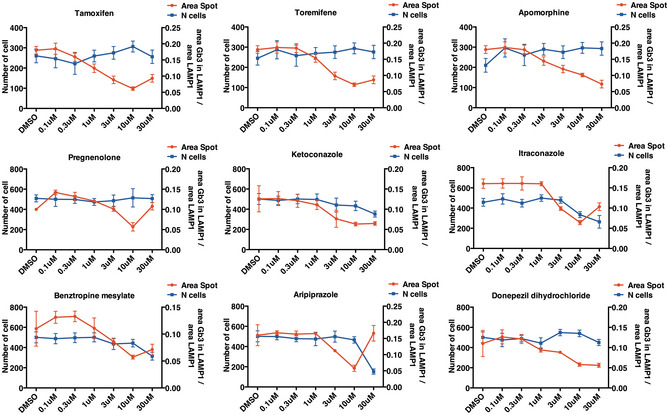
Dose response Dose–response analysis of compound hits derived from the FDA‐screening to identify correctors of Gb3 accumulation. Data from both Gb3 accumulation and nuclei count are presented as mean ± SD (*n* = 4 technical replicas). Source data are available online for this figure.

As we found that abnormal accumulation of Gb3 appears to be pathologic in both *in vitro* and *in vivo* models of CLN3 and CLN7 diseases, we used the STX assay to identify FDA‐approved compounds able to reduce the lysosomal accumulation of Gb3 by quantifying its co‐localization with the lysosomal membrane protein LAMP1 in ARPE‐19‐CLN3‐KO cells (see methods and Appendix Fig S4). The screening of 1,280 FDA drugs resulted in the identification of 9 compound hits. These include two compounds belonging to the stilbenoid class of drugs that are selective oestrogen receptor modulators (tamoxifen and toremifene), one alkaloid (apomorphine), three phenylpiperazines (itraconazole, ketoconazole, and aripiprazole), a derivative of cholesterol (pregnenolone), a diphenylmethane (benztropine) and an acetylcholinesterase inhibitor (donepezil dihydrochloride) (Fig 3B). All nine compounds were further confirmed and tested in the same STX assay in a dose–response format to determine EC50 and cell viability (Figs 3C and EV2). Tamoxifen treatment resulted in the most potent reduction of lysosomal STX accumulation with an EC50 of 0,75 µM without compromising vitality (Fig 3C). We only observed a very weak reduction in the number of nuclei at the highest concentration of tamoxifen that may suggest potential cytotoxicity at doses of > 30 µM (Fig EV2).

**Figure EV3:**
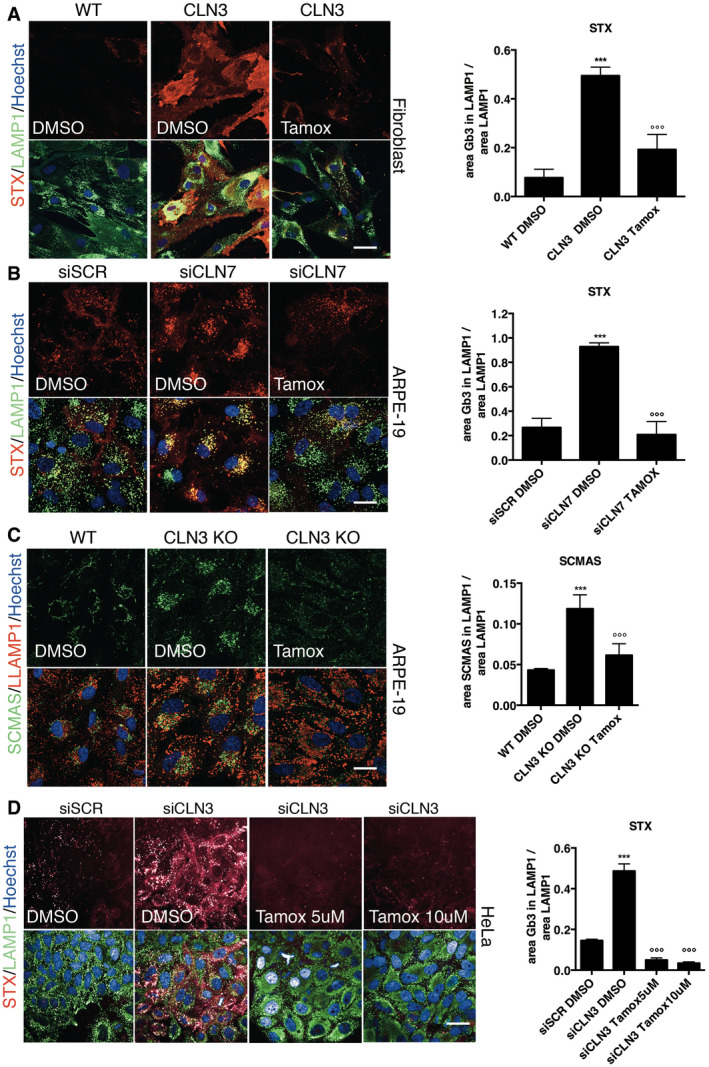
Tamoxifen induces clearance in Batten disease models Representative confocal images and quantification of STX staining within the lysosome of human WT fibroblasts and CLN3 mutants in DMSO or treated with Tamoxifen. (***versus WT, °°° versus DMSO) Data are presented as mean ± SD, °°°/***P ≤ 0.0001, as determined by ANOVA (*n* = 3 biological replicas in duplicate) Scale bars: 20 µm.Representative confocal images and quantification of STX within the lysosome in ARPE‐19 cells silenced with siRNA scramble or against CLN7, in DMSO or treated with Tamoxifen. (***versus siSCR, °°° versus DMSO) Data are presented as mean ± SD, °°°/***P ≤ 0.0001, as determined by ANOVA (*n* = 3 biological replicas in duplicate) Scale bars: 20 µm.Representative confocal images and quantification of SCMAS accumulation within the lysosomes in wild type and ARPE‐19 CLN3 KO cells in DMSO, or treated with Tamoxifen. (***versus WT, °°° versus DMSO) Data are presented as mean ± SD, °°°/***P ≤ 0.0001, as determined by ANOVA (*n* = 3 biological replicas in duplicate) Scale bars: 20 µm.Representative confocal images and quantification of STX within the lysosome. HeLa cells after acute silencing of CLN3 (siCLN3) in DMSO or treated 48 h with Tamoxifen. (***versus siSCR, °°° versus DMSO). Data are presented as mean ± SD, °°°/***P ≤ 0.0001, as determined by ANOVA (*n* = 3 biological replicas in duplicate) Scale bars: 20 µm. Source data are available online for this figure.

Tamoxifen is a readily available EMA‐ and FDA‐approved drug used for several decades for treating breast cancer and other hormone‐related disorders. Importantly, it is also safe in paediatric conditions (Gayi *et al*, 2018). Given the well‐established and widespread prescription of this drug, we decided to focus on tamoxifen for further studies. We confirmed that 10µM tamoxifen promotes the clearance of lysosomal Gb3, stained with STX, in human CLN3 patient fibroblasts (Fig EV3A), ARPE‐19 cells depleted of CLN7 by siRNAs (Fig EV3B), ARPE‐19 CLN7‐KO cells (Fig 3D), and Nestin‐positive neuronal precursor cells (NPCs) derived from CLN7 patient iPSCs (Fig 3E, Appendix Fig S5). Tamoxifen was also able to reduce SCMAS levels in the same cells (Fig EV3C).

**Figure 4:**
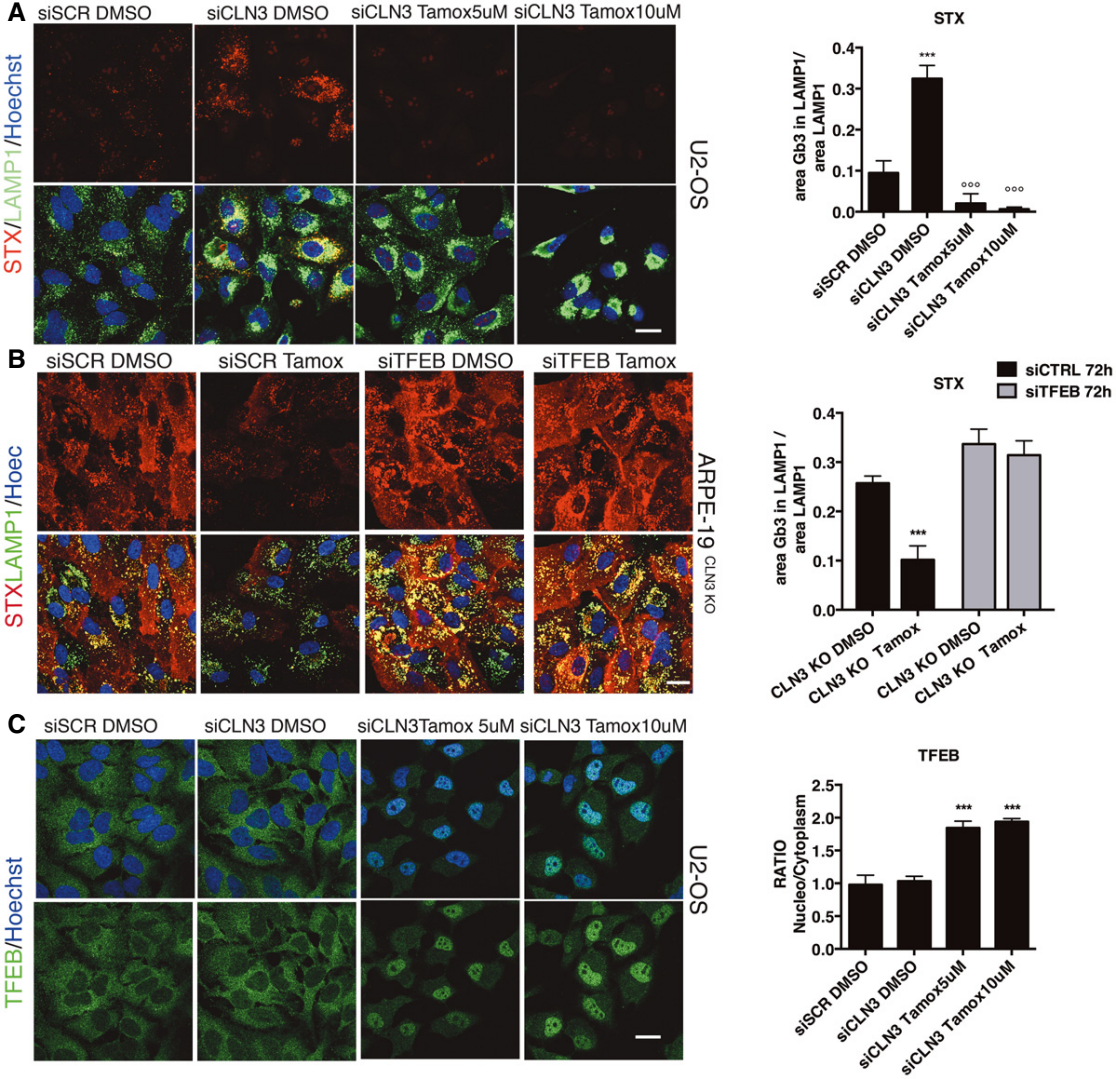
Tamoxifen‐mediated clearance of Gb3 is ER‐independent but TFEB‐dependent Representative confocal images and Quantification of STX within the lysosome in U2‐OS and HeLa cells after acute silencing of CLN3 (siCLN3) in DMSO or treated 48 h with Tamoxifen. (***versus siSCR DMSO, °°° versus siCLN3 DMSO). Data are presented as mean ± SD, °°°/***P ≤ 0.0001, as determined by ANOVA (*n* = 3 biological replicas in duplicate). Scale bars: 20 µm.Representative confocal image and quantification of Gb3 in ARPE‐19 CLN3 KO cells silenced with siRNA against scramble sequence and TFEB (siTFEB) for 72 h and treated for the last 48 h with DMSO or Tamoxifen. Data are presented as mean ± SD, ***P ≤ 0.0001, as determined by ANOVA (*n* = 3 biological replicas in duplicate). Scale bars: 20 µm.Representative confocal image and quantification of TFEB in U2‐OS cells silenced with siRNA against scramble sequence and CLN3 (siCLN3) for 72 h and treated for the last 48 h with DMSO or Tamoxifen (5 and 10 µM). Data are presented as mean ± SD, ***P ≤ 0.0001, as determined by ANOVA (*n* = 3 biological replicas in duplicate). Scale bars: 20 µm.

Tamoxifen is a selective oestrogen receptor modulator (SERM) and the most commonly used drug for the treatment of oestrogen receptor (ER)‐positive breast cancer (Shagufta & Ahmad, 2018). Thus, we asked whether tamoxifen's ability to reduce the accumulation of Gb3 could be through targeting ERs. Surprisingly, at two concentrations, tamoxifen was able to promote Gb3 clearance in two cell lines silenced for CLN3 that do not express ERs, U2OS and HeLa cells (Kallio *et al*, 2008; Selyunin *et al*, 2019) (Figs 4A and EV3D, Appendix Fig S6A and B). These results indicate that tamoxifen induces Gb3 clearance in BD cellular models by a mechanism that is independent of the modulation of ERs. Together, we have developed a novel phenotypic screening tool for repurposing compounds able to reduce lysosomal Gb3 accumulation in BD cells and identified tamoxifen as a potential corrector of the most common subtypes of BD, CLN3 and CLN7 diseases.

### Tamoxifen induces Gb3 clearance through activation of the transcription factor TFEB

**Figure EV4:**
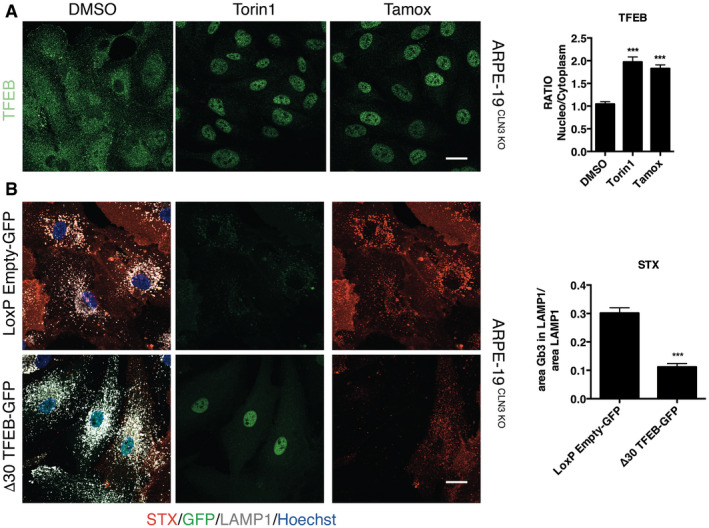
Tamoxifen induces TFEB activation Representative confocal image and quantification of TFEB localization in ARPE‐19 CLN3 KO treated for 3 h with DMSO, Torin1 or Tamoxifen. Data are presented as mean ± SD, ***P ≤ 0.0001, as determined by ANOVA (*n* = 3 biological replicas in duplicate). Scale bars: 20 µm.Representative confocal image and quantification of STX in ARPE‐19 CLN3 KO infected with an inducible vector expressing a nuclear‐localized mutant form of TFEB. Data are presented as mean ± SD, ***P ≤ 0.0001, as determined by ANOVA (*n* = 3 biological replicas in duplicate). Scale bars: 20 µm. Source data are available online for this figure.

The clearance activity of tamoxifen in two different types of BD through a mechanism that is ER‐independent might be explained by the activation of the transcription factor TFEB. This is a master gene of lysosomal function that, upon activation, induces lysosomal clearance of pathological storage in various LSDs, including CLN3 disease (Medina *et al*, 2011). We found that tamoxifen was able to significantly induce TFEB nuclear translocation in ARPE‐19‐CLN3‐KO cells (Fig EV4A). To determine whether TFEB activation is a requirement for tamoxifen‐mediated clearance of Gb3, we tested tamoxifen in ARPE‐19‐CLN3‐KO cells depleted of TFEB by using siRNAs (Fig 4B). While tamoxifen was effective in reducing Gb3 in ARPE‐19‐CLN3‐KO cells treated with scrambled siRNAs (Fig 4B), it was not active in the TFEB‐silenced CLN3‐KO cells (Fig 4B). PCR analysis of the TFEB mRNA levels and Western blotting confirmed the efficiency of siRNA‐mediated depletion of TFEB (Appendix Fig S7A and B). Consistently, viral‐mediated transduction of an inducible vector expressing a nuclear‐localized mutant form of TFEB was sufficient to clear Gb3 in ARPE‐19‐CLN3 KO cells (Fig EV4B). Indeed, tamoxifen was able to induce TFEB nuclear translocation in U2‐OS cells that do not express ERs (Fig 4C), indicating that, such as tamoxifen‐mediated induction of Gb3 clearance, TFEB nuclear translocation is also ER‐independent. mRNA expression analysis by qPCR shows the efficiency of the CLN3 gene depletion in U2‐OS cells (Appendix Fig S6).

**Figure EV5:**
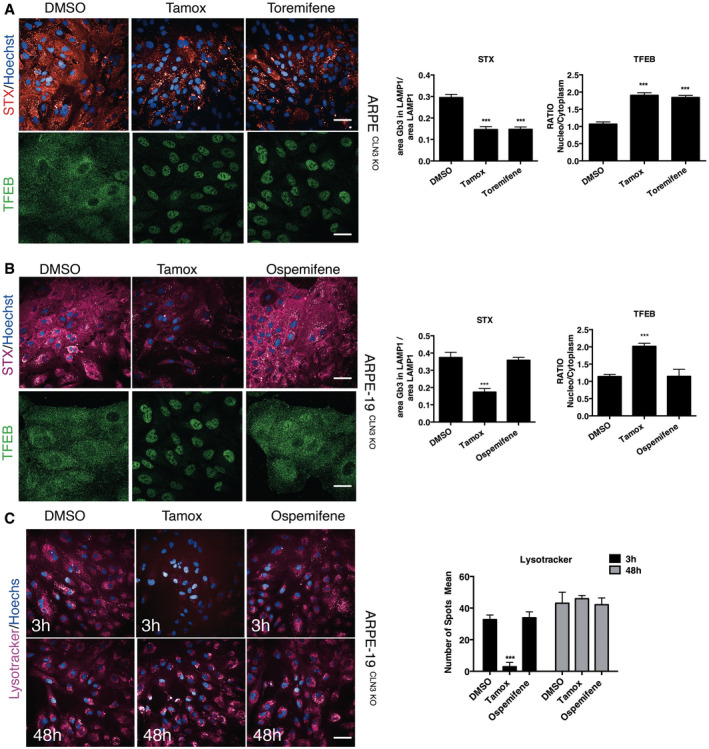
Effects of ER modulators on Gb3 clearance and TFEB activation Representative Opera and confocal images and quantification of TFEB subcellular localization, and STX accumulation within the lysosome of ARPE‐19 CLN3 KO cells treated with DMSO, Tamoxifene or Toremifene for 48 h. Data are presented as mean ± SD, ***P ≤ 0.0001, as determined by ANOVA (*n* = 3 biological replicas in duplicate). Scale bars: 50 µm top and 20 µm down.Representative Opera images and quantification of STX and TFEB localization in ARPE‐19 CLN3 KO in DMSO, Tamoxifen or Ospemifene after 48 h. Data are presented as mean ± SD, ***P ≤ 0.0001, as determined by ANOVA (*n* = 3 biological replicas in duplicate). Scale bars: 50 µm top and 20 µm down.Representative Opera images and quantification of Lysotracker‐Red staining in ARPE‐19 CLN3 KO cells cultivated for 3 and 48 h in the absence (DMSO) or presence of Tamoxifen (Tamox) or Ospemifene. Data are presented as mean ± SD, ***P ≤ 0.0001, as determined by ANOVA (*n* = 3 biological replicas in duplicate). Scale bars: 20 µm. Source data are available online for this figure.

Lysosomotropic compounds possess weak‐base properties that favour their accumulation in lysosomes by ion‐trapping mechanisms (Ohkuma & Poole, 1981; Pisonero‐Vaquero & Medina, 2017). Recent work shows that lysosomotropic anti‐cancer drugs promote lysosome‐mediated cancer drug resistance by stimulating activation of TFEB and the consequent increase in lysosomal biogenesis, lysosomal exocytosis, and autophagy (Zhitomirsky *et al*, 2018). Indeed, two compound hits in our screen, tamoxifen, and toremifene, both present a tertiary amine that makes them weak‐bases able to transiently modify endolysosomal pH by a mechanism that is independent of the ER (Altan *et al*, 1999; Lu *et al*, 2017; Selyunin *et al*, 2019). Consistently, tamoxifen and toremifene were able to reduce Gb3 accumulation and induce TFEB nuclear translocation (Fig EV5A). Conversely, ospemifene, which is structurally related to tamoxifen (Taras *et al*, 2001), possesses similar potency targeting ER‐mediated pathways (Wurz *et al*, 2005), but has a hydroxyl group in place of the tertiary amine of tamoxifen in its side chain, was not able to induce TFEB nuclear translocation or Gb3 clearance at 10 µM (Fig EV5B). These observations suggest that the effects of tamoxifen on inducing TFEB activation and reducing intracellular Gb3 storage in BD models are due to its weak‐base property. Indeed, while 10 uM tamoxifen was able to transiently alkalinize the lysosome after 3 h treatment, measured by a reduction in lysotracker staining, ospemifene was not effective at a similar concentration (Fig EV5C). Also, we confirm previous data showing that the effect of tamoxifen on lysosomal alkalinization is reversible (Fig EV5C) (Actis *et al*, 2021).

**Figure 5:**
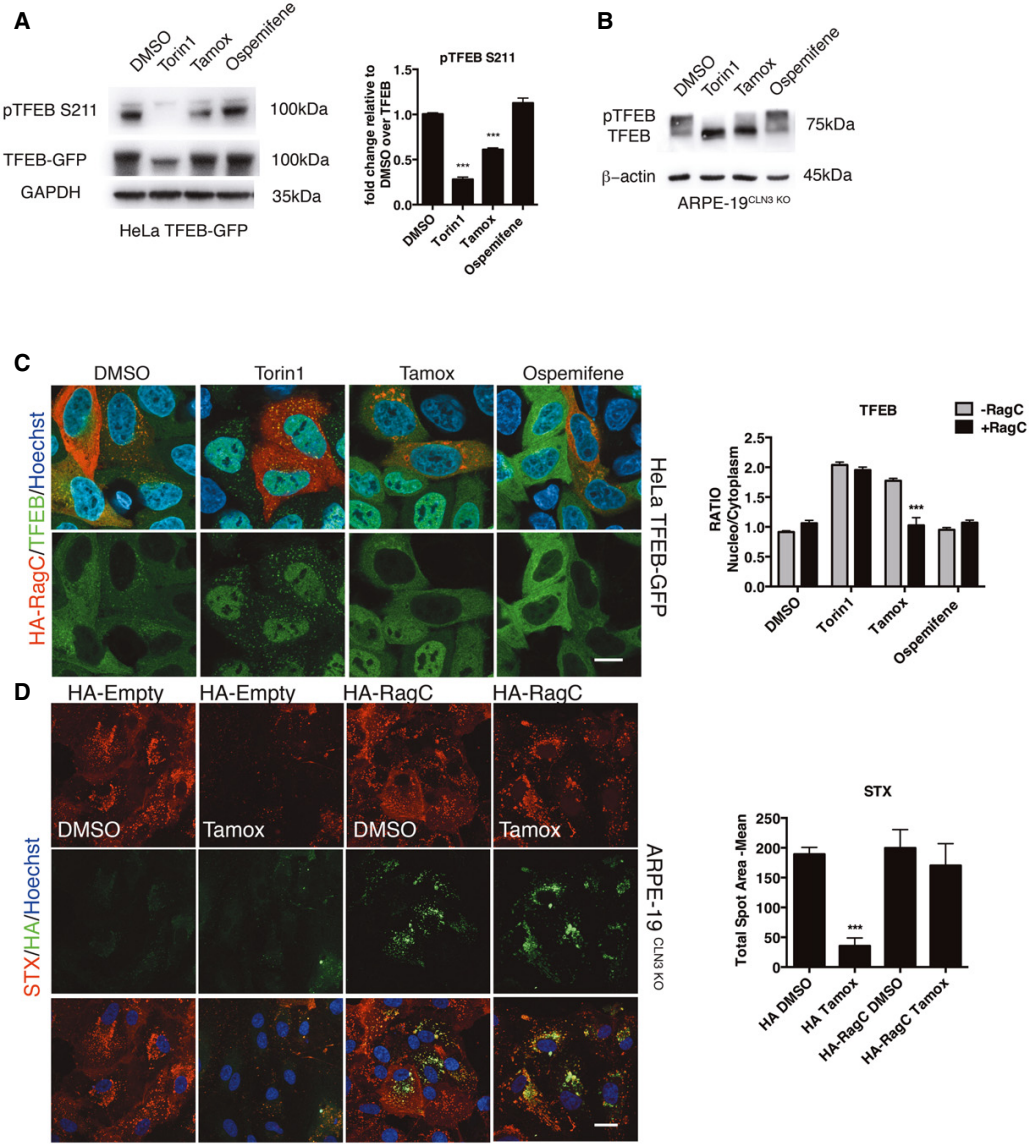
Tamoxifen dephosphorylates TFEB Immunoblot analysis and quantification of pTFEB S211 in HeLa cells stably expressing TFEB‐GFP. Data are presented as mean ± SD, ***P ≤ 0.0001, as determined by ANOVA (*n* = 3 biological replicas). GAPDH immunoblotting was performed as a loading control.Immunoblot analysis of TFEB shift in ARPE‐19 CLN3 KO cells. β‐actin immunoblotting were performed as a loading control.Representative confocal image and quantification of TFEB localization in HeLa TFEB‐GFP transfected with RagC for 48 h and treated for the last 3 h with DMSO, Torin1, Tamoxifen or Ospemifene. Ratios of nuclear to cytosolic TFEB localization in RagC non‐expressing (RagC‐) and RagC‐expressing cells (RagC+) are presented as mean ± SD, ***P ≤ 0.0001, as determined by ANOVA (*n* = 3 biological replicas in duplicate). Scale bars: 20 µm.Representative onfocal images and quantification of STX in ARPE‐19 CLN3 KO cells transfected with empty vector or HA‐RagC for 48 h and treated for 48 h with DMSO or Tamoxifen. STX average spot area presented as mean ± SD, ***P ≤ 0.0001, as determined by ANOVA (*n* = 3 biological replicas in duplicate). Scale bars: 20 µm.

mTOR kinase is the major kinase involved in the negative regulation of TFEB (Laplante & Sabatini, 2012). Thus, we asked whether tamoxifen induces TFEB nuclear translocation by inhibiting mTORC1 activity. Interestingly, mTOR kinase activity measured by both high content imaging assays and immunoblot of its classical substrates, such as p70S6K, 4EPB and ULK1, was not affected by tamoxifen treatment (Appendix Fig S8A and B). However, and in agreement with the induction of TFEB nuclear translocation, tamoxifen reduced the phosphorylation of TFEB while ospemifene did not (Fig 5A and B). Recent work has shown that unlike other substrates of mTORC1, such as those tested above, TFEB is strictly dependent on the activation of RagC and RagD GTPases (Napolitano *et al*, 2020). Indeed, the overexpression of a constitutive active form of RagC (HA‐GST‐RagCS75L) was able to block tamoxifen‐mediated TFEB nuclear translocation and the clearance of Gb3 (Fig 5C and D). As a negative control, we treated HelaTFEB‐GFP cells with the selective mTOR kinase inhibitor torin1 that translocates TFEB even in the presence of active RagC (Napolitano *et al*, 2020) (Fig 5C). As expected, ospemifene was not effective at inducing TFEB translocation and was not affected by RagC expression (Fig 5C). Together, our results indicate that the lysosomotropic feature of tamoxifen induces TFEB nuclear translocation through the inhibition of mTORC1 via a Rag‐dependent mechanism.

### Tamoxifen ameliorates pathologic hallmarks of the CLN7^∆ex2^ mouse model

**Figure 6:**
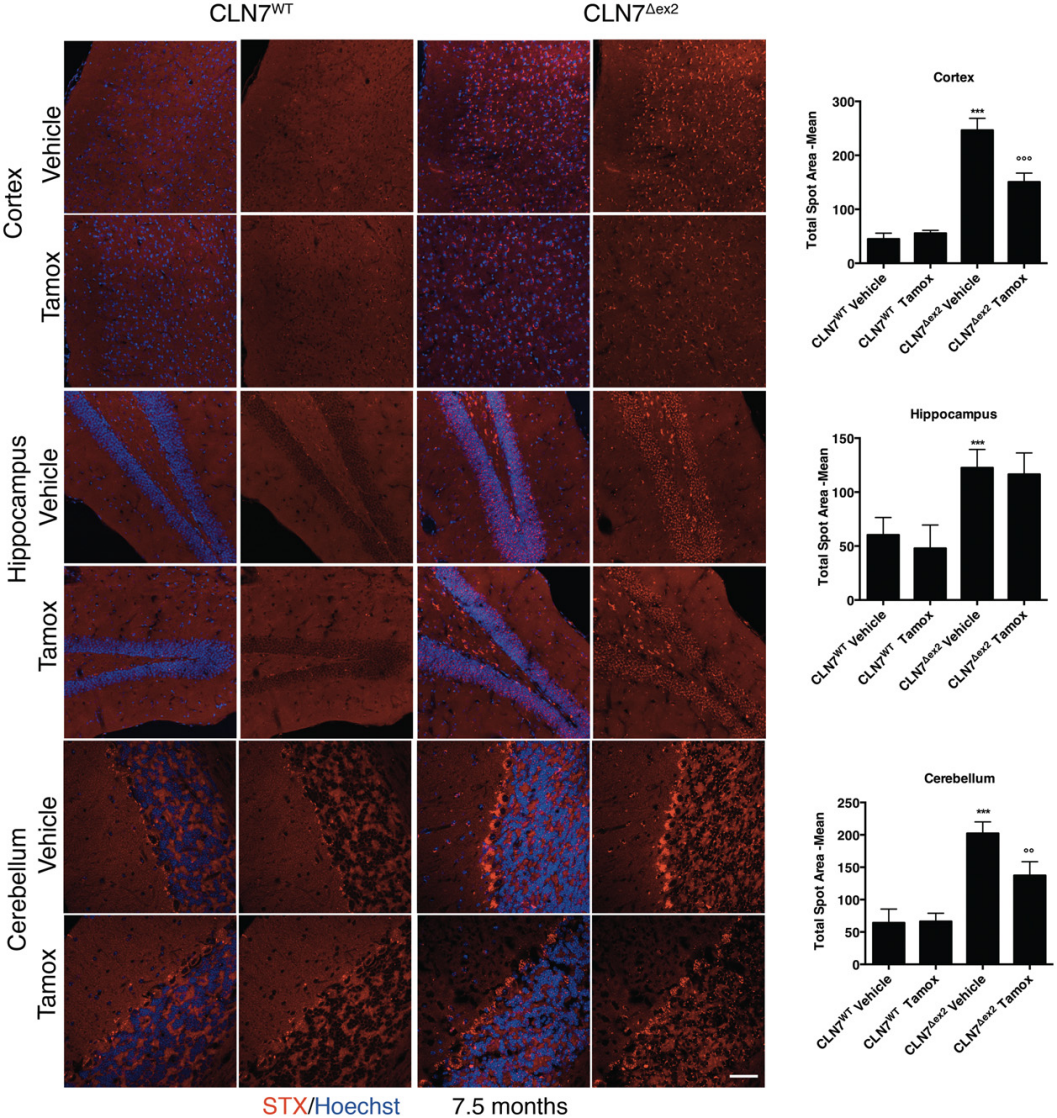
Tamoxifen reduce Gb3 accumulation in brain of CLN7^Δex2^ mice Representative confocal images of STX in the Cortex, Hippocampus and Cerebellum brain section derived from 7.5‐month‐old mouse WT or CLN7^Δex2^ injected with vehicle or Tamoxifen (Tamox). Quantification of confocal images, the plot shows the quantification of the STX average spot area normalized for the number of Hoechst‐positive cells. (***versus WT, °°°/°° versus CLN7^Δex2^ Vehicle). Data are presented as mean ± SD, **/^oo^ *P* ≤ 0.001, ***/^ooo^:*P* ≤ 0.0001, as determined by ANOVA (*N* ≥ 3 biological replicas). Scale bars: 60 µm.

**Figure 7:**
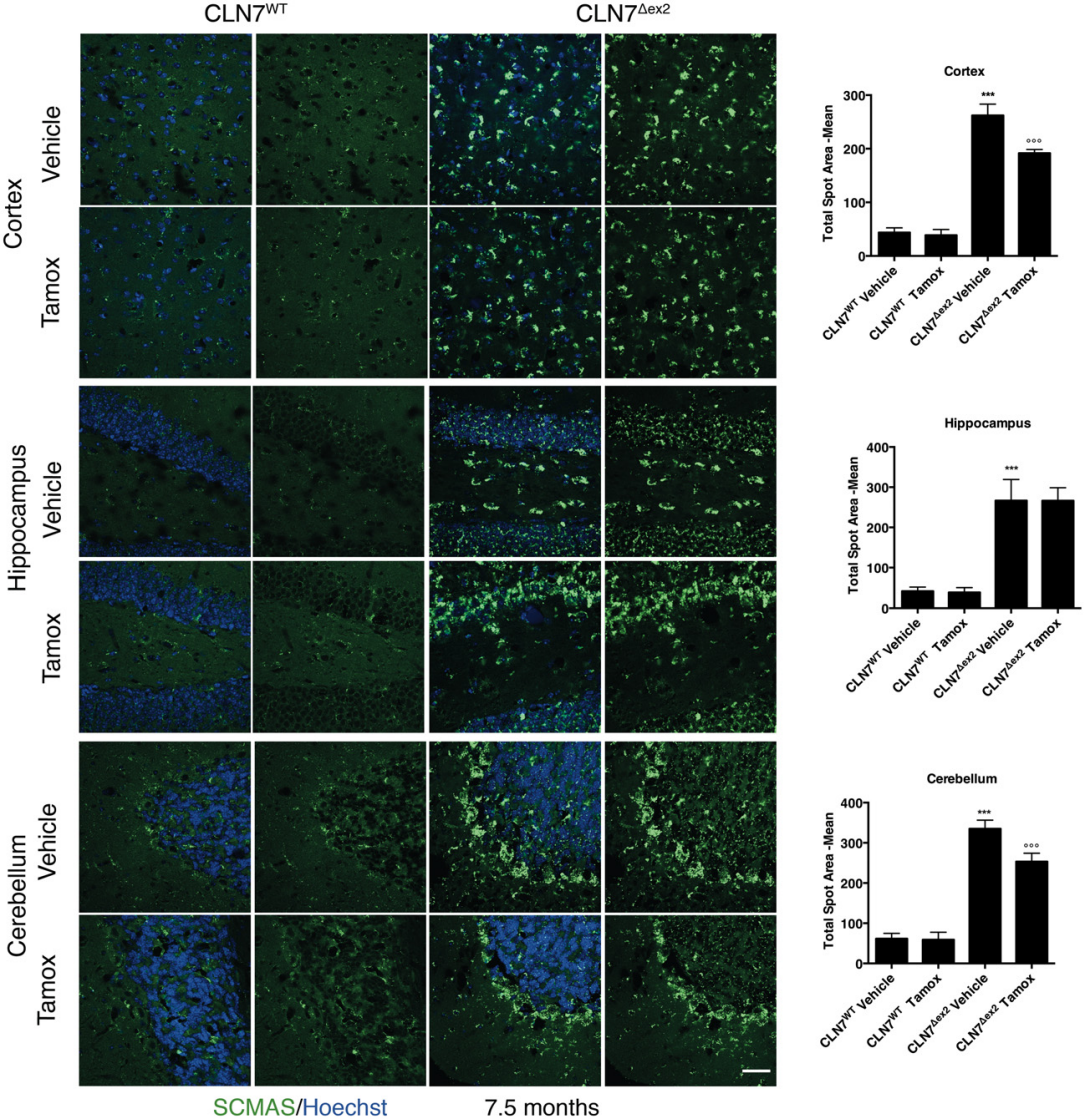
Tamoxifen reduces SCMAS accumulation in the brains of CLN7^Δex2^ mice Representative confocal images of SCMAS in the Cortex, Hippocampus and Cerebellum brain section derived from mouse WT or CLN7^Δex2^ injected with vehicle or Tamoxifen. Quantification of confocal images, the plot shows the quantification of the SCMAS average spot area normalized for the number of Hoechst‐positive cells. (***versus WT, °°° versus CLN7^Δex2^ Vehicle). Data are presented as mean ± SD, ***P ≤ 0.0001, as determined by ANOVA (*N* ≥ 3 biological). Scale bars: 60 µm.

**Figure 8:**
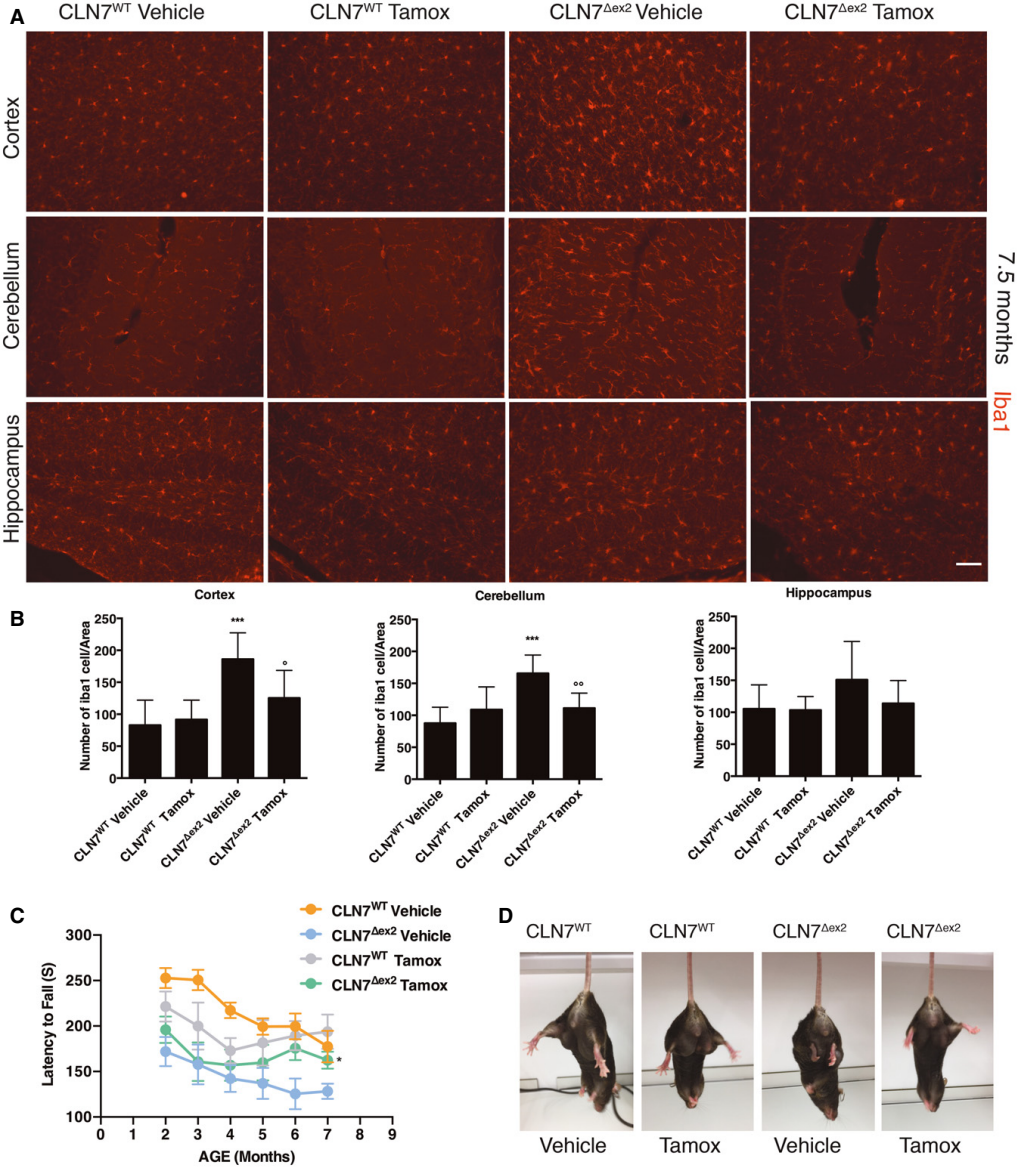
Tamoxifen ameliorates CLN7^Δex2^ phenotype A, BRepresentative confocal images and quantification of IBA‐1 in the Cortex, Hippocampus and Cerebellum brain section derived from WT or CLN7^Δex2^ mice injected with the vehicle or Tamoxifen. (***/**/*versus WT, °°°/°°/° versus CLN7^Δex2^ Vehicle). Data are presented as mean ± SD, *^/o^ *P* ≤ 0.01, **^/oo^ *P* ≤ 0.001, ***P ≤ 0.0001, as determined by ANOVA (*N* ≥ 3 biological replicas). Scale bars: 50 µm.CPlots show the quantification latency to fall from the rotarod. Data are presented as mean ± SD, *P ≤ 0.01, as determined by ANOVA (*N* ≥ 3 biological replicas).DRepresentative images of hindlimb clasping test in mouse WT or CLN7Δex2 injected with vehicle or Tamoxifen.

To test the efficacy of tamoxifen *in vivo*, we selected the CLN7^Δex2^ mouse model (Brandenstein *et al*, 2016), which has a more severe phenotype than the existing CLN3 mouse models (Huber *et al*, 2020) and recapitulates the phenotype of human CLN7 patients. Thus, CLN7^Δex2^ mice show the accumulation of autofluorescent material and SCMAS in the central nervous system, as well as brain gliosis, clasping, hind limb paralysis and seizures (Damme *et al*, 2014; Brandenstein *et al*, 2016; Huber *et al*, 2020). We first tested the ability of tamoxifen to reduce pathologic hallmarks of disease by intraperitoneal injections of tamoxifen (40 mg/kg, twice per week) starting from 2.5 month‐old mice to 7.5 months of age when the disease phenotype is well established (Brandenstein *et al*, 2016). We investigated the ability of tamoxifen to reduce Gb3 storage by using Shiga toxin staining assay. We found a significant reduction of Gb3 in the cortex and the cerebellum, but not the hippocampus, of 7.5‐month‐old tamoxifen‐treated CLN7^Δex2^ mice compared with their age‐matched untreated CLN7^Δex2^ mice (Fig 6). Then, we investigated whether other features of the disease, such as the accumulation of SCMAS and the activation of microglia, could be reversed by the treatment with tamoxifen. Similar to the reduction of Gb3, we found that tamoxifen treatment was able to significantly reduce the accumulation of SCMAS in the cortex and the cerebellum, but not the hippocampus (Fig 7). To test neuroinflammation, we first confirmed that the levels of the small calcium‐binding protein IBA1, a specific marker of both resting and activated populations of microglia, were upregulated in brain sections from the CLN7^Δex2^ mouse (Fig 8A and B) compared to the age‐matched wild‐type mice. By contrast, tamoxifen‐treated CLN7^Δex2^ mice presented a significant reduction of IBA1‐positive cells in both the cortex and the cerebellum (Fig 8A and B). We found a similar trend in the hippocampus, although it was not statistically significant (Fig 8A and B). Together, these results strongly indicate that tamoxifen treatment can reduce the pathological storage of GB3 and SCMAS as well as reduce signs of neuroinflammation in the brain of CLN7^Δex2^ mice.

Motor deficits are one of the primary clinical features of BD (Raininko *et al*, 1990; Kovács *et al*, 2006; Mole *et al*, 2019). By 8 months of age, Cln7^Δex2^ mice begin to manifest signs of neurological deterioration attested by clasping phenotype, hind‐leg paralysis, tremor and myoclonus epilepsies (Brandenstein *et al*, 2016). Motor deficits and balance are detectable by measuring the latency to fall from the rotarod and can be used as a read‐out of the efficacy of potential therapeutic compounds in BD models (Finn *et al*, 2011). We performed the rotarod test (Finn *et al*, 2011) during the whole period of treatment (6 measurements in total). Interestingly, tamoxifen‐treated wild‐type mice improved with age. CLN7^Δex2^ mice exhibit a marked locomotor dysfunction in the late stages of the disease, while tamoxifen‐treated CLN7^Δex2^ mice were less likely to fall when compared with the untreated CLN7^Δex2^ mice, although did not improve to the extent of wild‐type mice (Fig 8C). We tested motor dysfunction by using the hindlimb clasping test (Lieu *et al*, 2013). In healthy mice, both hindlimbs remain splayed outward away from the abdomen with splayed toes. Partial retraction of one or both hindlimbs towards the body indicates a moderate phenotype. Severe motor dysfunction correlates with both hindlimbs partially retracted towards the body and touching the abdomen. As expected, the wild‐type animals showed a normal extension reflex in the hindlimbs, while 7.5‐month‐old mutant mice did not. We found that hindlimb clasping improved in the tamoxifen‐treated CLN7^Δex2^ mice (Fig 8D). Together, the results of the motor tests suggest a partial recovery of motor coordination capacity in CLN7^Δex2^ mice treated with tamoxifen. In conclusion, *in vivo* administration of tamoxifen improves biochemical markers and motor deficits of CLN7 disease.

## Discussion

We have discovered using cellular models of CLN3 and CLN7 diseases, and NPCs generated from iPS cells derived from CLN7 patient fibroblasts that Gb3 accumulated within lysosomes as a consequence of disease. This Gb3 accumulation is even more striking in neurons of CLN3 and CLN7 mouse models. *In vitro*, silencing of Gb3 synthase leads to the reduction of Gb3 levels, and also decreases the characteristic disease storage of subunit SCMAS, indicating that the altered levels of Gb3 might be part of the neuropathological features characterizing these diseases. Previous alterations of some glycosphingolipids such as ceramide, LacCer and GM3 have been described in BD models (Puranam *et al*, 1999; Rusyn *et al*, 2008; Schmidtke *et al*, 2019). Indeed, in addition to endogenous Gb3 accumulation, the lipidomic analysis of CLN3 and CLN7 KO models showed increased levels of LacCer, the common precursor of both Gb3 and GM3 synthesis. Lipidomics analysis of freshly isolated neurons from the forebrain of CLN7 mice, displays an even higher elevation of Gb3 and GM3 and their precursor GlcCer. However, we observed that while the depletion of LacCer synthase or Gb3 synthase reduced SCMAS accumulation, the silencing of the unrelated GM3 synthase did not, indicating that unbalancing the pathway involving the synthesis/degradation of Gb3 may contribute to the pathogenesis of CLN3 and CLN7 diseases. Recent SILAC‐based quantitative analysis of the lysosomal proteome of MEFs from CLN7 mice showed significant differences in the expression of proteins involved in lipid trafficking and glycosphingolipid catabolism (Danyukova *et al*, 2018). Thus, these changes may be related to the pathologic accumulation of Gb3 in CLN7 disease. The same study revealed that the CLN5 protein is also downregulated in CLN7 MEFs (Danyukova *et al*, 2018). CLN5 mutations present with a similar disease onset, progression and phenotypes as CLN7 disease suggesting that both genes may act in a common pathway that is disturbed in both diseases. Additionally, CLN5 can interact with CLN3 (Vesa *et al*, 2002), suggesting that CLN5 disease, together with CLN3 and CLN7, may belong to a subset of BDs accumulating Gb3. Future studies are needed to confirm this hypothesis and to determine the interaction of these three proteins in the regulation of brain Gb3 levels.

Our observations allowed us to develop a cell‐based HCI for Gb3 accumulation assay to screen > 1,200 FDA compounds. Among the compound hits, we focused on the selective oestrogen receptor modulator (SERM) tamoxifen that promotes the clearance of lysosomal Gb3 in CLN3 and CLN7 cells. We found that tamoxifen activity is independent of its ER modulation but requires TFEB activation. TFEB can promote clearance of pathological storage both *in vitro* and *in vivo* in various models of LSDs (Sardiello *et al*, 2009; Spampanato *et al*, 2013; Palmer, 2015; Kauss *et al*, 2020). Indeed, we confirmed that TFEB expression was sufficient to promote Gb3 clearance in ARPE‐19‐CLN3‐KO cells. Thus, tamoxifen‐mediated clearance via TFEB activation may represent a small molecule‐based strategy to treat common types of BD. Using ospemifene, an analog of tamoxifen that does not contain the tertiary amine conferring lysosomotropic properties, we determined that the activation of TFEB requires the weak‐base nature of tamoxifen. Consistently, another lysosomotropic analog of tamoxifen, toremifene, also induces Gb3 clearance and TFEB activation *in vitro*. Another lysosomotropic SERM, raloxifene (Selyunin *et al*, 2019), is effective in neuroprotection and immunomodulatory effects in a mouse model of Parkinson’s disease (Poirier *et al*, 2016), supporting the potential benefits of repurposing approved stilbenoids to treat LSDs and more common neurodegenerative disorders. Since most of the approved CNS‐penetrant drugs are lysosomotropic, future studies are needed to elucidate whether all compounds with this feature can promote clearance of pathological storage through the activation of TFEB or other properties are involved. Also, the logP and pKa properties of five out of the nine hits identified correspond to drugs with potential lysosomotropic properties (logP > 2; pKa 6‐11), supporting further studies of these compounds in BD models.

Mechanistically, we observed that tamoxifen induced TFEB nuclear translocation by specifically impairing mTORC1‐mediated phosphorylation of TFEB without affecting mTORC1 activity towards canonical substrates such as S6K, 4EBP and ULK1. Giving the recent observations that RagC/D GTPase activity can mediate selective phosphorylation of mTORC1 substrates (Napolitano *et al*, 2020), we postulated that lysosomotropic properties of tamoxifen specifically affect RagC/D activity leading to the dephosphorylation of TFEB. A few reports suggest that tamoxifen can alter glycosphingolipid metabolism in cancer cells (Lavie *et al*, 1997; Morad & Cabot, 2015). Thus, future studies are needed to determine whether the reported effects of tamoxifen on glycosphingolipid regulation may contribute to Gb3 clearance and whether the activation of TFEB is involved.

We tested the efficacy of tamoxifen as a therapeutic agent by treating CLN7^Δex2 mice^. Tamoxifen treatment using a therapeutic concentration of 40 mg/kg ameliorated various phenotypic hallmarks of CLN7 mouse including; (i) the accumulation of Gb3, (ii) SCMAS, (iii) microglia activation and (iv) improved motor dysfunction measured by rotarod and hindlimb clasping.

In humans, tamoxifen is used orally and crosses the blood–brain barrier. It has shown neuroprotective activity in rat and dog models of brain ischaemia and stroke, respectively (Kimelberg *et al*, 2003; Kimelberg, 2008; Boulos *et al*, 2011), and it has been used in the treatment of a variety of childhood disorders (Maddalozzo *et al*, 1993; Walter *et al*, 2000; Derman *et al*, 2003; Lawrence *et al*, 2004; Kreher *et al*, 2005). Adverse effects in these populations have been rare, and tamoxifen seems to have an excellent safety profile overall. Together with our data, therefore, we propose tamoxifen as a novel therapeutic for two types of BD, CLN3 and CLN7 diseases.

## Materials and Methods

### Cell culture and siRNA transfection

ARPE‐19 (retinal pigment epithelium (RPE) cell line), U2‐OS and HeLa cells were purchased at ATCC and cultured in DMEM F12 and DMEM, supplemented with 10% foetal bovine serum, 200 µM l‐glutamine, 100 µM sodium pyruvate, 5% CO_2_ at 37°C. Human ARPE‐19 cells were chosen because they are diploid and non‐transformed. ARPE‐19 depleted of CLN3 was generated by Dr. J. Monfregola at TIGEM (Naples) and was cultured in DMEM F12 supplemented with 10% foetal bovine serum, 200 µM l‐glutamine, 100 µM sodium pyruvate, 5% CO_2_ at 37°C.

Human control patient fibroblasts were provided by Professor Brunetti (TIGEM), CLN3 patient fibroblasts were purchased from Coriell Institute and cultured in DMEM supplemented with 15% foetal bovine serum, 200 µM l‐glutamine, 5% CO_2_ at 37°C. HeLa TFEB/TFE3 KO cells were generated from Dr. R.J. Youle from the National Institutes of Health, Bethesda. U2OS was purchased at ATCC and cultured in DMEM supplemented with 10% foetal bovine serum, 200 µM l‐glutamine, 5% CO_2_ at 37°C.

Cells were silenced with 25 nM of siRNA against CLN3, CLN6, CLN7, Gb3S and TFEB for 72 h using Lipofectamine RNAimax (Thermo Fisher) according to the protocol from the manufacturer. All control experiments to confirm silencing efficiency (by qPCR or immunoblot) are reported.

### Generation of ARPE‐19 CRISPR/Cas9 CLN3 KO and CLN7 KO cell lines

ARPE‐19 (ATCC CCRL‐2320) cells carrying a homozygous deletion of a C were generate by using the CRISPR/Cas9 system. The gRNA sequence with low off‐target score have been selected using the http://crispor.tefor.net/crispor.py tool. An “ALL in One” vector expressing Cas9, the specific gRNA and GFP was obtained from SIGMA (CAS9GFPP). The CAS9GFPP was nucleofected in ARPE19 cells using the Amaxa nucleofector kit V (Cat No VCA‐1003) and transfected GFP‐positive cells were FACS sorted into 96‐well plates to obtain single‐cell derived colonies carrying the INDEL mutations. Upon genomic DNA extraction and DNA Sanger sequencing, clones carrying the c.1055delA for CLN3 KO cells and c.103delC for CLN7 KO cells were selected and expanded.

### Drugs and cellular treatments

The following drugs were used to perform the assays: Tamoxifen (10 µM, SIGMA 3–48 h), Toremifene (10 µM, SIGMA 48 h) and Ospemifene (10 µM, SIGMA 48 h).

Screening and dose‐response: Cells were plated on 384‐well plates (2 × 10^4^ cells per well). After 24 h, cells were treated with 10 µM compounds or 0.1% dimethyl sulfoxide (DMSO) in complete medium. The Prestwick Library consists of 1,280 FDA‐approved drugs, all off‐patent, dissolved in DMSO. The drugs from the 96‐well source plate were diluted and compacted in 384‐well plates to a concentration of 100 μM in the DMEM medium (working plate). To study the effect of the drugs, 5 μl of the drugs at 100 μM in DMEM medium were added to plates containing 45 μl of medium (10 μM final drug concentration with 0.1% DMSO). As a positive control of Gb3 reduction, we used the glucosylceramide synthase inhibitor PDMP.

For the dose–response confirmation test, compounds were serially diluted from 10 mM stock into complete medium and added to plates starting at 30 to 0.1 µM. The final concentration of DMSO did not exceed 0.3% in the dose–response assays.

Cells were incubated together with drugs 48 h at 37°C and 5% CO_2_.

### Screening quality control analysis

We confirmed the robustness of the STX‐assay using two different quality control scores (*Z*‐score and SSMD‐score) (Appendix Fig S4A). To exclude toxicity in the primary screening, we discard compounds reducing the cell viability to 40% compared to DMSO‐treated controls. To ensure reproducibility, we also analyse the correlation between plate replicates (Appendix Fig S4B). As a cut‐off for hit selection, we select compounds reducing the lysosomal accumulation of Gb3 greater than the mean of Gb3 in DMSO‐treated CLN3 cells minus two standard deviations.

### Antibodies and western blotting

The following antibodies were used: β‐Actin (Santa Cruz sc‐47778, 1:4,000), ULK1 (Cell signaling cat. 8054 1:1,000), Phospho‐ULK1 (Ser757) (Cell signaling cat. 6888 1:1,000), p70 S6 Kinase (Cell signaling cat. 2708 1:1,000), Phospho‐p70 S6 Kinase (Thr389) (Cell signaling cat. 9205 1:1,000), GAPDH (6C5) (Santa Cruz sc‐32233, 1:2,000), 4EBP (Cell signaling cat. 9644 1:1,000), p4EBP (Cell signaling cat. 9456 1:1,000), TFEB (Cell signaling cat. 4240S 1:1,000) and TFEB‐pS211 (custom‐generated in collaboration with Bethyl Laboratories 1:1,000). For immunoblot, the total cell lysates were prepared by solubilization of cell pellets in 10mM Tris–HCl pH 8.0 and 0.2% SDS supplemented with protease and phosphatase inhibitors (SIGMA). Protein concentration was determined by the Bradford method. After SDS‐PAGE and immunoblotting, the proteins recognized by the specific antibody were visualized by chemiluminescence methods (Luminata Crescendo Western HRP substrate, Millipore) using peroxidase‐conjugated anti‐rabbit or anti‐mouse secondary antibodies (Millipore). Membranes were developed using a Chemidoc UVP imaging system (Ultra‐Violet Products Ltd) and densitometric quantification was performed in unsaturated images using ImageJ (NIH).

### Immunofluorescence

For immunofluorescence, the following antibodies were used: LAMP1 (Santa Cruz cat. sc‐20011, 1:400), TFEB (Cell signalling cat. 4240S 1:200), Phospho‐S6 Ribosomal Protein (Ser235/236) (Cell signalling cat. 9865 1:400), anti‐ATP‐synthase C (Abcam ab181243 1:500), HA.11 clone 16B12 (BioLegend 901501 1:500) and Nestin (Thermo Fisher MA1‐110 1:200). Cells were fixed in PFA 4% for 20 min and permeabilized with blocking buffer saponin whereas for TFEB immunostaining cells were permeabilized in 0.1% (w/v) Triton X‐100, 1% (w/v) horse serum, and 1% (w/v) BSA in PBS. Cells were incubated with the indicated primary antibodies for 2 h and subsequently incubated with secondary antibodies for 45 min (AlexaFluor 488 A21202, AlexaFluor 488 A11008, AlexaFluor 568, A10037, AlexaFluor 568 A10042, all Thermo Fisher 1:400). For confocal imaging, the samples were examined under a Zeiss LSM 800 confocal microscope. Optical sections were obtained under a × 63 or ×40 immersion objective at a definition of 1,024 × 1,024 pixels (average of eight or sixteen scans), adjusting the pinhole diameter to 1 Airy unit for each emission channel to have all the intensity values between 1 and 254 (linear range). For high content images, we used the OPERA high content imager from PerkinElmer.

For image analysis, we used Columbus 2.6.0.127073 (built at 03:56 on 05/02/19) released by PerkinElmer. This online platform is based on Harmony High‐Content Imaging and Analysis Software which provides an easy quantification of complex cellular phenotypes.

### Lipidomic analysis

For Lipidomics analysis, 200,000 cell culture lysates or 10 µg of protein of homogenized immunoisolated neuron samples were spiked with 4.28 μl of internal standard lipid mixture containing 500 pmol of Chol‐d6, 100 pmol of Chol‐16:0‐d7, 100 pmol of DAG 17:0‐17:0, 50 pmol of TAG 17:0‐17:0‐17:0, 100 pmol of SM 18:1;2‐12:0, 30 pmol of Cer 18:1;2‐12:0, 30 pmol of GalCer 18:1;2‐12:0, 50 pmol of LacCer 18:1;2‐12:0, 300 pmol of PC 17:0‐17:0, 50 pmol of PE 17:0‐17:0, 30 pmol of PI 16:0‐16:0, 50 pmol of PS 17:0‐17:0, 30 pmol of PG 17:0‐17:0, 30 pmol of PA 17:0‐17:0, 25 pmol of Gb3 18:1;2‐17:0, 25 pmol of GM3 18:1;2‐18:0‐d5, 25 pmol of GM2 18:1;2‐18:0‐d9, 25 pmol of GM1 18:1;2‐18:0‐d5m 25 pmol of GD1a 18:1;2‐17:0 and subjected to lipid extraction at 4°C, as described elsewhere (Sampaio *et al*, 2011). Briefly, the sample was dissolved in 200 μl of 155 mM ammonium bicarbonate and then extracted with 1 ml of chloroform‐methanol (10:1) for 2 h. The lower organic phase was collected, and the aqueous phase was re‐extracted with 1 ml of chloroform‐methanol (2:1) for 1 h. The lower organic phase was collected and evaporated in a SpeedVac vacuum concentrator. Lipid extracts were dissolved in 100 μl of infusion mixture consisting of 7.5 mM ammonium acetate dissolved in propanol:chloroform:methanol [4:1:2 (vol/vol)]. Samples were analysed by direct infusion in a QExactive mass spectrometer (Thermo Fisher Scientific) equipped with a TriVersa NanoMate ion source (Advion Biosciences). 5 µl of sample was infused with gas pressure and voltage set to 1.25 psi and 0.95 kV, respectively.

HexCer was detected in the 10:1 extract, by negative ion mode FTMS as a deprotonated ion by scanning *m/z* = 520–1,050 Da, at *R_m/z_* _= 200_ = 280,000 with lock mass activated at a common background (*m/z* = 529.46262) for 30 s. Every scan is the average of 2 micro‐scans, automatic gain control (AGC) was set to 1E6 and maximum ion injection time (IT) was set to 200 ms. Hex2Cer and Hex3Cer were detected in the 2:1 extract, by positive ion mode FTMS as protonated ions by scanning *m/z* = 800–1,600 Da, at *R_m/z_* _= 200_ = 280,000 with lock mass activated at a common background (*m/z* = 1194.81790) for 30 s. Every scan is the average of 2 micro‐scans, automatic gain control (AGC) was set to 1E6 and maximum ion injection time (IT) was set to 50ms. GM3 was detected in the 2:1 extract, by polarity switch to negative ion mode FTMS as a deprotonated ion by scanning *m/z* = 1,100–1,650 Da, at *R_m/z_* _= 200_ = 280,000 with lock mass activated at a common background (*m/z* = 1175.77680) for 30 s. Every scan is the average of 2 micro‐scans, automatic gain control (AGC) was set to 1E6 and maximum ion injection time (IT) was set to 50 ms. All data were acquired in centroid mode.

All data were analysed with the lipid identification software, LipidXplorer (https://doi.org/10.1186/gb‐2011‐12‐1‐r8). Tolerance for MS and identification was set to 2 ppm. Data post‐processing and normalization to internal standards were done manually. For the sake of simplicity, only the pertinent data are displayed (HexCer, Hex2Cer, Hex3Cer and GM3) and normalized to the total lipid identified. Raw data are represented in Appendix Tables S1–S4.

### Induced pluripotent stem cells (iPSC) and Neural Progenitor Cells (NPC) generation

CLN7 patient fibroblasts were obtained from the UCL NCL repository (https://www.ucl.ac.uk/ncl‐disease/). iPSCs were generated from a cell line originally derived pre‐2005 from skin biopsy fibroblasts from a CLN7 patient (UCL 474 Pa) and then characterized and differentiated to NPC as previously described (FitzPatrick *et al*, 2018). Human iPSC‐derived NPCs from an age‐matched control patient and patient 474 Pa harbouring a homozygous CLN7 mutation (c.1393C > T; p.R465W) were plated on Matrigel. Matrix in Nunc™ Lab‐Tek™ 8‐well Chamber Slides and cultured in Neural Expansion Medium (NEM) with DMEM/F12, NEAA, N‐2 supplement, B‐27 supplement, heparin, bFGF protein and penicillin/streptomycin.

### Ethics approval

Informed consent to generate cell lines used in this research was obtained from all human subjects, and experiments conformed to the WMA Declaration of Helsinki and to the principles set out in the USA Department of Health and Human Services Belmont Report.

### Fluorescent assays

#### Cholera toxin

Cells were cultured on 96‐well plates and incubated in a serum‐free medium containing 1 μg/ml AlexaFluor488‐labelled cholera toxin subunit B (C22841 Thermo Fisher Scientific) for 30 min at 33°C. Subsequently, cells were washed three times with PBS and fixed in 4% (w/v) paraformaldehyde for 10 min at room temperature. Nuclei were stained with Hoechst for 10 min.

#### Filipin III

Cells were fixed with 4% paraformaldehyde 10 min. The paraformaldehyde was rinsed with PBS and quenched with 50 mM glycine in PBS. Cells were then incubated for 2 h at room temperature in PBS containing 50 μg/ml filipin III (from Streptomyces filipinensis SIGMA F4767). Nuclei were stained with DRQ5 1:5,000 (62254 Thermo Fisher Scientific) for 10 min.

#### Shiga Toxin

Cells were fixed with 4% paraformaldehyde 10 min and permeabilized in 0.1% (w/v) saponin, 0.5% (w/v) BSA and 50 mM NH_4_Cl in PBS (blocking buffer saponin). STX were incubated alone or with LAMP1 antibody in blocking buffer saponin for 2 h (1:50,000) and subsequently incubated with secondary antibodies for 45 min. Nuclei were stained with Hoechst for 10 min.

#### Lysotracker

Cells were cultured on 96‐well plates and incubated in a serum‐free medium containing 1:10,000 AlexaFluor568‐labelled Lysotracker Red (L7528 Thermo Fisher Scientific) for 20 min at 33°C. Subsequently, cells were washed three times with PBS and fixed in 4% (w/v) paraformaldehyde for 10 min at room temperature. Nuclei were stained with Hoechst for 10 min.

### RNA extraction and quantitative PCR

Total RNA was extracted from cells using the RNeasy Plus Mini Kit (Qiagen). Reverse transcription was performed using the QuantiTect Rev Transcription Kit (Qiagen). Real‐time quantitative Reverse Transcription PCR (qRT–PCR) was performed using the LightCycler^®^ System 2.0 (Roche Applied Science). HPRT was used for qRT–PCR as a reference gene. The parameters of real‐time qRT–PCR amplification were according to Roche recommendations. Primer sequences are available upon request.

#### Ethical use of animals

Mice were bred at the Animal Experimentation Unit of the University of Salamanca. All protocols were performed according to the European Union Directive 86/609/EEC and Recommendation 2007/526/EC, regarding the protection of animals used for experimental and other scientific purposes, enforced in Spanish legislation under the law 6/2013. All protocols were approved by the Bioethics Committee of the University of Salamanca. The Cln3^Δex7/8^ knock‐in mice were bred in a pathogen‐free animal facility at the University Medical Center Hamburg‐Eppendorf according to institutional guidelines.

### Mice genotyping by a polymerase chain reaction

For Cln7^Δex2^ genotyping, a PCR with the following primers was performed 5′‐TGGTGCATTAATACAGTCCTAGAATCCAGG‐3′, 5′‐CTAGGGAGGTTCAGATAGTAGAACCC‐3′, 5′‐TTCCACCTAGAGAATGGAGCGAGATAG‐3′, resulting in a 290 bp band in the case of Cln7^Δex2^ mice, and 400 bp for wild type (Brandenstein *et al*, 2016). In the case of Cln3^Δex7/8^ knock‐in mice, a band with 250 bp is obtained for wild type, and a 500 bp in the case of Cln3^Δex7/8^ knock‐in mice using the following primers: 5′‐ CAGCATCTCCTCAGGGCTA‐3′, 5′‐CCAACATAGAAAGTAGGGTGTGC‐3′, 5′‐ GAGCTTTGTTCTGGTGCCTTC‐3′, 5′‐ GCAGTCTCTGCCTCGTTTTCT‐3′ *(19)*.

### Neuronal cell isolation from the brain cortex

Adult mouse brain (from 6 months animals) tissue was dissociated with the Adult Brain Isolation Kit (Miltenyi Biotec). Neurons were separated in the dissociated cells, after removal of debris and red blood cells, and neurons were separated with the Neuron Isolation Kit (Miltenyi), according to the manufacturer’s protocol. The identity of the isolated fraction was confirmed previously (Lopez‐Fabuel *et al*, 2016) by Western blot against the neuronal marker microtubule‐associated protein 2 (MAP2).

### Tamoxifen administration

Twice per week 40 mg of tamoxifen per gram of body weight (from a stock solution of tamoxifen in 20% (vol/vol) ethanol and 80% (vol/vol) of sunflower oil) were injected intraperitoneally to male mice of 2.5 months old until they reach 7.5 months old. Body weight was evaluated before injections, as well as the general aspect of the mice (eyes, fur and behaviour) to see the influence of tamoxifen in mice. Control animals received injections with the tamoxifen vehicle (20% (vol/vol) ethanol and 80% (vol/vol) of sunflower oil). A rotarod test was done before treatment beginning, and every month during it, to all the studied animals.

### Locomotor assessment (Rotarod Test)

The rotarod test (Rotarod apparatus, model 47600, Ugo Basile) was used to analyse motor balance and coordination. Male mice were previously trained during three consecutive days, 2 days before the test. The rotarod conditions were gradually accelerated from 4 to 45 r.p.m., reaching the final speed at 270 s. The latency to fall was evaluated and averaged for each animal during the 3 days of the experiment.

#### Mouse perfusion and immunohistochemistry

Male mice were anaesthetized by intraperitoneal injection of a mixture of xylazine hydrochloride (Rompun; Bayer) and ketamine hydrochloride/chlorbutol (Imalgene; Merial) (1:4) at 1 ml per kg body weight and then perfused intra‐aortically with 0.9% NaCl followed by 5 ml per g body weight of Somogyi (Paraformaldehyde, 4% (wt/vol) and picric acid, 0.2% (vol/vol), in 0.1 MPB, pH 7.4. After perfusion, (i) brains were dissected out sagittally in two parts and post‐fixed with Somogyi for 2 h at room temperature; (ii) eyes were extracted, stabbed in the lens, post‐fixed overnight in 4% (wt/vol) of paraformaldehyde, and then were subjected to 5 washes of 10 min in 0.1 M of PB solution, followed by cryoprotection in 15 and 30% of sucrose (wt/vol) in 0.1 MPB sequentially at 4°C. Brain blocks were rinsed successively for 15, 30 min, 1 and 2 h with 0.1 MPB solution and cryoprotected in 10, 20, and 30% (wt/vol) sucrose in PBS sequentially, until they sank. After cryoprotection, 40‐μm thick sagittal sections were obtained with a freezing‐sliding cryostat (Leica; CM1950 AgProtect). The sections were collected serially in a 12‐well plate in 0.1 MPB, rinsed three times for 10 min in 0.1 M PBS and used for subsequent immunohistochemistry and autofluorescence evaluation. The section‐containing wells that were not used were kept in freezer mix (polyethylene glycol, 30% by volume and glycerol 30% by volume in 0.1 MPB) at −20°C. In the case of autofluorescence, sections were mounted with Fluoromount (Sigma‐Aldrich) aqueous mounting medium and lamelles cover‐objects (Thermo Fisher Scientific). For immunohistochemistry, sections were incubated sequentially in (i) 5 mg/ml sodium borohydride in PBS for 30 min (to remove aldehyde autofluorescence); (ii) three PBS washes of 10 min each; (iii) 1:500 anti‐IBA‐1 (019‐19741, Wako), 1:200 anti‐ATP‐synthase C (ab181243 abcam), 1:500 anti‐NeuN (MAB377 Millipore), 1:500 anti‐GFAP (G6171, Sigma) and 1:1,000 STX in 0.02% Triton X‐100 (Sigma‐Aldrich) and 5% goat serum (Jackson Immuno‐Research) in 0.1 MPB for 72 h at 4°C; (iv) three PB washes of 10 min each; (v) fluorophore conjugated secondary antibodies, 1:500 Cy2 goat anti‐mouse or 1:500 Cy3 goat anti‐rabbit (Jackson Immuno‐Research) in PB for 2 h at room temperature; and (vi) 0.5 μg/ml Hoechst in PB for 10 min at room temperature. After being rinsed with PB, sections were mounted with Fluoromount.

### Imaging and quantification

For confocal imaging, the sections were examined under a Zeiss LSM 800 confocal microscope. Optical sections were obtained under a × 63 or ×40 immersion objective at a definition of 1,024 × 1,024 pixels (average of eight or sixteen scans), adjusting the pinhole diameter to 1 Airy unit for each emission channel to have all the intensity values between 1 and 254 (linear range). For image analysis, we used Columbus 2.6.0.127073 (built at 03:56 on 05/02/19) released by PerkinElmer. This online platform is based on Harmony High‐Content Imaging and Analysis Software.

### Statistical analysis

For *in vitro* studies, we perform three or more independent experiments without data exclusion. No formal blinding was performed, but independent researchers were performing similar experiments to confirm the results.

For *in vivo* studies, we used ≥ 3 animals per group. InVivoStat software was used to determine that six animals were enough as the sample size to achieve a 20% of variation with statistical significance with a power of 100%. The presence of signs of behavioural or health problems was the criteria to exclude animals. The adscription of animals to each group was done randomly. Animals were selected arbitrarily to receive tamoxifen or vehicle injections. The analysis of animal samples was blind. The samples were identified by numbers, and the results were grouped after the analysis. For behavioural tests, the animals were tested together. Data have been analysed with the support of the Bioinformatics Core in TIGEM.

Microsoft Excel, GraphPad Prism and R software packages were used to analyse the data. Sample numbers and other information (mean or SD, number of replicates and specific statistical tests) are indicated in the main text or Figure legends.

We have first used the Shapiro–Wilk test to check the normality assumption. The *P*‐value in our data is not significant, and therefore, we can assume normality.

We used Levene’s test to check the homogeneity of variances. The *P*‐value of Levene’s test in our data is not significant. It means that there is no significant difference between variances across groups. Then, one‐way or two‐way ANOVA have been applied for all charts with more than two groups. Student's *t*‐test was used for statistical analysis when comparing only two groups.

## Author contributions

DLM and CS conceived the study and produced the manuscript, which was improved by all authors. CS, IL‐F, MG‐M, JPB, SM, LGW, JM, JLS, AE and MG‐F produced experimental data. JPB, GN, LJ, SS, SEM, TB, MADM, ASR, EKK, DP, TM, CMG and AB contributed to the interpretation of parts of the results.

## Conflict of interest

The authors declare that they have no conflict of interest.

## Supporting information



Appendix

Expanded View Figures PDF

Source Data for Expanded View and Appendix

Source Data for Figure 1

Source Data for Figure 2

Source Data for Figure 3

Source Data for Figure 4

Source Data for Figure 5

Source Data for Figure 6

Source Data for Figure 7

Source Data for Figure 8

## Data Availability

This study includes no data deposited in external repositories.
