# Supplementary material for: Repurposing of tamoxifen ameliorates CLN3 and CLN7 disease phenotype
Source: EMBO Mol Med. 2021 Aug 19;13(10):e13742. doi: 10.15252/emmm.202013742 (PMC8495452; doi:10.15252/emmm.202013742)
Supplement: Supplementary file 1 — Appendix [file EMMM-13-e13742-s003.pdf]

Appendix Table of content

Appendix Figure S1 Quantitative PCR
Appendix Figure S2 Quantitative PCR
Appendix Figure S3 Quantitative PCR
Appendix Figure S4 Quality data determination of the cell-based high content screening
Appendix Figure S5 Nestin in NPCs
Appendix Figure S6 Quantitative PCR
Appendix Figure S7 Quantitative PCR and immunoblot of TFEB expression
Appendix Figure S8 mTORC1 pathway
Appendix Table S1 Lipidomic raw data Species profile
Appendix Table S2 Lipidomic raw data Class Profile
Appendix Table S3 Lipidomic raw data SpeciesProfile
Appendix Table S4 Lipidomic raw data Class Profile

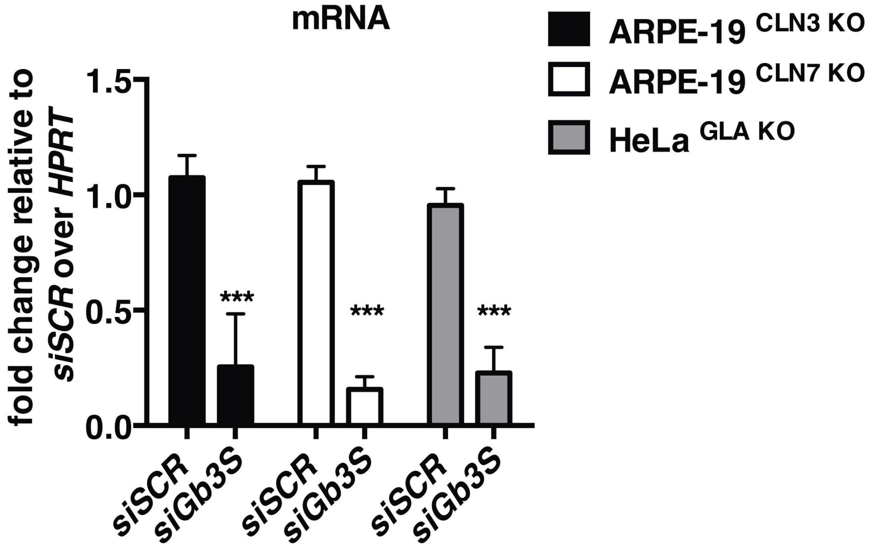

Appendix Figure S1. Image relative to Figure1 and EV1. Quantitative PCR showing mRNA levels of silenced gene *Gb3S* compared to a scramble sequence (*siSCR*). Data are presented as mean ± SD, ***: P ≤ 0.0001, as determined by ANOVA (n=3)

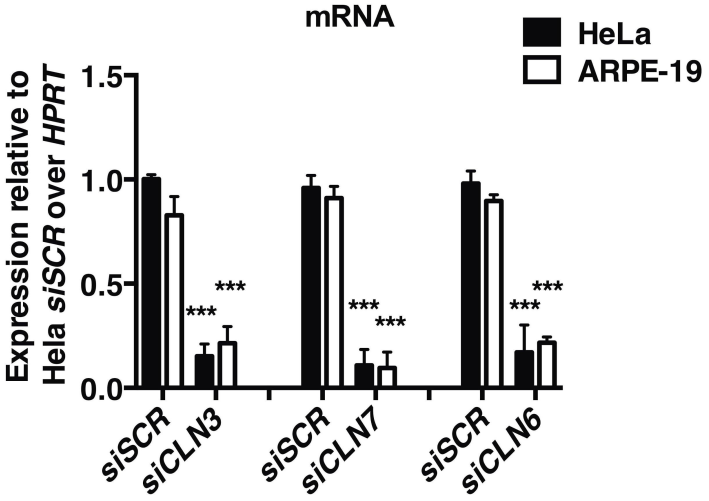

Appendix Figure S2. Image relative to EV1. Quantitative PCR showing mRNA levels of silenced genes *CLN3*, *CLN7* and *CLN6* compared to a scramble sequence (*siSCR*). Data are presented as mean ± SD, ***: P ≤ 0.0001, as determined by (n=3)

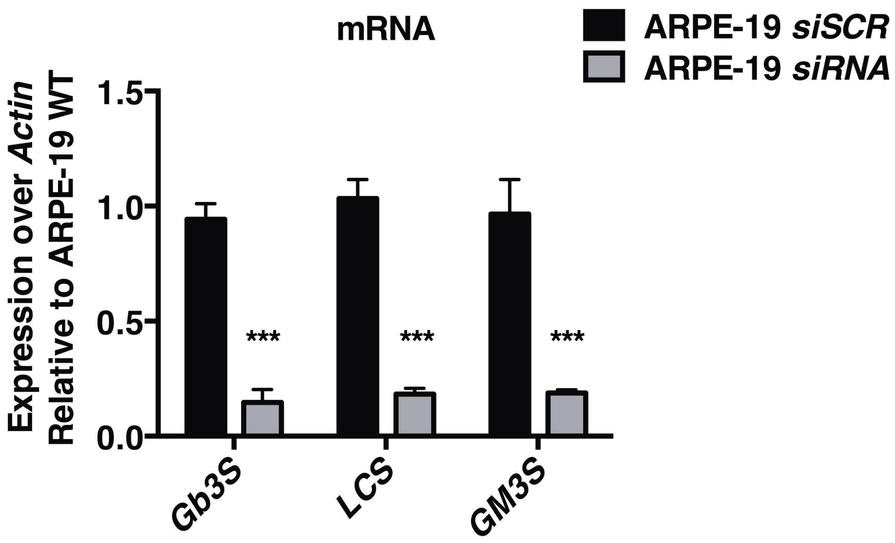

Appendix Figure S3. Image relative to Figure 3. Quantitative PCR showing Gb3 synthase (*Gb3S*), LacCer synthase (*LCS*) and GM3 synthase (*GM3S*) mRNA levels in ARPE-19 CLN3 KO cells transfected with scrambled (*SCR*, black bars) or gene-specific siRNAs (grey bars). Data are presented as mean ± SD, ***: P ≤ 0.0001, as determined by (n=3)

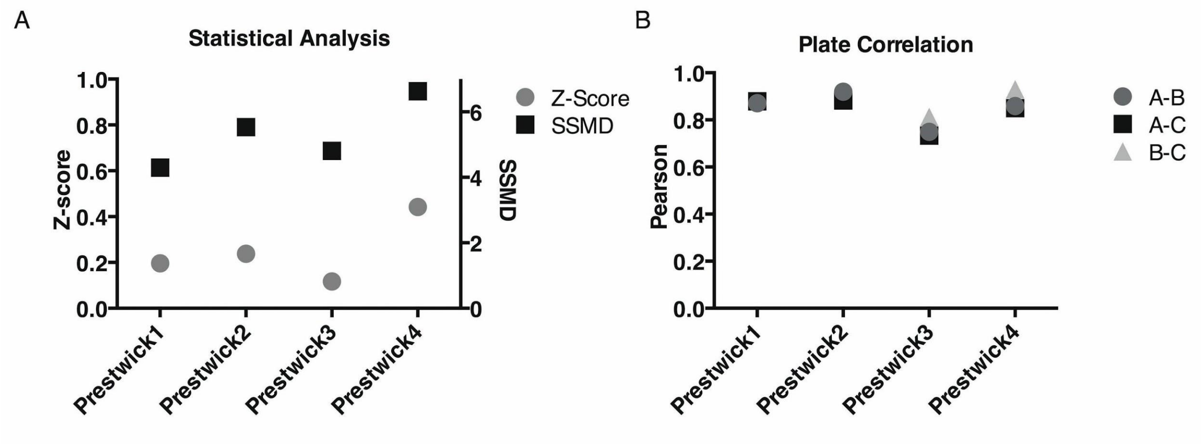

Appendix Figure S4. Image relative to Figure 3. (A) Quality data determination of the cell-based high content screening performed (Z-score and SSMD score values) and (B) correlation between plate replicates of the screening.

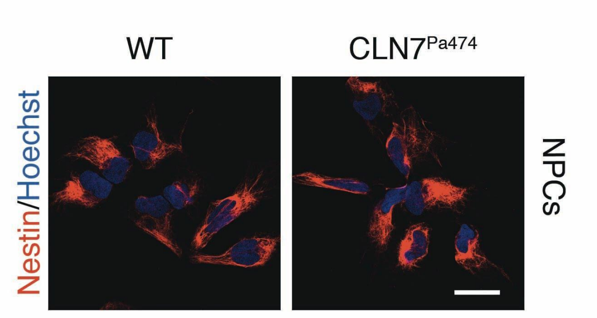

Appendix Figure S5. Image relative to Figure 3. Representative confocal images of Nestin in NPCs WT and derived from a CLN7 patient IPSC. Scale bars: 20 µm.

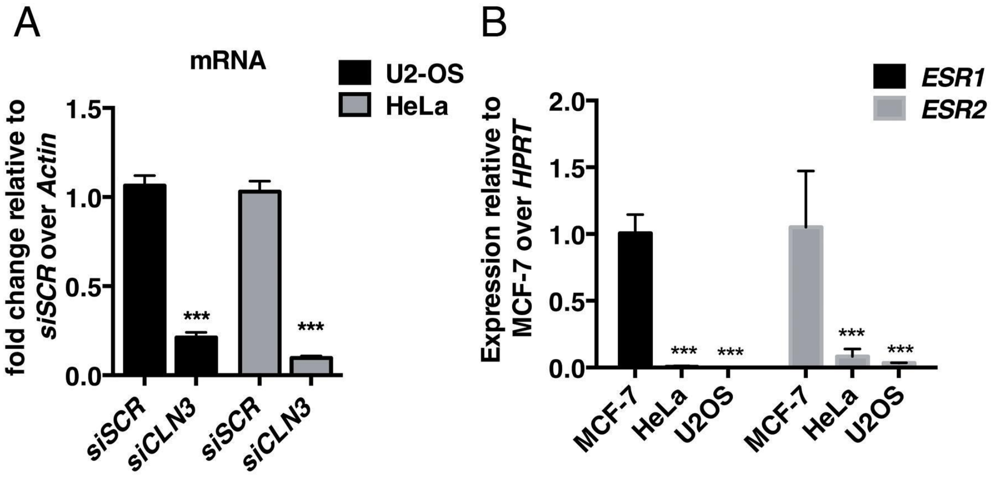

Appendix Figure S6A-B. Image relative to Figure 4 and EV3. A) Quantitative PCR showing mRNA levels of silenced gene *CLN3* **(siCLN3)** compared to a scramble sequence *(siSCR)*. Data are presented as mean ± SD, ***: P ≤ 0.0001, as determined by ANOVA (n=3). B) Quantitative PCR showing mRNA levels of Estrogen receptor 1 and 2 in HeLa and U2OS cells compared to MCF-7. Data are presented as mean ± SD, ***: P ≤ 0.0001, as determined by ANOVA (n=3).

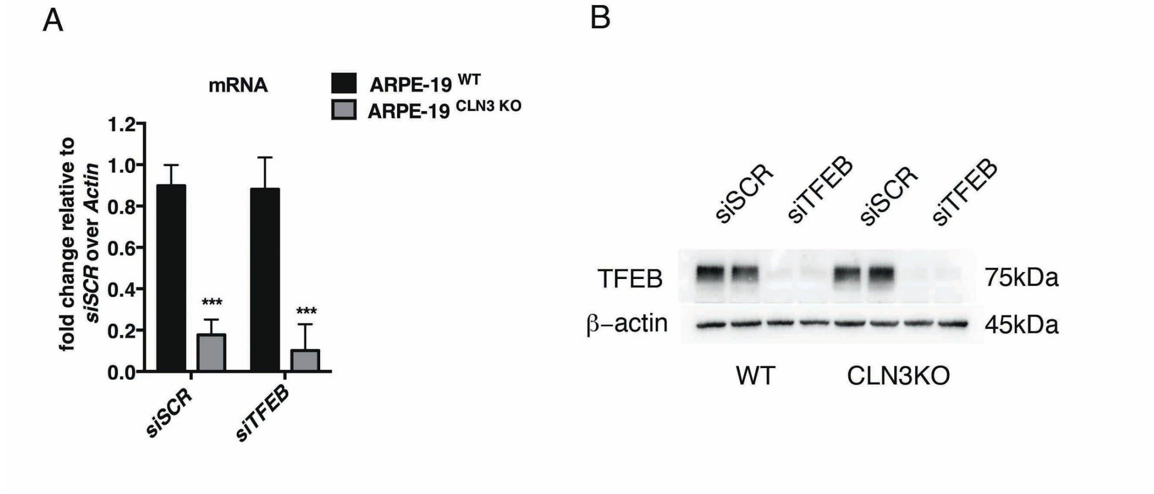

Appendix Figure S7A-B. Image relative to Figure 4. Quantitative PCR and immunoblot of TFEB expression in WT and ARPE 19-CLN3 KO cells transfected with scrambled (*siSCR*) and *TFEB* siRNAs (*siTFEB*). β-actin immunoblotting was performed as loading control. Data are presented as mean ± SD, ***: P ≤ 0.0001, as determined by one-way ANOVA (n=3).

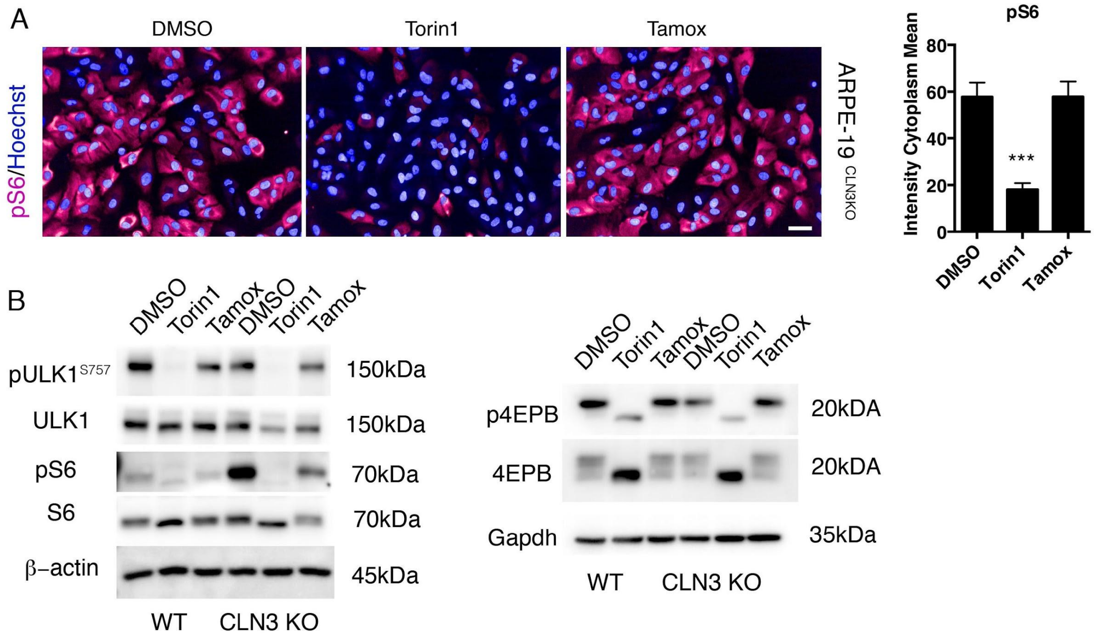

Appendix Figure S8 (A-B). Image relative to Figure 5. Tamoxifen dephosphorylates TFEB. (A) Opera images and quantification of Phospho-S6 ribosomal protein (pS6) intensity levels in ARPE-19 CLN3 KO cells incubated in the absence (DMSO) or presence of torin1 or tamoxifene (Tamox). Data are presented as mean ± SD, ***: $P \leq 0.0001$, as determined by one-way ANOVA (n=3). Scale bars: 40 µm. (B) Immunoblot analysis of the mTORC1 substrates ULK1, p70S6K (S6) and 4EPB in wild type and in ARPE-19 CLN3 KO cells.

Appendix Table S1 Lipidomic raw data relative to Fig 1D

Species Proflie Fig 1D

	rep1	rep2	rep3	rep4
mol%	ARPE WT	ARPE WT	ARPE WT	ARPE WT
HexCer 34:0;2		0,000885425	0,000368422	0,0011947
HexCer 34:1;2	0,041270768	0,043374699	0,04308986	0,043713457
HexCer 36:1;2	0,013156589	0,013977048	0,01353186	0,014307474
HexCer 38:1;2	0,005437953	0,004603391	0,008833425	0,006226619
HexCer 40:1;2	0,056611288	0,067626775	0,064893739	0,06020372
HexCer 40:1;3	0,003819062	0,005358356	0,006956119	0,005162237
HexCer 40:2;2	0,00772388	0,007717706	0,009471497	0,007618555
HexCer 41:1;2	0,018261124	0,016714162	0,016347212	0,018664325
HexCer 41:2;2	0,009731222	0,010271845	0,008012259	0,009795023
HexCer 42:1;2	0,081421297	0,089355224	0,091550572	0,093798536
HexCer 42:1;3	0,004612517	0,004237253	0,005880008	0,006852792
HexCer 42:2;2	0,077394502	0,078616066	0,081746379	0,077107568
HexCer 42:3;2	0,008270697	0,008013283	0,008059413	0,008496674
	0	0	0	0
Hex2Cer 32:1;2	0,001771249	0,003049601	0,002999579	0,001744331
Hex2Cer 34:1;2	0,032914697	0,035598437	0,035585667	0,034335871
Hex2Cer 35:1;2		0,000474502	0,000486375	0,000403517

Hex2Cer 36:1;2	0,012640398	0,012297544	0,012216451	0,010738488
Hex2Cer 38:1;2	0,002783904	0,004570595	0,003663629	0,003339121
Hex2Cer 40:1;2	0,035728338	0,034607907	0,036559584	0,033593756
Hex2Cer 40:2;2	0,009873665	0,011243429	0,009439927	0,008050899
Hex2Cer 41:1;2	0,003047	0,004220329	0,003402466	0,00189736
Hex2Cer 41:2;2	0,003943746	0,006436062	0,004380477	0,004079316
Hex2Cer 42:1;2	0,024659533	0,023689274	0,02436857	0,02458139
Hex2Cer 42:2;2	0,039668969	0,042379118	0,045390081	0,04257562
Hex2Cer 42:3;2	0,001072023	0,004451974	0,006120738	0,002757889
	0	0	0	0
Hex3Cer [34:1:2]	0,202431322	0,124428439	0,139261525	0,16529761
Hex3Cer [34:1:3]	0	0	0	0
Hex3Cer [34:2:2]	0	0	0	0
Hex3Cer [36:1:2]	0,028559602	0,020098241	0,02270247	0,028882791
Hex3Cer [36:2:2]	0	0	0	0
Hex3Cer [38:1:2]		0,003864016	0,005807614	0,010459149
Hex3Cer [40:1:2]	0,143648908	0,092241778	0,115687786	0,112316743
Hex3Cer [40:2:2]	0,01583278	0,010539192	0,027697592	0,031806186
Hex3Cer [41:1:2]		0,009079955	0,001946683	0,008791124
Hex3Cer [41:2:2]	0,012142222		0,002582821	0,006088408
Hex3Cer [42:1:2]	0,172857029	0,150640823	0,119189569	0,136391649
Hex3Cer [42:2:2]	0,320400261	0,194752911	0,20745101	0,239218358
Hex3Cer [42:3:2]	0,009035858	0,018978679	0,00592438	0,018822928
Hex3Cer [43:1:2]	0	0	0	0
Hex3Cer [43:2:2]	0	0	0	0
Hex3Cer [44:1:2]	0	0	0	0
Hex3Cer [44:2:2]	0	0	0	0
	0	0	0	0
GM3 [32:1:2]	0,001367233	0,001480522	0,001659376	0,000929876
GM3 [33:1:2]	0,000978008	0,000597372	0,000827376	0,000618374
GM3 [34:1:2]	0,047480508	0,047282354	0,042622809	0,044569527
GM3 [34:1:3]	0,008893226	0,008515024	0,006719035	0,00663831
GM3 [34:2:2]	0,006960643	0,00512498	0,003514271	0,004074584
GM3 [35:1:2]	0,001459708	0,001707252	0,001707804	0,001289757
GM3 [36:1:2]	0,0160523	0,016466261	0,016167783	0,01427277
GM3 [36:2:2]	0,001476231	0,002252453	0,001774152	0,00215185
GM3 [38:1:2]	0,021283119	0,021596326	0,014776208	0,016226918
GM3 [38:1:3]	0,004389297	0,003042953	0,001357878	0,001286399
GM3 [38:2:2]	0,008074947	0,007689078	0,004435154	0,005400709
GM3 [39:1:2]	0,002375086	0,003185097	0,002775347	0,00215222
GM3 [40:1:2]	0,075501186	0,079674515	0,076497687	0,075153535
GM3 [40:1:3]	0,000850737	0,001065605	0,001134681	0,000507497
GM3 [40:2:2]	0,024293704	0,025653412	0,024622717	0,027240829
GM3 [41:1:2]	0,005864844	0,004846786	0,006617351	0,004313179
GM3 [41:2:2]	0,012929683	0,013289539	0,013123035	0,014515094
GM3 [42:1:2]	0,024907845	0,024937186	0,025732786	0,020607406
GM3 [42:1:3]	0,00206553	0,002108828	0,002634282	0,002752539
GM3 [42:2:2]	0,134606268	0,145373965	0,136404616	0,142087122
GM3 [42:2:3]	0,003152768	0,00132369	0,001754996	0,00115776

GM3 [42:3:2]	0,0219811	0,022701273	0,02154008	0,025204477
GM3 [43:1:2]	0,001037309	0,000537541		
GM3 [43:2:2]	0,002037201	0,00270157	0,002541741	0,001471529
GM3 [44:1:2]	0	0	0	0
GM3 [44:2:2]	0,00298015	0,002644593	0,003368811	0,002932204
	rep1	rep2	rep3	rep4
mol%	ARPE CLN3	ARPE CLN3	ARPE CLN3	ARPE CLN3
HexCer 34:0;2	0,000482575			0,00045534
HexCer 34:1;2	0,026158197	0,024472001	0,024338083	0,025634565
HexCer 36:1;2	0,00968192	0,010922981	0,011128873	0,009670008
HexCer 38:1;2	0,00225018	0,00141367	0,001775749	0,003015224
HexCer 40:1;2	0,030481499	0,030145289	0,029952882	0,031473135
HexCer 40:1;3	0,002306156	0,00216325	0,002441771	0,002973025
HexCer 40:2;2	0,003232288	0,002014789	0,003242849	0,002894676
HexCer 41:1;2	0,007081338	0,007387991	0,007585853	0,00913804
HexCer 41:2;2	0,002252246	0,002825806	0,002535498	0,002190062
HexCer 42:1;2	0,063842697	0,059806332	0,06199586	0,058786571
HexCer 42:1;3	0,001546703	0,001748727	0,001247859	0,001645092
HexCer 42:2;2	0,040785457	0,040018443	0,040241721	0,040648708
HexCer 42:3;2	0,002723044	0,003391916	0,00197021	0,001378864
	0	0	0	0
Hex2Cer 32:1;2	0	0	0	
Hex2Cer 34:1;2	0,072980698	0,067597917	0,068668021	0,06520586
Hex2Cer 35:1;2	0,003909887	0,004008721	0,002940156	0,003512784
Hex2Cer 36:1;2	0,027496669	0,02171164	0,025947062	0,020627785
Hex2Cer 38:1;2	0,002874393	0,003057722	0,003096167	0,001774133
Hex2Cer 40:1;2	0,04350744	0,041888865	0,046823725	0,038681509
Hex2Cer 40:2;2	0,001496322	0,00244395	0,003009123	0,003262256
Hex2Cer 41:1;2	0,011742213	0,018888486	0,017925831	0,015861531
Hex2Cer 41:2;2	0,003650248	0,002410797	0,001780442	0,003990899
Hex2Cer 42:1;2	0,096191138	0,094034639	0,105091169	0,097695538
Hex2Cer 42:2;2	0,05070508	0,049559894	0,05271765	0,047972685
Hex2Cer 42:3;2	0,003368352	0,005645645	0,004132864	0,005345035
	0	0	0	0
Hex3Cer [34:1:2]	0,264824024	0,228146488	0,214278905	0,290058062
Hex3Cer [34:1:3]	0,00416199	0,006051294	0,004588918	0,005435362
Hex3Cer [34:2:2]		0,002095693	0,002785728	0,006193732
Hex3Cer [36:1:2]	0,084680026	0,079963467	0,064692797	0,093169797
Hex3Cer [36:2:2]		0,002766925	0,005340359	0,005333322
Hex3Cer [38:1:2]	0,018159674	0,014577858	0,017240355	0,019287823
Hex3Cer [40:1:2]	0,177722022	0,159072267	0,139509827	0,198888448
Hex3Cer [40:2:2]	0,069257545	0,055681572	0,050697589	0,053805971
Hex3Cer [41:1:2]	0,034671786	0,032345424	0,020275705	0,037428576
Hex3Cer [41:2:2]	0,024350766	0,011264486	0,011809308	0,023336061
Hex3Cer [42:1:2]	0,404508918	0,342831923	0,311810772	0,4110031
Hex3Cer [42:2:2]	0,385528315	0,329806532	0,294890859	0,396003136
Hex3Cer [42:3:2]	0,0584243	0,042482287	0,058206924	0,063643737
Hex3Cer [43:1:2]	0,003175044	0,010245436		0,002756933

Hex3Cer [43:2:2]	0,004416025	0,003193401	0,007288784	0,004016161
Hex3Cer [44:1:2]		0,001576242	0,003093282	
Hex3Cer [44:2:2]	0,003432767	0,00140622	0,004365094	0
	0	0	0	0
GM3 [32:1:2]	0,00130833	0,000811985	0,001373758	0,001354437
GM3 [33:1:2]	0,000797393	0,001104209	0,0013007	0,001172831
GM3 [34:1:2]	0,058198109	0,062533082	0,064117974	0,065124205
GM3 [34:1:3]	0,004882798	0,006223284	0,00701539	0,008035427
GM3 [34:2:2]	0,005621146	0,00775884	0,007738529	0,009278073
GM3 [35:1:2]	0,004876732	0,005587193	0,005842947	0,005091444
GM3 [36:1:2]	0,017695666	0,0181975	0,020371192	0,01853643
GM3 [36:2:2]	0,004433949	0,00259987	0,0021861	0,002044455
GM3 [38:1:2]	0,013869587	0,02182587	0,017656103	0,017554772
GM3 [38:1:3]	0,001592446	0,006907442	0,004321634	0,003834473
GM3 [38:2:2]	0,005012441	0,007120625	0,005857985	0,007322062
GM3 [39:1:2]	0,00253741	0,002577361	0,002870406	0,002597738
GM3 [40:1:2]	0,054657898	0,055393197	0,058743584	0,057303987
GM3 [40:1:3]			0,000259907	6,41811E-05
GM3 [40:2:2]	0,0114948	0,010914726	0,011895347	0,011263324
GM3 [41:1:2]	0,011169403	0,009855836	0,010367031	0,011482863
GM3 [41:2:2]	0,007071582	0,006447329	0,00715992	0,006948586
GM3 [42:1:2]	0,075059765	0,111324531	0,120657396	0,121071603
GM3 [42:1:3]	0,00055518	0,000645438	0,001282408	0,001126659
GM3 [42:2:2]	0,213016477	0,084205059	0,085829969	0,084572118
GM3 [42:2:3]	0,009207	0,009417374	0,00462011	0,005728774
GM3 [42:3:2]	0,01112398	0,010883977	0,011821727	0,011593906
GM3 [43:1:2]	0,006616425	0,006468073	0,006460194	0,00578533
GM3 [43:2:2]	0,002876291	0,001557383	0,002527022	0,002985128
GM3 [44:1:2]	0,002642471	0,00211419	0,00289473	0,003182262
GM3 [44:2:2]	0,003137143	0,002150912	0,00270852	0,002990442
	rep1	rep2	rep3	rep4
mol%	ARPE CLN7	ARPE CLN7	ARPE CLN7	ARPE CLN7
HexCer 34:0;2			0,000412202	0,000392347
HexCer 34:1;2	0,025136445	0,021341594	0,023294594	0,020425915
HexCer 36:1;2	0,01134874	0,008826281	0,0089845	0,007947539
HexCer 38:1;2	0,001041992	0,001431412	0,000690622	0,001422739
HexCer 40:1;2	0,023719956	0,020332144	0,023170354	0,021451838
HexCer 40:1;3	0,000361036	0,00049793	0,000545404	0,000801791
HexCer 40:2;2		0,000778282		0,000555694
HexCer 41:1;2	0,007077765	0,007071113	0,006246886	0,00628002
HexCer 41:2;2	0,001066203	0,001212718	0,000793041	
HexCer 42:1;2	0,062809432	0,055023167	0,056974376	0,052905648
HexCer 42:1;3	0	0	0	0
HexCer 42:2;2	0,027819224	0,027051709	0,027303477	0,023964141
HexCer 42:3;2	0,001366924	0,000333841	0,00042708	0,001257881
	0	0	0	0
Hex2Cer 32:1;2	0,001825875		0,003460892	

Hex2Cer 34:1;2	0,077528186	0,068446476	0,06061166	0,061247186
Hex2Cer 35:1;2	0,006219272	0,00399312	0,002966259	0,004034125
Hex2Cer 36:1;2	0,022481392	0,021925601	0,019581176	0,019343531
Hex2Cer 38:1;2	0,003489687	0,002322805	0,003461722	0,002668342
Hex2Cer 40:1;2	0,04457701	0,039948952	0,037724644	0,037732258
Hex2Cer 40:2;2	0,00280049	0,001364839	0,002709331	0,001120603
Hex2Cer 41:1;2	0,0160349	0,014528177	0,015364458	0,014903477
Hex2Cer 41:2;2	0,003467283	0,005064293	0,002827068	0,002122005
Hex2Cer 42:1;2	0,107004502	0,087355712	0,083725275	0,083596063
Hex2Cer 42:2;2	0,04830812	0,043958186	0,045053404	0,042336044
Hex2Cer 42:3;2	0,00176927	0,003045157	0,001680011	0,001741703
	0	0	0	0
Hex3Cer [34:1:2]	0,263754893	0,261221124	0,225935474	0,231548565
Hex3Cer [34:1:3]			0,004323583	0,002701141
Hex3Cer [34:2:2]	0,005519217		0,00268835	0,005126251
Hex3Cer [36:1:2]	0,099790924	0,074863413	0,065642038	0,086070351
Hex3Cer [36:2:2]	0,002803565	0,003232066	0,003375882	0,003374378
Hex3Cer [38:1:2]	0,017856967	0,003622895	0,010797662	0,01490464
Hex3Cer [40:1:2]	0,167206607	0,154585248	0,14242597	0,157309368
Hex3Cer [40:2:2]	0,030942127	0,030147323	0,026433444	0,032631368
Hex3Cer [41:1:2]	0,033563968	0,02983566	0,023932972	0,037611118
Hex3Cer [41:2:2]	0,013779454	0,006650595	0,008432685	0,012345415
Hex3Cer [42:1:2]	0,464409611	0,454355791	0,371796339	0,410662643
Hex3Cer [42:2:2]	0,293994585	0,300651902	0,251681036	0,279228812
Hex3Cer [42:3:2]	0,02926203	0,021297229	0,024510739	0,024451494
Hex3Cer [43:1:2]	0,005760575	0,012945243	0,011028586	0,008895105
Hex3Cer [43:2:2]	0,006936943	0,003733585	0,002736333	0,007890659
Hex3Cer [44:1:2]	0,002497584		0,001802379	0,004060454
Hex3Cer [44:2:2]	0,009505892	0,004414325	0,007894389	0,002947287
	0	0	0	0
GM3 [32:1:2]	0,001076079	0,00112428	0,001274168	0,001105142
GM3 [33:1:2]	0,001282829	0,001558527		
GM3 [34:1:2]	0,056232591	0,054907516	0,054277759	0,051357834
GM3 [34:1:3]	0,004973988	0,003385669	0,004996943	0,004905096
GM3 [34:2:2]	0,004677233	0,006856864	0,004763096	0,004400054
GM3 [35:1:2]	0,00454455	0,004461128	0,00476848	0,004801763
GM3 [36:1:2]	0,018441689	0,016693522	0,016032647	0,016639188
GM3 [36:2:2]	0,002183202	0,002157031	0,00230144	0,002012415
GM3 [38:1:2]	0,016177235	0,017993858	0,01888451	0,014467481
GM3 [38:1:3]	0,001696013	0,003022906	0,005188161	0,003519286
GM3 [38:2:2]	0,004844709	0,006085475	0,00459641	0,003870843
GM3 [39:1:2]	0,002452041	0,001505197	0,001862456	0,001762899
GM3 [40:1:2]	0,049151998	0,044451494	0,0435371	0,042584831
GM3 [40:1:3]	2,7359E-05			0,00013725
GM3 [40:2:2]	0,00835777	0,007494913	0,008159698	0,007885461
GM3 [41:1:2]	0,009945983	0,009320812	0,00790804	0,008977482
GM3 [41:2:2]	0,005321157	0,003138313	0,004736552	0,004432791
GM3 [42:1:2]	0,110225853	0,097425657	0,093543757	0,097395434
GM3 [42:1:3]	0,000432271			0,000642495

GM3 [42:2:2]	0,067357302	0,059981168	0,063221643	0,060334134
GM3 [42:2:3]	0,008896851	0,034518136	0,011866996	0,009431699
GM3 [42:3:2]	0,009407101	0,007636433	0,00821907	0,008131043
GM3 [43:1:2]	0,005925201	0,005039852	0,005047529	0,004955065
GM3 [43:2:2]	0,001549323	0,000809728	0,00131845	0,001925604
GM3 [44:1:2]	0,002595155	0,002338427	0,002415308	0,001944508
GM3 [44:2:2]	0,002422729	0,002461196	0,002573112	0,002562931

	average	average	average	stdev	stdev	stdev
mol%	ARPE WT	ARPE CLN3	ARPE CLN7	ARPE WT	ARPE CLN3	ARPE CLN7
HexCer 34:0;2	0,000816182	0,000468958	0,000402274	0,000417468	1,9258E-05	1,40394E-05
HexCer 34:1;2	0,042862196	0,025150712	0,022549637	0,001091143	0,000888848	0,002098897
HexCer 36:1;2	0,013743243	0,010350945	0,009276765	0,00050394	0,000783936	0,001454679
HexCer 38:1;2	0,006275347	0,002113706	0,001146691	0,001829645	0,000691767	0,000354132
HexCer 40:1;2	0,06233388	0,030513201	0,022168573	0,004893985	0,000676214	0,001559544
HexCer 40:1;3	0,005323944	0,002471051	0,00055154	0,001285302	0,000353443	0,000184236
HexCer 40:2;2	0,008132909	0,00284615	0,000666988	0,000893696	0,000577347	0,000157393
HexCer 41:1;2	0,017496706	0,007798306	0,006668946	0,00113745	0,000916956	0,000468426
HexCer 41:2;2	0,009452587	0,002450903	0,001023987	0,000990055	0,000291668	0,000213
HexCer 42:1;2	0,089031407	0,061107865	0,056928156	0,00538796	0,002262022	0,004258368
HexCer 42:1;3	0,005395642	0,001547095	0	0,001199034	0,000215871	0
HexCer 42:2;2	0,078716129	0,040423582	0,026534638	0,002123408	0,000355362	0,001743186
HexCer 42:3;2	0,008210017	0,002366009	0,000846431	0,00022154	0,0008777	0,000541236
	0	0	0	0	0	0
Hex2Cer 32:1;2	0,00239119	0	0,002643383	0,000731755	0	0,001156132
Hex2Cer 34:1;2	0,034608668	0,068613124	0,066958377	0,001275164	0,003251605	0,007891637
Hex2Cer 35:1;2	0,000454798	0,003592887	0,004303194	4,48055E-05	0,000485071	0,001369585
Hex2Cer 36:1;2	0,01197322	0,023945789	0,020832925	0,000843413	0,003297192	0,001601723
Hex2Cer 38:1;2	0,003589312	0,002700604	0,002985639	0,000748268	0,000625181	0,000583308

Hex2Cer 40:1;2	0,035122396	0,042725385	0,039995716	0,001295391	0,003389233	0,003228594
Hex2Cer 40:2;2	0,00965198	0,002552913	0,001998816	0,00131533	0,000783061	0,000879526
Hex2Cer 41:1;2	0,003141789	0,016104515	0,015207753	0,000964156	0,00317051	0,00064888
Hex2Cer 41:2;2	0,0047099	0,002958096	0,003370162	0,001165158	0,001037973	0,001255967
Hex2Cer 42:1;2	0,024324692	0,098253121	0,090420388	0,000441095	0,004799902	0,011192572
Hex2Cer 42:2;2	0,042503447	0,050238827	0,044913938	0,002337206	0,001996522	0,002523154
Hex2Cer 42:3;2	0,003600656	0,004622974	0,002059035	0,002174074	0,001061683	0,000658473
	0	0	0	0	0	0
Hex3Cer [34:1:2]	0,157854724	0,24932687	0,245615014	0,034183296	0,034526127	0,019644806
Hex3Cer [34:1:3]	0	0,005059391	0,003512362	0	0,000846935	0,00114724
Hex3Cer [34:2:2]	0	0,003691718	0,004444606	0	0,002194104	0,001533601
Hex3Cer [36:1:2]	0,025060776	0,080626522	0,081591682	0,00436035	0,011945541	0,014730146
Hex3Cer [36:2:2]	0	0,004480202	0,003196473	0	0,001483745	0,000270482
Hex3Cer [38:1:2]	0,00671026	0,017316427	0,011795541	0,003388956	0,002008566	0,006169692
Hex3Cer [40:1:2]	0,115973804	0,168798141	0,155381798	0,021154797	0,02541299	0,010198523
Hex3Cer [40:2:2]	0,021468937	0,057360669	0,030038565	0,009947872	0,00819324	0,002617109
Hex3Cer [41:1:2]	0,006605921	0,031180373	0,03123593	0,004037602	0,007560845	0,005812536
Hex3Cer [41:2:2]	0,006937817	0,017690156	0,010302037	0,004835975	0,007120711	0,003321412
Hex3Cer [42:1:2]	0,144769768	0,367538678	0,425306096	0,022714922	0,048207807	0,042625089
Hex3Cer [42:2:2]	0,240455635	0,35155721	0,281389084	0,056482205	0,047657261	0,021734686
Hex3Cer [42:3:2]	0,013190461	0,055689312	0,024880373	0,006715276	0,009156362	0,003284225
Hex3Cer [43:1:2]	0	0,005392471	0,009657377	0	0,004207987	0,003079848
Hex3Cer [43:2:2]	0	0,004728592	0,00532438	0	0,001781072	0,00247755
Hex3Cer [44:1:2]	0	0,002334762	0,002786806	0	0,001072709	0,001156487
Hex3Cer [44:2:2]	0	0,00230102	0,006190473	0	0,001969485	0,003031407
	0	0	0	0	0	0

GM3 [32:1:2]	0,001359252	0,001212128	0,001144917	0,000310488	0,00026817	8,84163E-05
GM3 [33:1:2]	0,000755282	0,001093783	0,001420678	0,000181185	0,000213713	0,000194948
GM3 [34:1:2]	0,0454888	0,062493342	0,054193925	0,002326851	0,003055677	0,002058787
GM3 [34:1:3]	0,007691399	0,006539225	0,004565424	0,001180005	0,001330279	0,000787471
GM3 [34:2:2]	0,00491862	0,007599147	0,005174312	0,00151625	0,001502909	0,001132349
GM3 [35:1:2]	0,00154113	0,005349579	0,00464398	0,000204283	0,000443505	0,00016705
GM3 [36:1:2]	0,015739779	0,018700197	0,016951762	0,000993437	0,001166309	0,001037472
GM3 [36:2:2]	0,001913672	0,002816094	0,002163522	0,000356988	0,001104012	0,000118721
GM3 [38:1:2]	0,018470643	0,017726583	0,016880771	0,003481526	0,003251413	0,001964083
GM3 [38:1:3]	0,002519132	0,004163999	0,003356591	0,001487733	0,002181193	0,001443374
GM3 [38:2:2]	0,006399972	0,006328278	0,004849359	0,001763176	0,001090569	0,000921861
GM3 [39:1:2]	0,002621938	0,002645728	0,001895648	0,000455431	0,000151866	0,000400312
GM3 [40:1:2]	0,076706731	0,056524666	0,044931356	0,002058899	0,001852565	0,002915145
GM3 [40:1:3]	0,00088963	0,000162044	8,23048E-05	0,00028199	0,000138399	7,7705E-05
GM3 [40:2:2]	0,025452665	0,011392049	0,007974461	0,001325372	0,000411612	0,000373776
GM3 [41:1:2]	0,00541054	0,010718783	0,009038079	0,00103034	0,000742806	0,000853402
GM3 [41:2:2]	0,013464338	0,006906854	0,004407203	0,000715772	0,000318372	0,000922771
GM3 [42:1:2]	0,024046306	0,107028324	0,099647675	0,002324232	0,021782343	0,0072839
GM3 [42:1:3]	0,002390295	0,000902421	0,000537383	0,000353764	0,000356506	0,00014865
GM3 [42:2:2]	0,139617993	0,116905906	0,062723562	0,004989021	0,064077492	0,003413196
GM3 [42:2:3]	0,001847303	0,007243315	0,01617842	0,00090598	0,002432943	0,01229462
GM3 [42:3:2]	0,022856732	0,011355898	0,008348412	0,001636702	0,000428229	0,000750936
GM3 [43:1:2]	0,000787425	0,006332505	0,005241912	0,000353389	0,000371795	0,000457449
GM3 [43:2:2]	0,00218801	0,002486456	0,001400776	0,000555265	0,000649479	0,000466771
GM3 [44:1:2]	0	0,002708413	0,002323349	0	0,000453393	0,000274519

GM3 [44:2:2]	0,002981439	0,002746754	0,002504992	0,00029774	0,000435231	7,45709E-05

Appendix Table S2 Lipidomic raw data relative to Fig 1D

Class profiel Fig 1D

	rep1	rep2	rep3	rep4		
mol%	ARPE WT	ARPE WT	ARPE WT	ARPE WT		
GlcCer	0,327710898	0,350751234	0,358740765	0,353141681		
LacCer	0,209853254	0,219443474	0,222697468	0,198505158		
Gb3	0,904907981	0,624624034	0,648251448	0,758074946		
Gb4	0,248936985	0,38435308	0,311742014	0,275140889		
GM3	0,432998629	0,445798178	0,414309978	0,417554464		
	rep1	rep2	rep3	rep4		
mol%	ARPE CLN3	ARPE CLN3	ARPE CLN3	ARPE CLN3		
GlcCer	0,192824298	0,186311196	0,188457208	0,189903309		
LacCer	0,351436588	0,352617304	0,367360757	0,354053187		
Gb3	1,537313204	1,323507515	1,210875206	1,610360222		
Gb4	0,012072461	0,011698575	0,006408577	0,007749995		
GM3	0,529454419	0,454625287	0,467880581	0,468045511		
	rep1	rep2	rep3	rep4		
mol%	ARPE CLN7	ARPE CLN7	ARPE CLN7	ARPE CLN7		
GlcCer	0,161747716	0,143900192	0,148842536	0,137405552		
LacCer	0,356852919	0,308858283	0,300886939	0,295375461		
Gb3	1,447584943	1,361556401	1,18543786	1,321759048		
Gb4	0,025955261	0,012975688	0,015091609	0,031826845		
GM3	0,400198211	0,394368102	0,372267178	0,361164005		
	average	average	average	st dev	st dev	st dev
mol%	ARPE WT	ARPE CLN3	ARPE CLN7	ARPE WT	ARPE CLN3	ARPE CLN7
GlcCer	0,347586145	0,189374003	0,147973999	0,01366666	0,002732886	0,010307887
LacCer	0,212624838	0,356366959	0,3154934	0,010878093	0,00740688	0,028123032
Gb3	0,733964602	1,420514037	1,329084563	0,127938855	0,185330627	0,109216602
Gb4	0,305043242	0,009482402	0,021462351	0,058813226	0,002832517	0,008948348
GM3	0,427665312	0,48000145	0,381999374	0,014581258	0,033562905	0,018375812

Appendix Table S3 lipidomic raw data relative to Fig 2E

Species Proflie Fig 2E

	rep1	rep2	rep3	rep1	rep2	rep3
mol%	WT	WT	WT	CLN7 KO	CLN7 KO	CLN7 KO

HexCer [32:0:2]	0,005613334	0,005136592	0,018395928	0,073295371	0,055245835	0,053124101
HexCer [32:1:2]	0,026447297	0,02132786	0,043147753	0,078858644	0,116384064	0,088197314
HexCer [33:1:2]	0,017251365	0,009264039	0,009786812	0,025311833	0,040584353	0,057769273
HexCer [34:1:2]	0,022579116	0,011024399	0,011494511	0,034062415	0,042264134	0,058106656
HexCer [34:1:3]	0,013933926	0,011442716	0,030929526	0,041138528	0,064538952	0,045147637
HexCer [36:1:2]	0,08361586	0,081237409	0,081265948	0,147224949	0,156242651	0,166234759
HexCer [36:1:3]	0,095827023	0,09667347	0,103556884	0,117776471	0,101155422	0,123400037
HexCer [36:2:2]	0,032214698	0,029750074	0,028733962	0,048804503	0,041683663	0,038629798
HexCer [37:1:2]	0,014701645	0,006650363	0,009431153	0,03823003	0,04323679	0,052950918
HexCer [38:1:2]	0,068074325	0,039401954	0,045904589	0,073629604	0,095783634	0,103734629
HexCer [38:1:3]	0,158749336	0,151555623	0,160566488	0,172221287	0,15657904	0,165004837
HexCer [38:2:2]	0,048390599	0,041473455	0,041074785	0,047258773	0,054031904	0,04875725
HexCer [38:2:3]	0,014812768	0,005158893	0,00968482	0,002060131	0,005201386	
HexCer [39:1:2]	0,017998113	0,00674354	0,006539685	0,010302223	0,008489664	0,013276818
HexCer [39:1:3]	0,062254843	0,05416781	0,059653422	0,048286729	0,051569757	0,056973325
HexCer [39:2:2]	0,011285992	0,007118539	0,008863177	0,002018691	0,007397798	0,003821486
HexCer [40:0:2]	0,02470026	0,023411461	0,049516071	0,12500768	0,083490464	0,085486819
HexCer [40:0:3]	0,131826549	0,131313011	0,138718608	0,177711726	0,174024233	0,181244
HexCer [40:1:2]	0,18712853	0,179131	0,190199904	0,227612786	0,258788509	0,26426234
HexCer [40:1:3]	1,436316971	1,439879408	1,536595942	1,632698875	1,55077245	1,659749867
HexCer [40:2:2]	0,399892312	0,371138609	0,367687117	0,412517045	0,398690434	0,411355329
HexCer [40:2:3]	0,166814213	0,152168041	0,163724462	0,134732741	0,136320786	0,132818493
HexCer [40:3:2]	0,048627282	0,032894391	0,03573789	0,019819264	0,030717958	0,034202706
HexCer [41:0:3]	0,018797457	0,018847767	0,020679684	0,00847949	0,017726053	0,018830705
HexCer [41:1:2]	0,121465153	0,115018799	0,112330704	0,131057163	0,128985771	0,150513769

HexCer [41:1:3]	0,677606844	0,657153574	0,695608949	0,653504538	0,636877552	0,67712329
HexCer [41:2:2]	0,184772454	0,183201793	0,168566813	0,181214444	0,184654481	0,172544791
HexCer [41:2:3]	0,066680398	0,057970853	0,068606847	0,046152154	0,043063518	0,03973679
HexCer [41:3:2]	0,018895539	0,010653283	0,014655391	0,0030287	0,005704281	0,00989665
HexCer [42:0:2]	0,016069673	0,019332932	0,061002012	0,17464634	0,094804796	0,064914904
HexCer [42:0:3]	0,044903619	0,034988875	0,045154212	0,05601116	0,0365605	0,058925927
HexCer [42:1:2]	0,369701111	0,352010636	0,369495965	0,462685064	0,458165962	0,481934658
HexCer [42:1:3]	1,321982535	1,336751286	1,37118667	1,580220304	1,509863631	1,586566319
HexCer [42:2:2]	1,678806569	1,658620293	1,690685379	1,779755071	1,759512315	1,781977884
HexCer [42:2:3]	0,91312887	0,903582371	0,919941177	0,899756824	0,87012506	0,930963463
HexCer [42:3:2]	0,270442125	0,242882056	0,242098682	0,225170194	0,242415642	0,250384441
HexCer [42:3:3]	0,061902646	0,050512729	0,054486665	0,039132833	0,050296954	0,040055965
HexCer [43:1:3]	0,078626887	0,067102418	0,058300941	0,067607409	0,08090775	0,10556924
HexCer [43:2:2]	0,054153496	0,054115763	0,056180951	0,057667229	0,061922535	0,047956319
HexCer [43:2:3]	0,048771302	0,038686303	0,035589121	0,019677122	0,040184993	0,031557961
HexCer [43:3:2]	0,013365142	0,00670677	0,003296764	0	0	0
HexCer [44:1:3]	0,039763445	0,03742902	0,029939667	0,039249253	0,059130055	0,072646971
HexCer [44:2:2]	0,013806708	0,007741646	0,014432458	0,01571201	0,008338534	0,013661305
HexCer [44:2:3]	0,030809773	0,020711752	0,020940566	0,031572541	0,028397431	0,023364641
	0	0	0	0	0	0
Hex3Cer [34:1:2]	0,014118665	0,016546912	0,012056412	0,082319435	0,092136617	0,095134989
Hex3Cer [36:1:3]	0	0		0,006174876	0,003448972	0,003713949
Hex3Cer [38:1:2]	0	0	0	0,004064201		0,006612586
Hex3Cer [40:1:2]	0	0	0	0,036355584	0,049492657	0,046677301
Hex3Cer [40:2:2]	0,001530198	0,004060724		0,030774685	0,059139841	0,009843976
Hex3Cer	0	0	0		0,00405692	0,00353865

[40:2:3]					2	1
Hex3Cer [41:2:2]	0,001145183	0,004227614	0,00144021	0,036231898	0,053102391	0,004203201
Hex3Cer [42:1:2]	0	0	0	0,045587925	0,042256248	0,038118771
Hex3Cer [42:2:2]	0,001490698	0,004219529	0,001293269	0,153143653	0,159278424	0,133454185
Hex3Cer [42:3:2]	0	0	0	0,025507582	0,020295488	0,015954792
Hex3Cer [43:2:2]		0	0	0,006010485	0,019887722	0,00269375
	0	0	0	0	0	0
GM3 [34:1:2]	0	0	0	0,021743164		0,060064855
GM3 [36:1:2]	0,036286975	0,038272566	0,036051493	0,441246612	0,437059582	0,345879079
GM3 [36:1:3]	0	0	0	0,001004674		0,00438841
GM3 [36:2:2]	0	0	0	0,02715056	0,026337382	0,006397427
GM3 [38:1:2]	0	0	0	0,097884473	0,090195185	0,090354034
GM3 [38:2:2]	0	0	0	0,004139146		0,017072564
GM3 [39:1:2]	0,000121091	0,001543543	0,000576456	0,011677565	0,012567799	
GM3 [40:1:2]	0,002965152	0,010165948	0,006833339	0,082580966	0,085887607	0,024669414
GM3 [40:1:3]	0,000858271	0,001478942	0,00115927	0,005633939	0,009929471	0
GM3 [40:2:2]	0	0		0,007129852	0,003997327	
GM3 [41:2:2]	0	0	0	0,00199162	0,002759658	0,003326156
GM3 [42:2:2]	0	0	0	0,047970315	0,039644636	0,033197656

	average	average	stdev	stdev
mol%	WT	CLN7 KO	WT	CLN7 KO
HexCer [32:0:2]	0,009715284	0,060555102	0,007521436	0,01108428
HexCer [32:1:2]	0,030307636	0,094480007	0,011410679	0,019535697
HexCer [33:1:2]	0,012100739	0,04122182	0,004468225	0,016238107
HexCer [34:1:2]	0,015032675	0,044811068	0,006539635	0,012222788
HexCer [34:1:3]	0,018768723	0,050275039	0,01060497	0,012514497
HexCer [36:1:2]	0,082039739	0,156567453	0,001365035	0,009509067
HexCer [36:1:3]	0,098685792	0,114110643	0,004239666	0,011566523
HexCer [36:2:2]	0,030232912	0,043039321	0,001789897	0,005221064
HexCer [37:1:2]	0,010261054	0,044805913	0,004089296	0,007484834
HexCer [38:1:2]	0,051126956	0,091049289	0,015032666	0,015600918

HexCer [38:1:3]	0,156957149	0,164601721	0,004765278	0,007828911
HexCer [38:2:2]	0,043646279	0,050015976	0,004113533	0,003557685
HexCer [38:2:3]	0,009885494	0,003630759	0,004830065	0,002221202
HexCer [39:1:2]	0,010427113	0,010689568	0,006557471	0,002416969
HexCer [39:1:3]	0,058692025	0,052276604	0,004128345	0,004386224
HexCer [39:2:2]	0,009089236	0,004412658	0,002092903	0,002737848
HexCer [40:0:2]	0,032542597	0,097994988	0,014713577	0,023414963
HexCer [40:0:3]	0,133952722	0,177659986	0,004135357	0,003610162
HexCer [40:1:2]	0,185486478	0,250221212	0,005714229	0,019769835
HexCer [40:1:3]	1,470930774	1,614407064	0,056895593	0,05674471
HexCer [40:2:2]	0,379572679	0,407520936	0,017681737	0,007669467
HexCer [40:2:3]	0,160902239	0,134624007	0,007720188	0,001753676
HexCer [40:3:2]	0,039086521	0,028246643	0,00838397	0,007503426
HexCer [41:0:3]	0,019441636	0,015012083	0,001072476	0,005684289
HexCer [41:1:2]	0,116271552	0,136852234	0,004694314	0,011876481
HexCer [41:1:3]	0,676789789	0,655835126	0,019240703	0,020223837
HexCer [41:2:2]	0,17884702	0,179471239	0,00893749	0,00624021
HexCer [41:2:3]	0,064419366	0,042984154	0,005667036	0,003208418
HexCer [41:3:2]	0,014734738	0,006209877	0,004121701	0,003461778
HexCer [42:0:2]	0,032134873	0,111455347	0,025052865	0,056728984
HexCer [42:0:3]	0,041682235	0,050499196	0,005797974	0,012158922
HexCer [42:1:2]	0,363735904	0,467595228	0,010154898	0,012622203
HexCer [42:1:3]	1,34330683	1,558883418	0,025248626	0,042570795
HexCer [42:2:2]	1,676037414	1,773748423	0,01621091	0,012378825
HexCer [42:2:3]	0,912217473	0,900281782	0,008217397	0,030422598
HexCer [42:3:2]	0,251807621	0,239323426	0,016142707	0,012888402
HexCer [42:3:3]	0,055634013	0,043161917	0,005780991	0,006196338
HexCer [43:1:3]	0,068010082	0,084694799	0,010193327	0,019262177
HexCer [43:2:2]	0,054816736	0,055848694	0,001181595	0,007158499
HexCer [43:2:3]	0,041015575	0,030473359	0,006892867	0,010296867
HexCer [43:3:2]	0,007789559	0	0,005120779	0
HexCer [44:1:3]	0,03571071	0,05700876	0,005132358	0,016799608
HexCer [44:2:2]	0,011993604	0,012570616	0,003695572	0,003805817
HexCer [44:2:3]	0,02415403	0,027778204	0,005765177	0,004138839
	0	0	0	0
Hex3Cer [34:1:2]	0,014240663	0,08986368	0,002247734	0,006703304
Hex3Cer [36:1:3]	0	0,004445932	0	0,001503159
Hex3Cer [38:1:2]	0	0,005338394	0	0,00180198
Hex3Cer [40:1:2]	0	0,044175181	0	0,006916728
Hex3Cer [40:2:2]	0,002795461	0,033252834	0,001789352	0,02474119
Hex3Cer [40:2:3]	0	0,003797787	0	0,000366473
Hex3Cer [41:2:2]	0,002271002	0,031179163	0,001700884	0,024838081
Hex3Cer [42:1:2]	0	0,041987648	0	0,003741814
Hex3Cer [42:2:2]	0,002334498	0,148625421	0,001635466	0,013491984
Hex3Cer [42:3:2]	0	0,020585954	0	0,004783014
Hex3Cer [43:2:2]	0	0,009530652	0	0,009121505
	0	0	0	0
GM3 [34:1:2]	0	0,040904009	0	0,027097527
GM3 [36:1:2]	0,036870345	0,408061757	0,001220054	0,053892457

GM3 [36:1:3]	0	0,002696542	0	0,002392663
GM3 [36:2:2]	0	0,01996179	0	0,011754117
GM3 [38:1:2]	0	0,092811231	0	0,004394274
GM3 [38:2:2]	0	0,010605855	0	0,009145307
GM3 [39:1:2]	0,00074703	0,012122682	0,000726405	0,000629491
GM3 [40:1:2]	0,006654813	0,064379329	0,003603716	0,034429514
GM3 [40:1:3]	0,001165494	0,005187803	0,000310382	0,004979747
GM3 [40:2:2]	0	0,00556359	0	0,002215029
GM3 [41:2:2]	0	0,002692478	0	0,0006698
GM3 [42:2:2]	0	0,040270869	0	0,007406213

Appendix Table S4 Lipidomic raw data relative to Fig3E

Class profiel Fig 3E

	rep1	rep2	rep3	rep1	rep2	rep3
mol%	WT 1	WT 2	WT 3	CLN7 KO 1	CLN7 KO 2	CLN7 KO 3
GlcCer	9,133508099	8,782083574	9,204389054	10,16288014	9,990831694	10,40337438
Gb3	0,018284744	0,029054779	0,014789891	0,426170324	0,503095281	0,359946152
GM3	0,040231489	0,051461	0,044620558	0,750152885	0,708378647	0,585349595
	average	average	stdev	stdev		
	WT	CLN7 KO	WT	CLN7 KO		
GlcCer	9,039993576	10,18569541	0,226150844	0,207215516		
Gb3	0,020709805	0,429737252	0,007435216	0,071641193		
GM3	0,045437682	0,681293709	0,005659174	0,085675122		
