# Supplementary material for: Repurposing of tamoxifen ameliorates CLN3 and CLN7 disease phenotype
Source: EMBO Mol Med. 2021 Aug 19;13(10):e13742. doi: 10.15252/emmm.202013742 (PMC8495452; doi:10.15252/emmm.202013742)
Supplement: Supplementary file 2 — Expanded View Figures PDF [file EMMM-13-e13742-s009.pdf]

Expanded View Figures

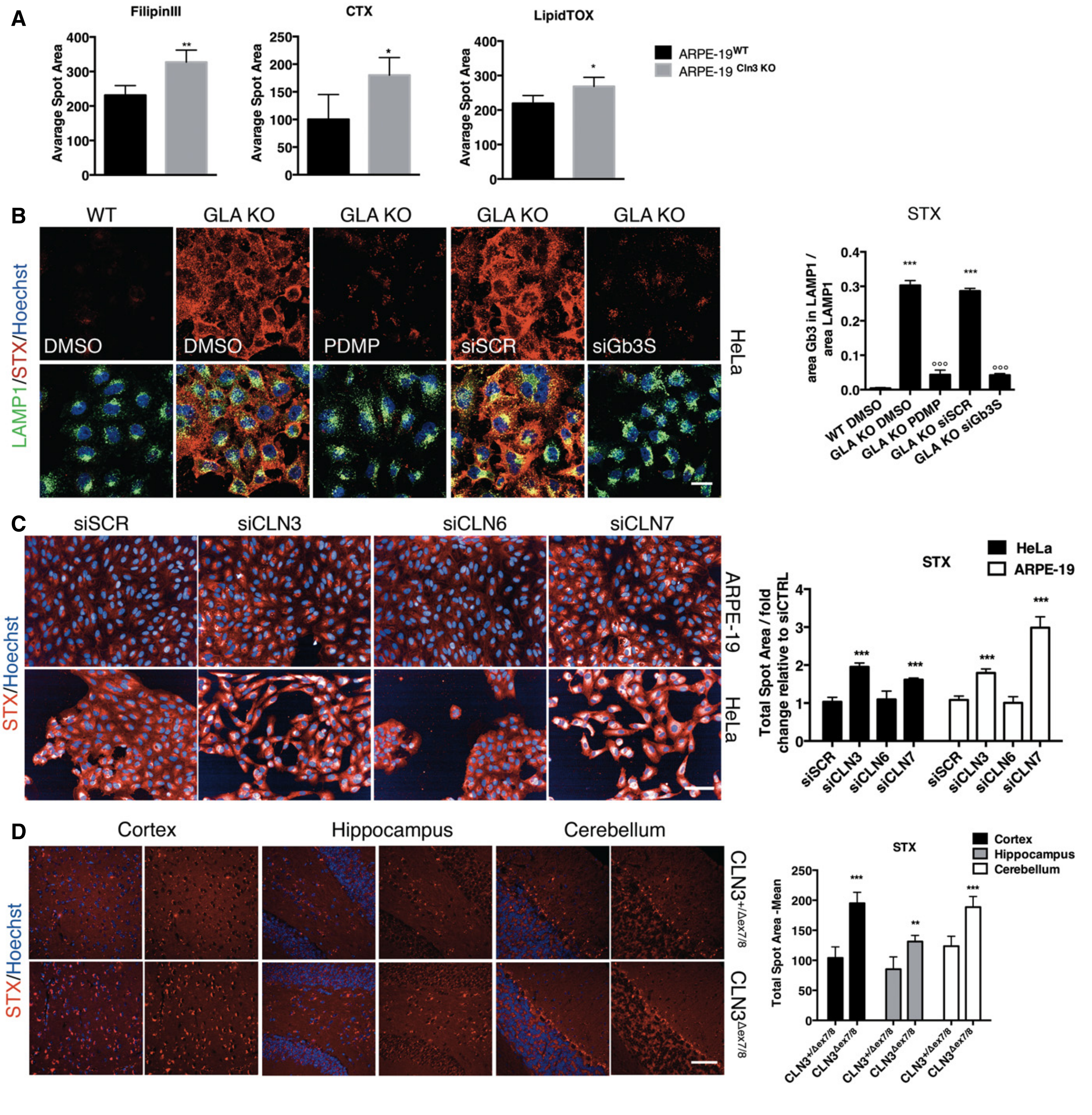

Figure EV1.

 © 2021 The Authors

Figure EV1. Accumulation of lipids and cell-based STX assay validation.

A Quantification of WT and CLN3 KO ARPE-19 cell lines stained using Filipin III (to detect cholesterol) fluorescent-conjugated cholera toxin (to detect GM1) and LipidTox (to detect neutral lipids). Data are presented as mean ± SD. $^{*}P \leq 0.01$, $^{**}P \leq 0.001$ as determined by Student's *t*-test ($n = 3$ biological replicas in duplicate).

B Representative confocal images and quantification of Gb3 accumulation within the lysosome detected by Shiga toxin in WT and GLA KO HeLa cells treated with DMSO (controls) or PDMP or silenced for the Gb3 synthase (siGb3S). (***versus WT, °°° versus DMSO) Data are presented as mean ± SD, $^{\circ\circ\circ}/^{***}P \leq 0.0001$, as determined by ANOVA ($n = 3$ biological replicas in duplicate) Scale bars: 20 µm.

C Representative Opera images and their quantification of STX staining in WT ARPE-19 and HeLa cells silenced for 72 h with siRNAs against a scrambled sequence, CLN3, CLN6, and CLN7. Data are presented as mean ± SD, $^{***}P \leq 0.0001$, as determined by ANOVA ($n = 3$ biological replicas in duplicate) Scale bars: 50 µm.

D Representative confocal images and quantification of Gb3 accumulation, revealed by STX staining, in brain sections from $CLN3^{+/\Delta ex7/8}$ and $CLN3^{\Delta ex7/8}$ mice at 7.5 months of age. Data are presented as mean ± SD, $^{**}P \leq 0.001$, $^{***}P \leq 0.0001$, as determined by ANOVA ($n = 4$ biological replicas) Scale bars: 60 µm.

Source data are available online for this figure.

© 2021 The Authors

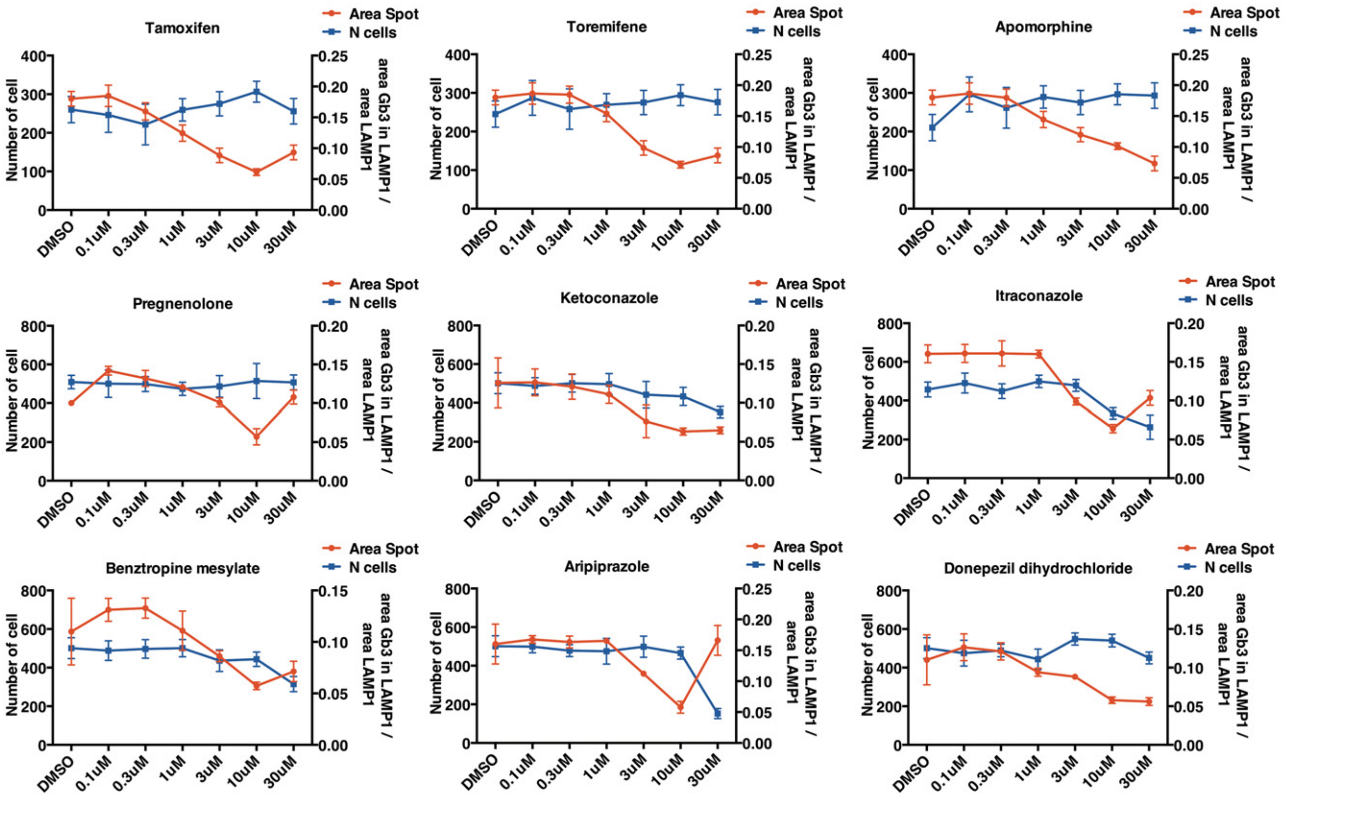

Figure EV2. Dose response.

Dose–response analysis of compound hits derived from the FDA-screening to identify correctors of Gb3 accumulation. Data from both Gb3 accumulation and nuclei count are presented as mean ± SD (n = 4 technical replicas).

Source data are available online for this figure.

Figure EV3. Tamoxifen induces clearance in Batten disease models.

A Representative confocal images and quantification of STX staining within the lysosome of human WT fibroblasts and CLN3 mutants in DMSO or treated with Tamoxifen. (***versus WT, °°° versus DMSO) Data are presented as mean ± SD, °°°/***$P \leq 0.0001$, as determined by ANOVA (n = 3 biological replicas in duplicate) Scale bars: 20 μm.

B Representative confocal images and quantification of STX within the lysosome in ARPE-19 cells silenced with siRNA scramble or against CLN7, in DMSO or treated with Tamoxifen. (***versus siSCR, °°° versus DMSO) Data are presented as mean ± SD, °°°/***$P \leq 0.0001$, as determined by ANOVA (n = 3 biological replicas in duplicate) Scale bars: 20 μm.

C Representative confocal images and quantification of SCMAS accumulation within the lysosomes in wild type and ARPE-19 CLN3 KO cells in DMSO, or treated with Tamoxifen. (***versus WT, °°° versus DMSO) Data are presented as mean ± SD, °°°/***$P \leq 0.0001$, as determined by ANOVA (n = 3 biological replicas in duplicate) Scale bars: 20 μm.

D Representative confocal images and quantification of STX within the lysosome. HeLa cells after acute silencing of CLN3 (siCLN3) in DMSO or treated 48 h with Tamoxifen. (***versus siSCR, °°° versus DMSO). Data are presented as mean ± SD, °°°/***$P \leq 0.0001$, as determined by ANOVA (n = 3 biological replicas in duplicate) Scale bars: 20 μm.

Source data are available online for this figure.

 © 2021 The Authors

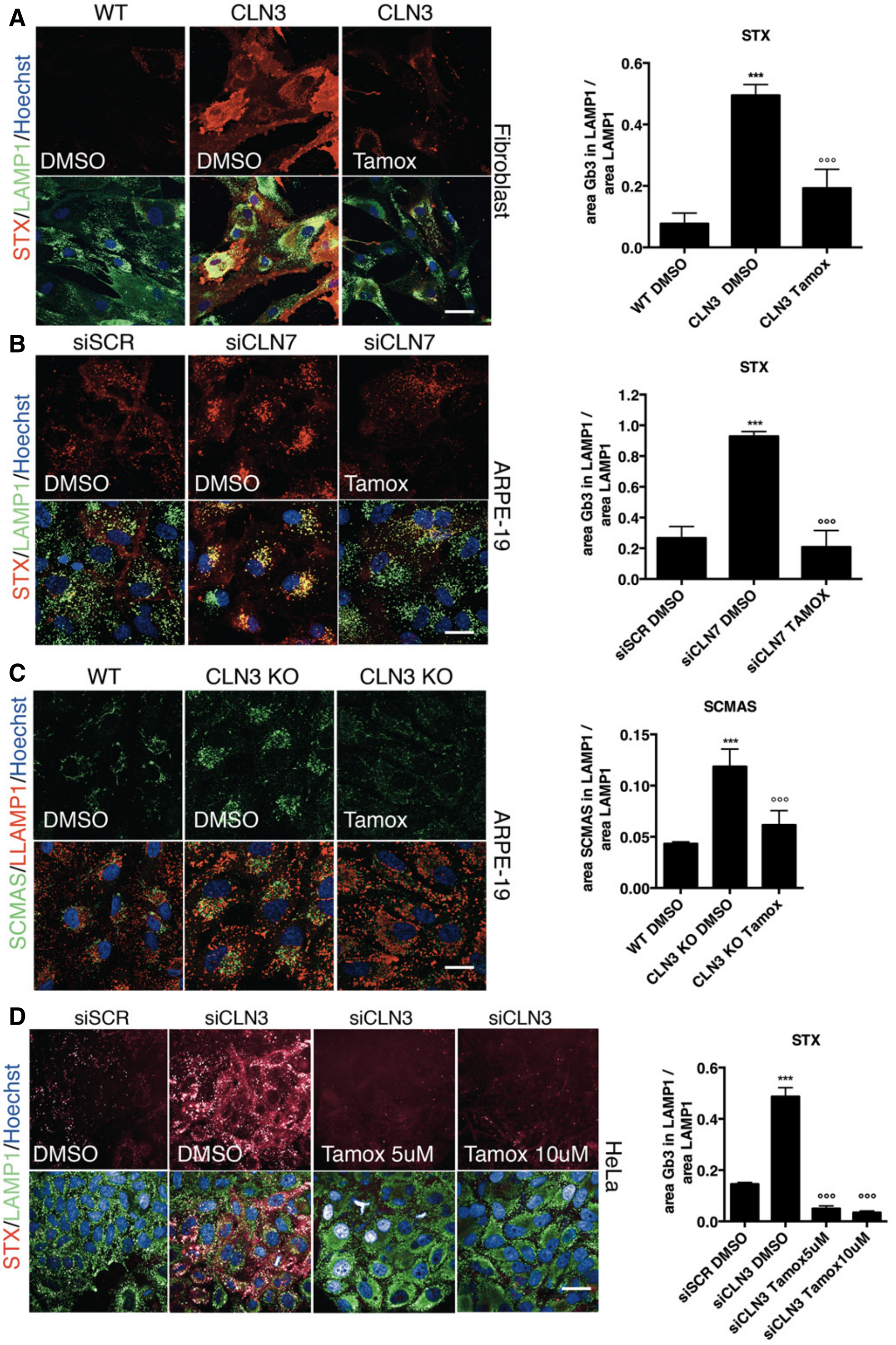
A
WT
CLN3
CLN3
STX/LAMP1/Hoechst
DMSO
DMSO
Tamox
Fibroblast
STX
area Gb3 in LAMP1 / area LAMP1
WT DMSO
CLN3 DMSO
CLN3 Tamox
B
siSCR
siCLN7
siCLN7
STX/LAMP1/Hoechst
DMSO
DMSO
Tamox
ARPE-19
STX
area Gb3 in LAMP1 / area LAMP1
siSCR DMSO
siCLN7 DMSO
siCLN7 TAMOX
C
WT
CLN3 KO
CLN3 KO
SCMAS/LLAMP1/Hoechst
DMSO
DMSO
Tamox
ARPE-19
SCMAS
area SCMAS in LAMP1 / area LAMP1
WT DMSO
CLN3 KO DMSO
CLN3 KO Tamox
D
siSCR
siCLN3
siCLN3
siCLN3
STX/LAMP1/Hoechst
DMSO
DMSO
Tamox 5uM
Tamox 10uM
HeLa
STX
area Gb3 in LAMP1 / area LAMP1
siSCR DMSO
siCLN3 DMSO
siCLN3 Tamox5uM
siCLN3 Tamox10uM

Figure EV3.

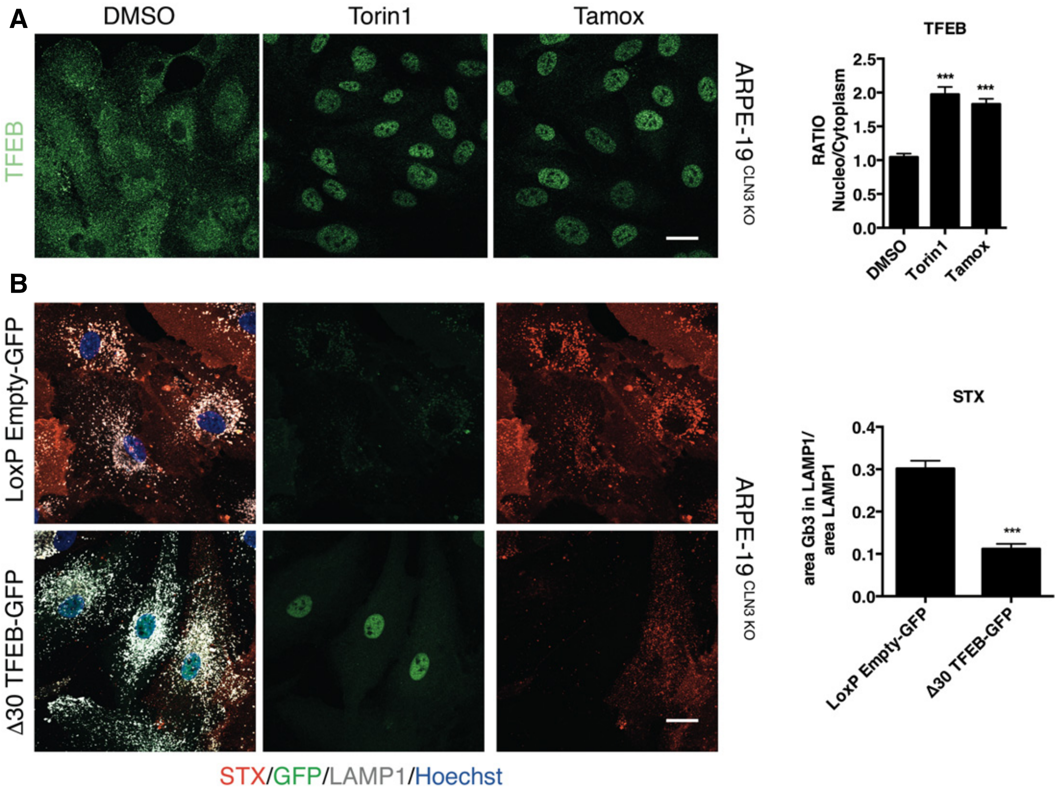

Figure EV4. Tamoxifen induces TFEB activation.

A Representative confocal image and quantification of TFEB localization in ARPE-19 CLN3 KO treated for 3 h with DMSO, Torin1 or Tamoxifen. Data are presented as mean ± SD, ***$P \leq 0.0001$, as determined by ANOVA ($n = 3$ biological replicas in duplicate). Scale bars: 20 μm.

B Representative confocal image and quantification of STX in ARPE-19 CLN3 KO infected with an inducible vector expressing a nuclear-localized mutant form of TFEB. Data are presented as mean ± SD, ***$P \leq 0.0001$, as determined by ANOVA ($n = 3$ biological replicas in duplicate). Scale bars: 20 μm.

Source data are available online for this figure.

 © 2021 The Authors

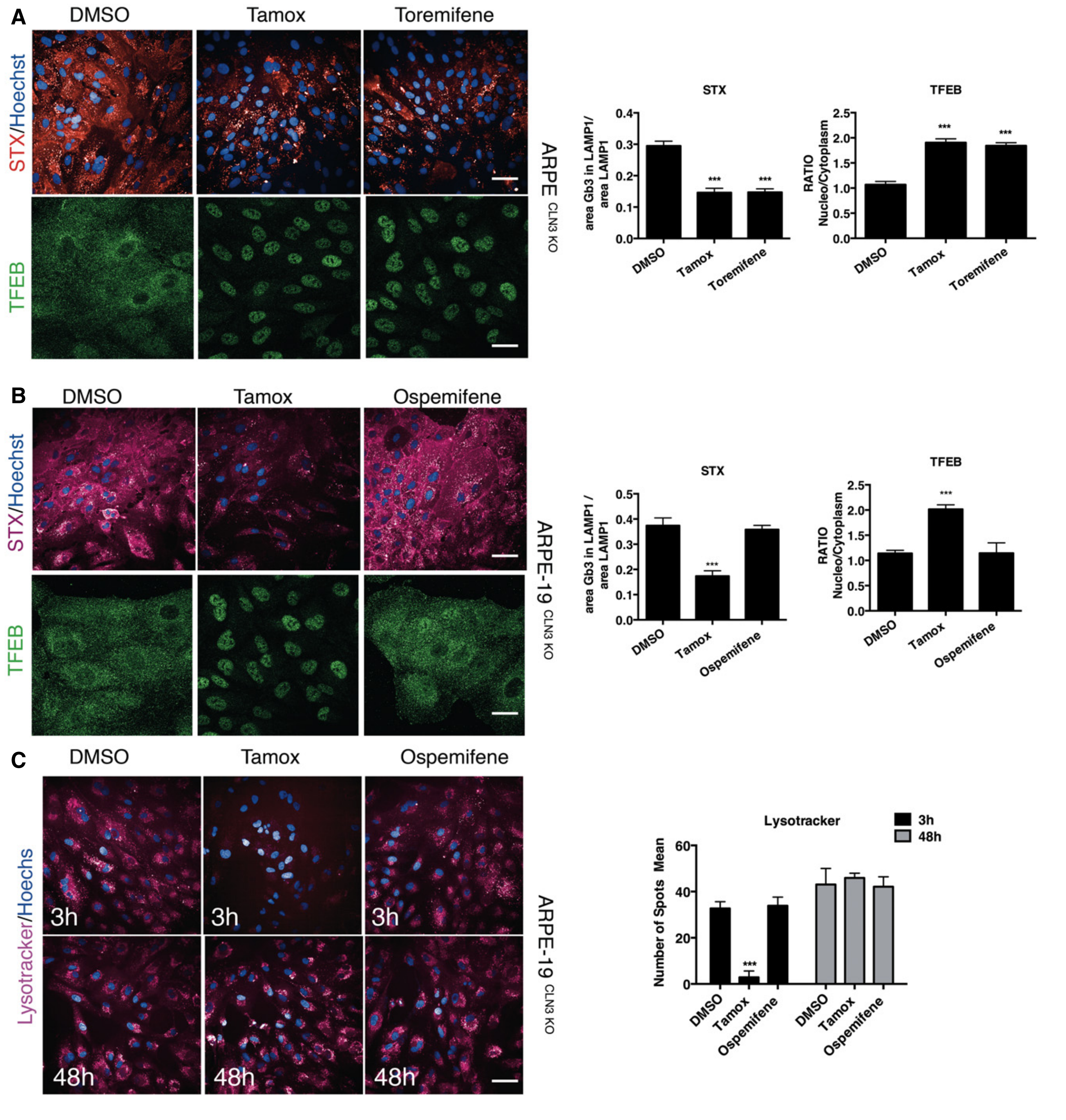

Figure EV5. Effects of ER modulators on Gb3 clearance and TFEB activation.

A Representative Opera and confocal images and quantification of TFEB subcellular localization, and STX accumulation within the lysosome of ARPE-19 CLN3 KO cells treated with DMSO, Tamoxifene or Toremifene for 48 h. Data are presented as mean ± SD, $^{***}P \leq 0.0001$, as determined by ANOVA (n = 3 biological replicas in duplicate). Scale bars: 50 μm top and 20 μm down.

B Representative Opera images and quantification of STX and TFEB localization in ARPE-19 CLN3 KO in DMSO, Tamoxifen or Ospemifene after 48 h. Data are presented as mean ± SD, $^{***}P \leq 0.0001$, as determined by ANOVA (n = 3 biological replicas in duplicate). Scale bars: 50 μm top and 20 μm down.

C Representative Opera images and quantification of Lysotracker-Red staining in ARPE-19 CLN3 KO cells cultivated for 3 and 48 h in the absence (DMSO) or presence of Tamoxifen (Tamox) or Ospemifene. Data are presented as mean ± SD, $^{***}P \leq 0.0001$, as determined by ANOVA (n = 3 biological replicas in duplicate). Scale bars: 20 μm.

Source data are available online for this figure.

© 2021 The Authors
