# Supplementary material for: Repurposing of tamoxifen ameliorates CLN3 and CLN7 disease phenotype
Source: EMBO Mol Med. 2021 Aug 19;13(10):e13742. doi: 10.15252/emmm.202013742 (PMC8495452; doi:10.15252/emmm.202013742)
Supplement: Supplementary file 12 [file EMMM-13-e13742-s007.pdf]

EMBO Molecular Medicine

Repurposing of tamoxifen ameliorates CLN3 and CLN7 disease phenotype

Chiara Soldati, Irene Lopez-Fabuel, Luca Wanderlingh, Marina Garcia-Macia, Jlenia Monfregola, Alessandra Esposito, Gennaro Napolitano, Marta Ferrer, Anna Scotto Rosato, Einar Krogsaeter, Dominik Paquet, Christian Grimm, Sandro Montefusco, Thomas Braulke, Stephan Storch, Sara Mole, Antonella De Matteis, Andrea Ballabio, Julio Sampaio, Tristan McKay, Ludger Johannes, Juan Bolanos, and Diego Medina
DOI: 10.15252/emmm.202013742

Corresponding authors: Diego Medina (medina@tigem.it) , Juan Bolanos (jbolanos@usal.es)

Review Timeline:

Submission Date:	19th Nov 20
Editorial Decision:	24th Dec 20
Revision Received:	11th Jun 21
Editorial Decision:	21st Jul 21
Revision Received:	30th Jul 21
Accepted:	2nd Aug 21

Editor: Zeljko Durdevic

Transaction Report:

(Note: With the exception of the correction of typographical or spelling errors that could be a source of ambiguity, letters and reports are not edited. Depending on transfer agreements, referee reports obtained elsewhere may or may not be included in this compilation. Referee reports are anonymous unless the Referee chooses to sign their reports.)

24th Dec 2020

Dear Prof. Medina,

Thank you for the submission of your manuscript to EMBO Molecular Medicine, and please accept my apologies for the delay in getting back to you. We have received feedback from two of the three reviewers who agreed to evaluate your manuscript. Given that referee #3 will unfortunately not be able to return his/her report in a timely manner, and that both referees #1 and #2 gave similar recommendation, we prefer to make a decision now in order to avoid further delay in the process. Should referee #3 provide a report, we will send it to you, with the understanding that we will not ask for an additional revision. As you will see from the reports below, the referees acknowledge the interest and novelty of the study but also raise serious and partially overlapping concerns that should be addressed in a major revision.

Addressing the reviewers' concerns in full will be necessary for further considering the manuscript in our journal, and acceptance of the manuscript will entail a second round of review. EMBO Molecular Medicine encourages a single round of revision only and therefore, acceptance or rejection of the manuscript will depend on the completeness of your responses included in the next, final version of the manuscript. For this reason, and to save you from any frustrations in the end, I would strongly advise against returning an incomplete revision.

We realize that the current situation is exceptional on the account of the COVID-19/SARS-CoV-2 pandemic. Therefore, please let us know if you need more than six months to revise the manuscript.

I look forward to receiving your revised manuscript.

Yours sincerely,

Zeljko Durdevic
Editor
EMBO Molecular Medicine

***** Reviewer's comments *****

Referee #1 (Comments on Novelty/Model System for Author):

Statistical analysis may need to be revised (i.e., Student's t-test is not appropriate for the data as presented).

NCL/Batten disease is extremely rare in the general population. In addition, the results of this study

may only be relevant to 2 of the 13 different subtypes of NCL/Batten disease (CLN3 and CLN7 disease).

Paper would benefit from examining Gb3 accumulation and correction in patient derived cells lines (e.g., neurons generated from iPS cells derived from patient fibroblasts).

Referee #1 (Remarks for Author):

This is a nice report. However, the conclusions would be strengthened by including observations from at least one patient-derived cell model (e.g., neurons derived from iPS cells generated from patient fibroblasts).

Abstract: "Altogether, these data strongly suggest that tamoxifen is a suitable drug for the treatment of Batten disease" - The authors should temper their optimism by stating that tamoxifen MAY be a suitable drug for treating Batten disease. This should be done elsewhere in the manuscript as well.

Introduction: Please clarify what is meant by "the most common of the rare neurodegenerative disorders in children". Are all childhood forms of neurodegeneration rare?

Introduction: Work in a variety of model systems (e.g., slime mold, sheep, HeLa cells, etc) has shown that CLN5 also localizes extracellularly. Please correct.

Introduction/Results/Discussion: The authors should provide some rationale as to why they focused on CLN3 and CLN7 models and not others. The results of this study suggest that tamoxifen might be an appropriate therapy for CLN3 and CLN7 disease, but it is premature to suggest that it can correct defects in all subtypes of NCL (i.e., since Gb3 accumulation and reduction was not assessed for all NCL genes). In fact, the authors stated that Gb3 did not accumulate in cells devoid of CLN6. Based on these observations, perhaps the findings can be explained by CLN3 and CLN7 both being transmembrane proteins? If yes, could the authors speculate in the Discussion as to why? Since the authors did not examine other NCL proteins that belong to different classes (e.g., enzymes), they should consider altering the title and any relevant text in the manuscript to specify which subtypes of NCL may be targeted by tamoxifen.

Results: "The PDMP inhibitor" - For clarity, this should be corrected to "the glucosylceramide synthase inhibitor PDMP".

Results: "Also, we discard compounds reducing the cell viability to 40% compared to DMSO-treated controls" - The authors should mention why the 40% threshold is important. Is this required for a compound to be considered for therapy?

Results: The authors state "Tamoxifen resulted in the most effective drug reducing Gb3 accumulation with an EC50 of 0.75 µM". However later in the Results, 10 uM is used. Can the authors please clarify why a higher concentration of tamoxifen was used for certain assays. Also, is 10 uM tamoxifen suitable for therapy? This should be mentioned in the Discussion.

Discussion: The authors should comment on how tamoxifen might be administered to a patient in a clinical setting and whether or not the results they present here may be limited to certain cell types.

Fig.1 caption: The y-axis label does not make it clear what's being measured. Could the authors

please clarify? This may also need to be done for the other figures.

The authors may need to adjust their statistical approach. I do not think the Student's t-test is an appropriate test for any of plots.

Grammatical errors that require correction

Title: Consider replacing "repositioning" with "repurposing".

Abstract: Specify that CLN3 and CLN7 diseases are subtypes of NCL.

Abstract: "TFEB activation by tamoxifen is trigger by a..." - Replace "trigger" with "triggered". Also, remove "a" from this sentence.

Introduction: "TFEB activation by tamoxifen is trigger by a..." - Replace "trigger" with "triggered". Also, remove "a" from this sentence.

Results: Remove "the" from the first sentence.

Results: "While seeking for glycosphingolipid accumulation..." - This is a run-on sentence. Please consider fragmenting the sentence into two.

Results: "Thus, we develop a cell-based assay to identify FDA..." - Replace "develop" with "developed". Many sentences in this section are currently presented in present tense. However, all statements in the Results section should be in past tense. Also, "Thus we develop a cell-based assay to identify FDA..." is a very long sentence. Consider fragmenting into two sentences.

Discussion: "Additionally, we observe that tamoxifen induces TFEB nuclear translocation by specifically impairs mTORC1-mediated phosphorylation of TFEB..." - Replace "impairs" with "impairing".

Referee #2 (Remarks for Author):

Comments to the editor and authors

Neuronal ceroid lipofuscinosis (also called Batten disease) is a devastating family of early-onset neurodegenerative diseases whose pathology is still to be determined. Because of the lack of molecular understanding of their pathophysiology, no cure is available. In this manuscript, Soldati et al. identifies a potential biomarker (Gb3 accumulation) that is shared between two forms of Batten disease, NCL3 and NCL7. Using high content imaging screens, they identify potential drugs that can reverse Gb3 accumulation. They further describe a potential positive effect of the identified drug, Tamoxifen, on several hallmarks of Batten disease in vivo.

We believe that the experiments are mostly well-performed and the conclusions are in general supported by these experiments, however, we have several concerns that we believe the authors should address to improve their manuscript and strengthen their conclusions.

Concerns and suggestions:

1) The manuscript (main text, methods, legends and figure labeling) will benefit from professional editing. We have encountered several grammar and spelling mistakes throughout the manuscript.

2) The authors don't describe their approach to identify Gb3 as a biomarker. The authors stated that while seeking to identify what glycosphingolipids accumulate in CLN3 KO cells they found Gb3. Were there other lipids in the list or they only looked at Gb3? This can help provide a better understanding of why Gb3 is accumulating.

3) Throughout the manuscript, the authors focus on the lysosomal accumulation of Gb3, however a careful look at their IF images clearly indicates that Gb3 equally accumulates in other membranes. We believe that the authors should refrain from giving the impression that Gb3 accumulation is strictly lysosomal by using a more accurate description

4) (4 and 5 are major concerns) While Shiga toxin is a good approach to detect Gb3, these toxins are never specific. The authors tried to show the specificity of this toxin using genetic approaches, however, to provide definitive evidence that Gb3 is indeed accumulating (which is the major biological finding in the paper) and not some other metabolic intermediate or lipid species, the authors should provide mass spectrometry (MS) data. MS-based approaches to detect and quantify Gb3 are readily available and commonly used as LysoGb3 is a major biomarker for Fabry disease. This structural data will also improve the manuscript by identifying the exact species that accumulates.

5) It is concerning to us that one of the major approaches that the authors used to show the specificity of the toxin to Gb3 is a stain with anti CD77 antibody. The catalogue number that is mentioned in the methods (19795) couldn't be found on abcam website.
Also, the authors should correct both the text and legend when they talk about Gb3 in that context and describe it as Gb3 protein. We believe it is a typo as Gb3 (CD77) is a lipid.

7) The authors didn't investigate if the identified compounds that affect the levels of Gb3 share any structural or chemical properties. This can be useful to have in the manuscript.

8) The authors didn't describe the hook effect seen in Fig S3, that shows that the highest Tamoxifen dose has less effect on Gb3 levels. This can be very important for any preclinical application.

9) The authors should provide an expression analysis for the Estrogen Receptor in HeLa and U2-OS cells. There is known heterogeneity between cancer cells and they cannot depend on available literature to rule out its involvement without testing it in the same cells they used in their experiments.

10) Images in Fig S5B are low quality and they are over-exposed. No decrease in the cytosolic TFEB signal can be observed upon translocation to the nucleus. These experiments should be repeated. Same is for S6B, where TFEB is still observed in the nucleus upon Ospemifene treatment although the authors claim it is not, which is another major concern. We believe the author should provide higher quality images to have a more conclusive evidence.

11) The authors investigate the mechanism of TFEB regulation by Tamoxifen and provide evidence that it involves RagC. However, the authors don't link this to Gb3 clearance. The authors should perform same RagC over expression experiments and see its effects on Gb3 clearance by

tamoxifen, otherwise these data are irrelevant to the current manuscript.

12) Some figures are not clearly labeled. Fig. 4D is one of such figures.

13) We are really concerned about the fact that the authors did most of their cell-based work in CLN3 KO cells but only performed the functional in vivo work in CLN7 models. The authors claimed that the phenotype in CLN3 KO mouse is subtle, however, there have been many neurological hallmarks that are established in this model. We believe that the authors should provide data in CLN3 KO model.

Rebuttal_ Soldati et al. EMM-2020-13742
In order to facilitate the revision, we have underlined added text in order to better track major changes in the manuscript.

Referee #1 (Comments on Novelty/Model System for Author):

Statistical analysis may need to be revised (i.e., Student's t-test is not appropriate for the data as presented).

We have followed the suggestion of Referee #1 and revised all the data with our Bioinformatics Core here at TIGEM. When it has been suggested and following their professional advice, we have re-analyzed raw data to perform the appropriate tests. All the statistical information has been added to Methods and legends.

NCL/Batten disease is extremely rare in the general population. In addition, the results of this study may only be relevant to 2 of the 13 different subtypes of NCL/Batten disease (CLN3 and CLN7 disease).

The incidence rates of the entire group of 13 genetically different NCL diseases varying between 1:14,000-1:67,000 (1; the references are given at the end of this rebuttal letter). CLN3 disease (MIM # 204200) represents the worldwide most common form of NCL. Thus, our work includes the most prevalent BD worldwide, juvenile CLN3 disease, and one of the most prevalent BD in southern and Mediterranean Europe, CLN7 disease. Because they are very rare, we think it is very important that someone studies these diseases. Repurposing drugs that may slow down the course of the CLN3 and/or CLN7 deficiency would be a great step.

Paper would benefit from examining Gb3 accumulation and correction in patient derived cells lines (e.g., neurons generated from iPS cells derived from patient fibroblasts).

Although it has been very difficult to find collaborators with such models available during the pandemic, we have achieved this request. Now, we have provided evidence of Gb3 accumulation and clearance upon tamoxifen treatment in Neuronal Precursors Cells (NPCs) derived from a CLN7 patient iPSCs (New Figure 3E).

Referee #1 (Remarks for Author):

This is a nice report. However, the conclusions would be strengthened by including observations from at least one patient-derived cell model (e.g., neurons derived from iPS cells generated from patient fibroblasts).

Although it has been very difficult to find collaborators with such models available and during the pandemic, we have achieved this request. Now, we have provided evidence of GB3 accumulation and clearance by tamoxifen in Neuronal Progenitor Cells (NPCs) derived

from CLN7 patients (see methods section). These results have been included in the main text and Figure 3E.

Abstract: "Altogether, these data strongly suggest that tamoxifen is a suitable drug for the treatment of Batten disease" - The authors should temper their optimism by stating that tamoxifen MAY be a suitable drug for treating Batten disease. This should be done elsewhere in the manuscript as well.

As suggested, we have tempered our optimism elsewhere in the manuscript

Introduction: Please clarify what is meant by "the most common of the rare neurodegenerative disorders in children". Are all childhood forms of neurodegeneration rare?

Thanks for the comment, and I apologize for the lack of clarity. Neuronal ceroid lipofuscinosis (NCLs) is a group of inherited neurodegenerative lysosomal storage diseases that together represent the most common cause of dementia in children (2). We have added this sentence and the reference in the manuscript.
As we said before, the incidence rates of the entire group of 13 genetically different NCL diseases varying between 1:14,000-1:67,000 (1), and CLN3 disease (MIM # 204200) represents the world-wide most common form of NCL. CLN7, instead, is one of the most prevalent BD in southern and Mediterranean Europe.

Introduction: Work in a variety of model systems (e.g., slime mold, sheep, HeLa cells, etc) has shown that CLN5 also localizes extracellularly. Please correct.

Corrected and added a reference

Introduction/Results/Discussion: The authors should provide some rationale as to why they focused on CLN3 and CLN7 models and not others. The results of this study suggest that tamoxifen might be an appropriate therapy for CLN3 and CLN7 disease, but it is premature to suggest that it can correct defects in all subtypes of NCL (i.e., since Gb3 accumulation and reduction was not assessed for all NCL genes). In fact, the authors stated that Gb3 did not accumulate in cells devoid of CLN6. Based on these observations, perhaps the findings can be explained by CLN3 and CLN7 both being transmembrane proteins? If yes, could the authors speculate in the Discussion as to why? Since the authors did not examine other NCL proteins that belong to different classes (e.g., enzymes), they should consider altering the title and any relevant text in the manuscript to specify which subtypes of NCL may be targeted by tamoxifen.

Thanks for the comment. We followed the strategy of combining a phenotypic cell-based high content imaging assay with the repurposing of FDA-approved drugs to identify correctors of two subtypes of Batten disease. We have focused on CLN3 and CLN7 because these are polytopic lysosomal membrane proteins which are related to the most prevalent

world-wide juvenile CLN3 disease, and the most common CLN7 disease in southern and mediterranean Europe, respectively. In contrast to the polytopic CLN6 (used as negative control) localized in the endoplasmic reticulum and cis/Golgi, CLN3 and CLN7 are most likely directly involved in the egress or sensing of lysosomal degradation products/metabolites. The referee is right, and like her/him we think that common findings in CLN3 and CLN7 might be related to their localization and transmembrane nature. We have added the rationale in the introduction and some text in the discussion. Also, we changed the title of the manuscript.

Results: "The PDMP inhibitor" - For clarity, this should be corrected to "the glucosylceramide synthase inhibitor PDMP".

We have added the definition.

Results: "Also, we discard compounds reducing the cell viability to 40% compared to DMSO-treated controls" - The authors should mention why the 40% threshold is important. Is this required for a compound to be considered for therapy?

We set up a threshold of cell vitality to prevent false positives caused by either cytotoxicity or cytostasis in the primary screening. From my experience at Eli Lilly&Co, the cellular toxicity observed in vitro cannot predict at 100% the toxicity in vivo, but can help us to focus on the best compounds for the subsequent administration in vivo.

Results: The authors state "Tamoxifen resulted in the most effective drug reducing Gb3 accumulation with an EC50 of 0.75 µM". However later in the Results, 10 uM is used. Can the authors please clarify why a higher concentration of tamoxifen was used for certain assays. Also, is 10 uM tamoxifen suitable for therapy? This should be mentioned in the Discussion.

Usually, for drug-receptor kinetics (following Michaelis-Menten) the substrate saturation is reached using a 25-100-fold excess of the KM-/Ki-value, and therefore we used doses in the lower end of this range. We have also presented data in this manuscript using 5uM tamoxifen (see Fig. 4A, Fig.EV3D). For patients with breast cancer, the recommended daily dose of tamoxifen is 20 to 40 mg (during 5-10 years) (https://www.accessdata.fda.gov/drugsatfda_docs/label/2018/021807s005lbl.pdf) (3). We have treated mice with 40 mg/kg which is in line with concentrations used to test efficacy in mouse models (https://monographs.iarc.fr/wp-content/uploads/2018/06/mono100A-13.pdf). Our aim in this manuscript is to provide proof of principle in vivo, and therefore specific dosing studies would be necessary for a new medical use of this compound.

Discussion: The authors should comment on how tamoxifen might be administered to a patient in a clinical setting and whether or not the results they present here may be limited to certain cell types.

For its current medical indication, tamoxifen is administered orally. Also, tamoxifen crosses the blood-brain barrier and has shown neuroprotective activity in rat and dog models of ischemia and stroke, respectively (4-6). Together with our in vivo data, showing tamoxifen activity reverting neurological hallmarks of CLN7 disease, we propose this FDA drug for the treatment of two subtypes of BD. We have included some of these comments in the discussion

Fig.1 caption: The y-axis label does not make it clear what's being measured. Could the authors please clarify? This may also need to be done for the other figures.

Accumulation of Gb3 is measured by analyzing the Area of Gb3 spots (detected with STX) within the Lysosome spots (detected by Lamp1) normalized for the total area of the lysosome spots (detected by Lamp1) the labeling "Area Gb3 in Lamp1 Cytoplasm/Lamp1 area in cytoplasm" has been changed with "Area Gb3 in Lamp1/ area Lamp1". Representative changes have been made in the legends and graphs

The authors may need to adjust their statistical approach. I do not think the Student's t-test is an appropriate test for any of plots.

We have followed Referee #1 suggestion and discuss all the data with our Bioinformatics Core here at TIGEM. We have re-analyzed raw data and performed the appropriate tests following their professional recommendations.

Grammatical errors that require correction

Title: Consider replacing "repositioning" with "repurposing".
DONE

Abstract: Specify that CLN3 and CLN7 diseases are subtypes of NCL. *DONE*

Abstract: "TFEB activation by tamoxifen is trigger by a..." - Replace "trigger" with "triggered". Also, remove "a" from this sentence.
DONE

Introduction: "TFEB activation by tamoxifen is trigger by a..." - Replace "trigger" with "triggered". Also, remove "a" from this sentence.
DONE

Results: Remove "the" from the first sentence.
DONE

Results: "While seeking for glycosphingolipid accumulation..." - This is a run-on sentence. Please consider fragmenting the sentence into two.

We have changed this part following the suggestion of reviewer 2

Results: "Thus, we develop a cell-based assay to identify FDA..." - Replace "develop" with "developed". Many sentences in this section are currently presented in present tense. However, all statements in the Results section should be in past tense. Also, "Thus we develop a cell-based assay to identify FDA..." is a very long sentence. Consider fragmenting into two sentences.
DONE

Discussion: "Additionally, we observe that tamoxifen induces TFEB nuclear translocation by specifically impairs mTORC1-mediated phosphorylation of TFEB..." - Replace "impairs" with "impairing".
DONE

Referee #2 (Remarks for Author):

Comments to the editor and authors

Neuronal ceroid lipofuscinosis (also called Batten disease) is a devastating family of early-onset neurodegenerative diseases whose pathology is still to be determined. Because of the lack of molecular understanding of their pathophysiology, no cure is available. In this manuscript, Soldati et al. identifies a potential biomarker (Gb3 accumulation) that is shared between two forms of Batten disease, NCL3 and NCL7. Using high content imaging screens, they identify potential drugs that can reverse Gb3 accumulation. They further describe a potential positive effect of the identified drug, Tamoxifen, on several hallmarks of Batten disease in vivo.

We believe that the experiments are mostly well-performed and the conclusions are in general supported by these experiments, however, we have several concerns that we believe the authors should address to improve their manuscript and strengthen their conclusions.

Concerns and suggestions:

1) The manuscript (main text, methods, legends and figure labeling) will benefit from professional editing. We have encountered several grammar and spelling mistakes throughout the manuscript.

We have tried to address all mistakes, also we have sent the manuscript to our English native collaborators (Sara Mole, Tris McKay) that gently edited the manuscript.

2) The authors don't describe their approach to identify Gb3 as a biomarker. The authors stated that while seeking to identify what glycosphingolipids accumulate in CLN3 KO cells they found Gb3. Were there other lipids in the list or they only looked at Gb3? This can help provide a better understanding of why Gb3 is accumulating.

To determine the best cell-based high content imaging assay for the screening of BD cells, we performed cell-based phenotyping assays to seek a variety of lipid accumulation in ARPE-19 CLN3 KO cells. Initially, this part was eliminated from the original draft for the sake of simplicity. Now, upon the reviewer's suggestion, we have added these results (new Figure 1A).

3) Throughout the manuscript, the authors focus on the lysosomal accumulation of Gb3, however a careful look at their IF images clearly indicates that Gb3 equally accumulates in other membranes. We believe that the authors should refrain from giving the impression that Gb3 accumulation is strictly lysosomal by using a more accurate description

Thanks for the comment. The referee is right. Although we are interested in promoting the clearance of pathological accumulation of lysosomal Gb3, it is obvious that Gb3 is also present in other membranes. Thus, we refrain from the text corresponding to the description of Fig1B.

4) (4 and 5 are major concerns) While Shiga toxin is a good approach to detect Gb3, these toxins are never specific. The authors tried to show the specificity of this toxin using genetic approaches, however, to provide definitive evidence that Gb3 is indeed accumulating (which is the major biological finding in the paper) and not some other metabolic intermediate or lipid species, the authors should provide mass spectrometry (MS) data. MS-based approaches to detect and quantify Gb3 are readily available and commonly used as LysoGb3 is a major biomarker for Fabry disease. This structural data will also improve the manuscript by identifying the exact species that accumulates.

We have performed lipidomics analysis of Gb3 accumulation in ARPE-19 depleted of CLN3 KO, newly generated ARPE-19-CLN7-KO cell line, and freshly isolated neurons derived from the forebrain of CLN7 mice. As expected from the highly selective affinity of the Shiga toxin reagent, we found an accumulation of Gb3 compared to WT counterparts (New Figure 1D). We have found other glycosphingolipids accumulated that are precursors of Gb3 and GM3 synthesis, confirming that CLN3 and CLN7 are involved in the glycosphingolipid pathway. We have added these results in the text and also discussed them.

It is concerning to us that one of the major approaches that the authors used to show the specificity of the toxin to Gb3 is a stain with anti CD77 antibody. The catalogue number that is mentioned in the methods (19795) couldn't be found on abcam website.
Also, the authors should correct both the text and legend when they talk about Gb3 in that context and describe it as Gb3 protein. We believe it is a typo as Gb3 (CD77) is a lipid.

CD77 has been widely used to detect endogenous levels of Gb3 (here some very recent references using this antibody, (7-10)). Ab19795 datasheet can be found on the ABCAM website (https://www.abcam.com/cd77-antibody-38-13-ab19795.html), but unfortunately, it is no longer available.
We also apologize for the typo. Following the suggestion of the reviewer, we have now demonstrated by lipidomic analysis that endogenous Gb3 is accumulated in these disease

models (Figure 1D), and therefore we have eliminated the redundant CD77 data from the manuscript.

7) The authors didn't investigate if the identified compounds that affect the levels of Gb3 share any structural or chemical properties. This can be useful to have in the manuscript.

We have identified 9 compound hits. Two compounds belong to the stilbenoid class of drugs and are selective estrogen receptor modulators (Tamoxifen and Toremifene), one alkaloid (apomorphine), three phenylpiperazines (itraconazole, ketoconazole, aripiprazole), a derivative of cholesterol (pregnenolone), a diphenylmethane (Benztropine), and an n-benzylpiperidine (donepezil). We have selected the compound class that shown the best potency in our assays. Interestingly, the LogP and pKa properties of five out of the nine hits correspond to drugs with potential lysosomotropic properties (LogP>2 and pKa 6-11), supporting our hypothesis that this property may represent at least one of the mechanisms of action of these drugs. We have added the structural analysis and comment on these observations in the text.

8) The authors didn't describe the hook effect seen in Fig S3, that shows that the highest Tamoxifen dose has less effect on Gb3 levels. This can be very important for any preclinical application.

We have provided dose-response data for all the compound hits identified that include Gb3 clearance and cellular viability. Looking at the plot from the tamoxifen dose-response, the average of nuclei is slightly decreased at 30 uM. Indeed, the potency clearing Gb3 is slightly reduced at the same highest concentration Thus, these results may indicate potential cytotoxicity or cytostasis starting at the highest dose. We have included this comment.

9) The authors should provide an expression analysis for the Estrogen Receptor in HeLa and U2-OS cells. There is known heterogeneity between cancer cells and they cannot depend on available literature to rule out its involvement without testing it in the same cells they used in their experiments.

We have performed the qPCR to confirm the lack of ERs in both HeLa and U2-OS cells. The analysis is now described in the text and shown in Appendix Fig. S6B.

10) Images in Fig S5B are low quality and they are over-exposed. No decrease in the cytosolic TFEB signal can be observed upon translocation to the nucleus. These experiments should be repeated. Same is for S6B, where TFEB is still observed in the nucleus upon Ospemifene treatment although the authors claim it is not, which is another major concern. We believe the author should provide higher quality images to have a more conclusive evidence.

To improve image quality, more experiments have been repeated and new images added

11) The authors investigate the mechanism of TFEB regulation by Tamoxifen and provide evidence that it involves RagC. However, the authors don't link this to Gb3 clearance. The authors should perform the same RagC over expression experiments and see its effects on Gb3 clearance by tamoxifen, otherwise these data are irrelevant to the current manuscript.

As suggested, we have tested the effects of blocking tamoxifen-mediated TFEB nuclear translocation, by using the overexpression of HA-RagC, on Gb3 clearance. As expected, we found a block of Gb3 clearance in cells transfected with HA-RagC and treated with tamoxifen compared to cells transfected with HA-empty vectors and treated with tamoxifen. Additionally, we have infected CLN3 KO cells with an inducible nuclear form of TFEB. Using this approach, we further confirm that TFEB expression induces Gb3 clearance. These results are now in Fig. 5D and Fig. EV4B, and all are discussed in the text.

12) Some figures are not clearly labeled. Fig. 4D is one of such figures.

We have re-edited the figures

13) We are really concerned about the fact that the authors did most of their cell-based work in CLN3 KO cells but only performed the functional in vivo work in CLN7 models. The authors claimed that the phenotype in CLN3 KO mouse is subtle, however, there have been many neurological hallmarks that are established in this model. We believe that the authors should provide data in the CLN3 KO model.

We already present a proof of principle in vivo, using the CLN7 mouse model which includes biochemical and motor analysis upon tamoxifen treatment. In the BD field it is well-known that CLN7 mice better resemble the human phenotype compared with the slower and more subtle progression of the CNS phenotype in the CLN3 mouse model. Therefore upon serious consideration of these differences and discuss the best strategy with pediatricians following LSD and BD patients (i.e. Dra Angela Schulz, Dr. Giancarlo Parenti, etc) we decided to test tamoxifen efficacy in CLN7 mice.
We have received these comments during the second lock-down in most European countries (24-12-2020). During this horrible period, our collaborators involved in the animal studies at the University of Salamanca (Spain) were forced to sacrifice the vast majority of the mice and stop all breedings, including the CLN3 mice. We discussed the 12th of January 2021 these issues with the editor and Dr. Durdevic and he understood our strong arguments and our impossibility to start a new in vivo project that will take 12-13 months. Instead, we have addressed all the rest of the concerns from both reviewers. These points include the most important points raised by reviewer2 (points 4-5). All the new data have improved the manuscript further confirmed both endogenous accumulation of Gb3 in CLN3 and CLN7 disease models and the efficacy of tamoxifen ameliorating phenotypic hallmarks of BD in vitro and in vivo (including NPC derived from CLN7 patient iPSCs).

References

1. M. Haltia, H. H. Goebel, The neuronal ceroid-lipofuscinoses: A historical introduction, Biochimica et Biophysica Acta (BBA) - Molecular Basis of Disease 1832, 1795–1800 (2013).

2. N. Specchio, A. Ferretti, M. Trivisano, N. Pietrafusa, C. Pepi, C. Calabrese, S. Livadiotti, A. Simonetti, P. Rossi, P. Curatolo, F. Vigevano, Neuronal Ceroid Lipofuscinosis: Potential for Targeted Therapy, Drugs 81, 101–123 (2021).

3. R. C. Bergan, E. Reed, C. E. Myers, D. Headlee, O. Brawley, H. K. Cho, W. D. Figg, A. Tompkins, W. M. Linehan, D. Kohler, S. M. Steinberg, M. V. Blagosklonny, A Phase II study of high-dose tamoxifen in patients with hormone-refractory prostate cancer, Clin Cancer Res 5, 2366–2373 (1999).

4. A. S. Boulos, E. M. Deshaies, J. C. Dalfino, P. J. Feustel, A. J. Popp, D. Drazin, Tamoxifen as an effective neuroprotectant in an endovascular canine model of stroke, J Neurosurg 114, 1117–1126 (2011).

5. H. K. Kimelberg, Tamoxifen as a powerful neuroprotectant in experimental stroke and implications for human stroke therapy, Recent Pat CNS Drug Discov 3, 104–108 (2008).

6. H. K. Kimelberg, Y. Jin, C. Charniga, P. J. Feustel, Neuroprotective activity of tamoxifen in permanent focal ischemia, J Neurosurg 99, 138–142 (2003).

7. F. Braun, L. Blomberg, S. Brodesser, M. C. Liebau, B. Schermer, T. Benzing, C. E. Kurschat, Enzyme Replacement Therapy Clears Gb3 Deposits from a Podocyte Cell Culture Model of Fabry Disease but Fails to Restore Altered Cellular Signaling, Cell Physiol Biochem 52, 1139–1150 (2019).

8. G. G. Slaats, F. Braun, M. Hoehne, L. E. Frech, L. Blomberg, T. Benzing, B. Schermer, M. M. Rinschen, C. E. Kurschat, Urine-derived cells: a promising diagnostic tool in Fabry disease patients, Sci Rep 8, 11042 (2018).

9. H.-Y. Song, H.-C. Chiang, W.-L. Tseng, P. Wu, C.-S. Chien, H.-B. Leu, Y.-P. Yang, M.-L. Wang, Y.-J. Jong, C.-H. Chen, W.-C. Yu, S.-H. Chiou, Using CRISPR/Cas9-Mediated GLA Gene Knockout as an In Vitro Drug Screening Model for Fabry Disease, Int J Mol Sci 17 (2016), doi:10.3390/ijms17122089.

10. Y. J. Jeon, N. Jung, J.-W. Park, H.-Y. Park, S.-C. Jung, Epithelial-Mesenchymal Transition in Kidney Tubular Epithelial Cells Induced by Globotriaosylsphingosine and Globotriaosylceramide, PLoS One 10, e0136442 (2015).

21st Jul 2021

Dear Prof. Medina,

Thank you for the submission of your revised manuscript to EMBO Molecular Medicine. I am pleased to inform you that we will be able to accept your manuscript pending the following final amendments:

1) Please address all the points raised by the referees.
2) Please revise your article for grammar and syntax (i.e. by an English native speaker).
3) In the main manuscript file, please do the following:
- Please check again the track changes suggested by our data editors by working from the attached document.
- Add up to 5 keywords.
- Make sure that all special characters display well.
- Add contributions for Marta Guevara-Ferrer, Maria Antonietta De Matteis, Tristan McKay.
- In M&M, Include a statement that informed consent was obtained from all human subjects and that the experiments conformed to the WMA Declaration of Helsinki and to the principles set out in the Department of Health and Human Services Belmont Report.
- In M&M, statistical paragraph should reflect all information that you have filled in the Authors Checklist, especially regarding randomization, blinding, replication etc.
- Rename "Bibliography" to "References".
4) Appendix: Please rename Table 1 and Table 2 to Table S1 and Table S2 and correct their callouts in the text. Please add Table S1 and S2 to the table of content.
5) Funding: Please make sure that information about all sources of funding are complete in both our submission system and in the manuscript.
6) Source data: Please upload 1 file per figure for the main figures and 1 zipped file for EV and 1 zipped file for Appendix Figures.
7) The Paper Explained: Please provide "The Paper Explained" and add it to the main manuscript text. Please check "Author Guidelines" for more information.
https://www.embopress.org/page/journal/17574684/authorguide#researcharticleguide
8) Synopsis:
- Synopsis text: Alongside the bullet points synopsis should have a short stand first (maximum of 300 characters, including space) that summarise the paper. Please use the passive voice.
- Please check your synopsis text and image, revise them if necessary and submit the final versions with your revised manuscript. Please be aware that in the proof stage minor corrections only are allowed (e.g., typos). Please submit synopsis text as a separate .doc file.
9) For more information: There is space at the end of each article to list relevant web links for further consultation by our readers. Could you identify some relevant ones and provide such information as well? Some examples are patient associations, relevant databases, OMIM/proteins/genes links, author's websites, etc...
10) As part of the EMBO Publications transparent editorial process initiative (see our Editorial at http://embomolmed.embopress.org/content/2/9/329), EMBO Molecular Medicine will publish online a Review Process File (RPF) to accompany accepted manuscripts. This file will be published in conjunction with your paper and will include the anonymous referee reports, your point-by-point response and all pertinent correspondence relating to the manuscript. Let us know whether you agree with the publication of the RPF and as here, if you want to remove or not any figures from it prior to publication. Please note that the Authors checklist will be published at the end of the RPF.

11) Please provide a point-by-point letter INCLUDING my comments as well as the reviewer's reports and your detailed responses (as Word file).

I look forward to reading a new revised version of your manuscript as soon as possible.

Yours sincerely,

Zeljko Durdevic
Editor
EMBO Molecular Medicine

***** Reviewer's comments *****

Referee #1 (Remarks for Author):

The authors stated: "Consistently, tamoxifen and toremifene were able to reduce Gb3 accumulation and induce TFEB nuclear translocation". However, the authors chose to focus their manuscript on the potential of tamoxifen to treat BD without mentioning in the manuscript why toremifene couldn't also serve this purpose.

The authors did not sufficiently address this comment from my previous review:
Introduction: Please clarify what is meant by "the most common of the rare neurodegenerative disorders in children". Are all childhood forms of neurodegeneration rare?

Referee #2 (Comments on Novelty/Model System for Author):

The model is adequate. Would be great to work with CLN3 Ko mouse models, but the authors clearly struggled with that due to the pandemic.

Referee #2 (Remarks for Author):

The reviewers responded to my comments and improved their manuscript. I still believe that the emphasis on the lysosomal accumulation of GB3 should be also removed from the abstract as it is misleading. They should absolutely state that this is an overall increase in the cell. Being accurate with describing the results is key as there is a fundamental difference between accumulation specific to the lysosome versus an overall increase in GB3 in the cell.

***** Reviewer's comments *****

Referee #1 (Remarks for Author):

The authors stated: "Consistently, tamoxifen and toremifene were able to reduce Gb3 accumulation and induce TFEB nuclear translocation". However, the authors chose to focus their manuscript on the potential of tamoxifen to treat BD without mentioning in the manuscript why toremifene couldn't also serve this purpose.

We already mention in the discussion this possibility. We have said: "Consistently, another lysosomotropic analog of tamoxifen, toremifene, also induces Gb3 clearance and TFEB activation in vitro. Another lysosomotropic SERM, raloxifene (Selyunin et al, 2019), is effective in neuroprotection and immunomodulatory effects in a mouse model of Parkinson's disease (Poirier et al, 2016), supporting the potential benefits of repurposing approved stilbenoids to treat LSDs and more common neurodegenerative disorders.

The authors did not sufficiently address this comment from my previous review: Introduction: Please clarify what is meant by "the most common of the rare neurodegenerative disorders in children". Are all childhood forms of neurodegeneration rare?

Yes, neurodegenerative diseases are rare in children, We have added some relevant BD links in the manuscript such as the NIH source: https://www.ninds.nih.gov/Disorders/Patient-Caregiver-Education/Fact-Sheets/Batten-Disease-Fact-Sheet. In this source is stated: "Although NCL diseases are rare, the childhood onset variants are the most common neurodegenerative disorders of childhood". We have also re-written our sentence in the introduction.

Referee #2 (Comments on Novelty/Model System for Author):

The model is adequate. Would be great to work with CLN3 Ko mouse models, but the authors clearly struggled with that due to the pandemic.

Thanks a lot for understand the situation. We are very excited and interested to move forward this discovery in other BDs.

Referee #2 (Remarks for Author):

The reviewers responded to my comments and improved their manuscript. I still believe that the emphasis on the lysosomal accumulation of GB3 should be also removed from the abstract as it is misleading. They should absolutely state that this is an overall increase in the cell. Being accurate with describing the results is key as there is a fundamental difference between accumulation specific to the lysosome versus an overall increase in GB3 in the cell.

We are very grateful for referee's interest to improve our manuscript with her/him suggestions. We removed lysosomal accumulation from abstract, we stated overall increase of Gb3 in cells and tissues. Also, we have remodulated our claims in the main text.

2nd Revision - Editorial Decision 2nd Aug 2021

We are pleased to inform you that your manuscript is accepted for publication and is now being sent to our publisher to be included in the next available issue of EMBO Molecular Medicine.

EMBO PRESS

YOU MUST COMPLETE ALL CELLS WITH A PINK BACKGROUND ↓

PLEASE NOTE THAT THIS CHECKLIST WILL BE PUBLISHED ALONGSIDE YOUR PAPER

Corresponding Author Name:	
Journal Submitted to:	
Manuscript Number:	

USEFUL LINKS FOR COMPLETING THIS FORM

http://www.antibodypedia.com	Antibodypedia
http://1degreebio.org	1DegreeBio
http://www.equator-network.org/reporting-guidelines/improving-bioscience-research-repor	ARRIVE Guidelines
http://grants.nih.gov/grants/olaw/olaw.htm	NIH Guidelines in animal use
http://www.mrc.ac.uk/Ourresearch/Ethicsresearchguidance/Useofanimals/index.htm	MRC Guidelines on animal use
http://ClinicalTrials.gov	Clinical Trial registration
http://www.consort-statement.org	CONSORT Flow Diagram
http://www.consort-statement.org/checklists/view/32-consort/66-title	CONSORT Check List
http://www.equator-network.org/reporting-guidelines/reporting-recommendations-for-tumo	REMARK Reporting Guidelines (marker prognostic studies)
http://datadryad.org	Dryad
http://figshare.com	Figshare
http://www.ncbi.nlm.nih.gov/gap	dbGAP
http://www.ebi.ac.uk/ega	EGA
http://biomodels.net/	Biomodels Database
http://biomodels.net/miriam/	MIRIAM Guidelines
http://jjj.biochem.sun.ac.za	JWS Online
http://oba.od.nih.gov/biosecurity/biosecurity_documents.html	Biosecurity Documents from NIH
http://www.selectagents.gov/	List of Select Agents

Reporting Checklist For Life Sciences Articles (Rev. June 2017)

This checklist is used to ensure good reporting standards and to improve the reproducibility of published results. These guidelines are consistent with the Principles and Guidelines for Reporting Preclinical Research issued by the NIH in 2014. Please follow the journal's authorship guidelines in preparing your manuscript.

A- Figures

1. Data

The data shown in figures should satisfy the following conditions:

- the data were obtained and processed according to the field's best practice and are presented to reflect the results of the experiments in an accurate and unbiased manner.
- figure panels include only data points, measurements or observations that can be compared to each other in a scientifically meaningful way.
- graphs include clearly labeled error bars for independent experiments and sample sizes. Unless justified, error bars should not be shown for technical replicates.
- if n< 5, the individual data points from each experiment should be plotted and any statistical test employed should be justified
- Source Data should be included to report the data underlying graphs. Please follow the guidelines set out in the author ship guidelines on Data Presentation.

2. Captions

Each figure caption should contain the following information, for each panel where they are relevant:

- a specification of the experimental system investigated (eg cell line, species name).
- the assay(s) and method(s) used to carry out the reported observations and measurements
- an explicit mention of the biological and chemical entity(ies) that are being measured.
- an explicit mention of the biological and chemical entity(ies) that are altered/varied/perturbed in a controlled manner.
- the exact sample size (n) for each experimental group/condition, given as a number, not a range;
- a description of the sample collection allowing the reader to understand whether the samples represent technical or biological replicates (including how many animals, litters, cultures, etc.).
- a statement of how many times the experiment shown was independently replicated in the laboratory.
- definitions of statistical methods and measures:
  - common tests, such as t-test (please specify whether paired vs. unpaired), simple χ2 tests, Wilcoxon and Mann-Whitney tests, can be unambiguously identified by name only, but more complex techniques should be described in the methods section;
  - are tests one-sided or two-sided?
  - are there adjustments for multiple comparisons?
  - exact statistical test results, e.g., P values = x but not P values < x;
  - definition of 'center values' as median or average;
  - definition of error bars as s.d. or s.e.m.

Any descriptions too long for the figure legend should be included in the methods section and/or with the source data.

In the pink boxes below, please ensure that the answers to the following questions are reported in the manuscript itself. Every question should be answered. If the question is not relevant to your research, please write NA (non applicable). We encourage you to include a specific subsection in the methods section for statistics, reagents, animal models and human subjects.

B- Statistics and general methods

Please fill out these boxes ↓ (Do not worry if you cannot see all your text once you press return)

1.a. How was the sample size chosen to ensure adequate power to detect a pre-specified effect size?	For in vitro studies three or more independent experiment were performed. For in vivo studies we used ≥ 3 animals per group.
1.b. For animal studies, include a statement about sample size estimate even if no statistical methods were used.	With the use of InVivoStat software the sample size to achieve a 20% of variation with statistical significance with a power of 100% was enough with 6 animals. For this reason, the number of animals in vivo experiments was at least this number.
2. Describe inclusion/exclusion criteria if samples or animals were excluded from the analysis. Were the criteria pre-established?	for Samples no data exclusion. In the case of animals, the presence of signs of behavioral or health problems was the criteria to exclude animals.
3. Were any steps taken to minimize the effects of subjective bias when allocating animals/samples to treatment (e.g. randomization procedure)? If yes, please describe.	Samples Random allocation . Cultured dishes containing various types of cells, sample preparation, and selection of image fields were randomized for selection in imaging experiments and biochemical assays.
For animal studies, include a statement about randomization even if no randomization was used.	The adscription of animal to each group was done randomly. In the experiment with tamoxifen injection, in the same cage animals were selected arbitrariarly to receive tamoxifen or vehicle.
4.a. Were any steps taken to minimize the effects of subjective bias during group allocation or/and when assessing results (e.g. blinding of the investigator)? If yes please describe.	No formal blinding was done for these assays but several independent participants were performing similar experiments to confirm results.
4.b. For animal studies, include a statement about blinding even if no blinding was done	The analysis of animals samples was completly blind. Samples were identified by numbers and the results were grouped after the analysis. For behavioural tests the animals were tested together, and the results analyzed before.
5. For every figure, are statistical tests justified as appropriate?	YES, Data have been analysed whit the support of the Bioinformatics Core in TIGEM. Statistical tests are described in the figure legends and explained in detail in the Methods section
Do the data meet the assumptions of the tests (e.g., normal distribution)? Describe any methods used to assess it.	Shapiro-Wilk test to check normality assumption. The p-value is not significant, so we can assume normality.
Is there an estimate of variation within each group of data?	Levene's test to check the homogeneity of variances.
Is the variance similar between the groups that are being statistically compared?	The p-value of Levene's test is not significant. This means that, there is not significant difference between variances across groups

C- Reagents

6. To show that antibodies were profiled for use in the system under study (assay and species), provide a citation, catalog number and/or clone number, supplementary information or reference to an antibody validation profile. e.g., Antibodypedia (see link list at top right), 1DegreeBio (see link list at top right).	B-Actin Santa Cruz SC 47778,<br>ULK1 cell signaling cat. 8054<br>Nestin (Thermo Fisher MA1-110 1:200 Phospho-ULK1 (Ser757) cell signaling cat. 6888<br>p70 S6 Kinase cell signaling cat. 2708<br>Phospho-p70 S6 Kinase (Thr389) (cell signaling cat. 9205 1:1000),<br>GAPDH (6C5): (Santa Cruz sc-32233, 1:2000),<br>4EBP (cell signaling cat. 9644 1:1000),<br>p4EBP(cell signaling cat. 9456 1:1000),<br>TFEB (cell signaling cat. 4240S 1:1000)<br>TFEB-pS211 (custom-generated in collaborationwith Bethyl Laboratories 1:1,000).<br>LAMP1 (Santa Cruz cat. sc-20011,1:400),<br>Phospho-S6 Ribosomal Protein (Ser235/236) (cellsignalling cat. 9865 1:400),<br>ATP-C SCMAS (ab181243 Abcam 1:500),<br>CD77/Gb3 (19795 Abcam 1:50), no more available, References: PMID: 30990584; PMID: 30038331; PMID: 27983599; PMID: 26291612<br>HA.11 clone 16B12 Biolegend 901501<br>IBA-1 (019-19741, Wako),<br>NeuN (MAB377 Millipore)<br>GFAP (G6171, Sigma)

	AlexaFluor488-labelled cholera toxin subunit B (C22841 Thermo Fisher Scientific)<br>Filipin III (from Streptomyces filipinensis SIGMA F4767).<br>DRQ5 (62254 Thermo Fisher Scientific)<br>AlexaFluor568-labelled Lysotracker Red (L7528 Thermo Fisher Scientific)<br>AlexaFluor 488 A21202<br>AlexaFluor 488 A11008<br>AlexaFluor 568 A10037<br>AlexaFluor 568 A10042DMEM/F-12, GlutaMAX™ Supplement (Gibco™) (Catalog #31331093)<br>MEM Non-Essential Amino Acids Solution (100X) (Gibco™) (Catalog #11140050)<br>N-2 Supplement (100X) (Gibco™) (Catalog #17502048)<br>B-27™ Supplement (50X), serum free (Gibco™) (Catalog #17504044)<br>Heparin sodium salt from porcine intestinal mucosa (Sigma-Aldrich®) (Catalog #H3393)<br>Recombinant Human FGF-basic (Preprotech®) (Catalog #100-18C)<br>Penicillin-Streptomycin Solution, 50x (Corning®) (Catalog #30-001-CI)<br>Matrigel® hESC-Qualified Matrix, LDEV-free (Corning®) (Catalog #354277)<br>Nestin Monoclonal Antibody (10C2) (ThermoFisher) (Catalog #MA1-110)<br>Recombinant Anti-ATP synthase C antibody [EPR13907] (Abcam) (Catalog #ab181243)<br>Anti-LAMP1 antibody (Abcam) (Catalog #ab24170)<br>Goat anti-Mouse IgG (H+L) Cross-Adsorbed Secondary Antibody, Alexa Fluor 568 (ThermoFisher) (Catalog #A-11004)<br>Goat anti-Rabbit IgG (H+L) Cross-Adsorbed Secondary Antibody, Alexa Fluor 488 (ThermoFisher) (Catalog #A-11008)<br>VECTASHIELD® Mounting Medium for Fluorescencen with DAPI (VECTOR) (Catalog #H-1200)
7. Identify the source of cell lines and report if they were recently authenticated (e.g., by STR profiling) and tested for mycoplasma contamination.	ARPE-19 CLN3 and CLN7 KO were generated by Dr J. Monfregola at TIGEM (Naples). ARPE-19 WT, HeLa wt, U2OS cells were purchased from ATCC .Human control patient fibroblasts were provided by Professor Brunetti (TIGEM), CLN3 patient fibroblasts were purchased from Coriell

* for all hyperlinks, please see the table at the top right of the document

D- Animal Models

8. Report species, strain, gender, age of animals and genetic modification status where applicable. Please detail housing and husbandry conditions and the source of animals.	Mice, CLN7Dex2 (Brandenstein et al., Hum. Mol. Genet. 25:777-91, 2016) y CLN3Dex7/8 (Starapoli et al., PLOS One 7: e38310, 2012) with their respective wild type littermates, in C57Bl6/J genetic background. Only male mice were used. The age of the animals was from 2.5 months to 7.5 months in the case of CLN7 mice, and XXX for CLN3. Animals were bred in cages with five animals maximun per cage, in a light-dark cycle maintained for 12h. The humidity was 45-65%,and the temperature was 20-25ºC. Animals were fed ad libitum with a standard solid diet (17% proteins, 3% lipids, 58.7% carbohydrates, 4.3% cellulose, 5% minerals and 12% humidity) with free access to water. Animal welfare was evaluated during the experiment, and animals were signs of pain were excluded.
9. For experiments involving live vertebrates, include a statement of compliance with ethical regulations and identify the committee(s) approving the experiments.	Animals were bred at the Animal Experimentation Unit of the University of Salamanca. All protocols were performed according to the European Union Directive 86/609/EEC and Recomendation 2007/526/EC, enforced by the Spanish legislation under the law 6/2013. All protocols were approved by the Bioethics Commitee of the University of Salamanca.
10. We recommend consulting the ARRIVE guidelines (see link list at top right) (PLoS Biol. 8(6), e1000412, 2010) to ensure that other relevant aspects of animal studies are adequately reported. See author guidelines, under 'Reporting Guidelines'. See also: NIH (see link list at top right) and MRC (see link list at top right) recommendations. Please confirm compliance.	We have check all.

E- Human Subjects

11. Identify the committee(s) approving the study protocol.	Obtaining human samples and the subsequent use of it was approved by Kaplan Medical Center's Helsinki committee according to standard procedure
12. Include a statement confirming that informed consent was obtained from all subjects and that the experiments conformed to the principles set out in the WMA Declaration of Helsinki and the Department of Health and Human Services Belmont Report.	This is included in the methods section.
13. For publication of patient photos, include a statement confirming that consent to publish was obtained.	NA
14. Report any restrictions on the availability (and/or on the use) of human data or samples.	CLN7 patient fibroblasts were obtained from the UCL patient repository which hold individual patient clinical information that is linked but anonymised. All human data generated in this project is linked to an anonymised patient identifier. There are no restrictions on the availability of the data generated in this study.
15. Report the clinical trial registration number (at ClinicalTrials.gov or equivalent), where applicable.	NA
16. For phase II and III randomized controlled trials, please refer to the CONSORT flow diagram (see link list at top right) and submit the CONSORT checklist (see link list at top right) with your submission. See author guidelines, under 'Reporting Guidelines'. Please confirm you have submitted this list.	NA
17. For tumor marker prognostic studies, we recommend that you follow the REMARK reporting guidelines (see link list at top right). See author guidelines, under 'Reporting Guidelines'. Please confirm you have followed these guidelines.	NA

F- Data Accessibility

18: Provide a "Data Availability" section at the end of the Materials & Methods, listing the accession codes for data generated in this study and deposited in a public database (e.g. RNA-Seq data: Gene Expression Omnibus GSE39462, Proteomics data: PRIDE PXD000208 etc.) Please refer to our author guidelines for 'Data Deposition'.<br><br>Data deposition in a public repository is mandatory for:<br>a. Protein, DNA and RNA sequences<br>b. Macromolecular structures<br>c. Crystallographic data for small molecules<br>d. Functional genomics data<br>e. Proteomics and molecular interactions	N/A
19. Deposition is strongly recommended for any datasets that are central and integral to the study; please consider the journal's data policy. If no structured public repository exists for a given data type, we encourage the provision of datasets in the manuscript as a Supplementary Document (see author guidelines under 'Expanded View' or in unstructured repositories such as Dryad (see link list at top right) or Figshare (see link list at top right).	N/A
20. Access to human clinical and genomic datasets should be provided with as few restrictions as possible while respecting ethical obligations to the patients and relevant medical and legal issues. If practically possible and compatible with the individual consent agreement used in the study, such data should be deposited in one of the major public access-controlled repositories such as dbGAP (see link list at top right) or EGA (see link list at top right).	N/A
21. Computational models that are central and integral to a study should be shared without restrictions and provided in a machine-readable form. The relevant accession numbers or links should be provided. When possible, standardized format (SBML, CellML) should be used instead of scripts (e.g. MATLAB). Authors are strongly encouraged to follow the MIRIAM guidelines (see link list at top right) and deposit their model in a public database such as Biomodels (see link list at top right) or JWS Online (see link list at top right). If computer source code is provided with the paper, it should be deposited in a public repository or included in supplementary information.	N/A

G- Dual use research of concern

22. Could your study fall under dual use research restrictions? Please check biosecurity documents (see link list at top right) and list of select agents and toxins (APHIS/CDC) (see link list at top right). According to our biosecurity guidelines, provide a statement only if it could.	The study we report does not fall under dual use research.
